# Evaluation of the safety and nutritional equivalency of maize grain with genetically modified event DP-Ø23211-2

**DOI:** 10.1080/21645698.2021.1963614

**Published:** 2021-08-29

**Authors:** Brenda L. Smith, Cindi S. Zimmermann, Anne B. Carlson, Carey A. Mathesius, Pushkor Mukerji, James L. McNaughton, Carl A. Walker, Jason M. Roper

**Affiliations:** a Regulatory & Stewardship, Corteva Agriscience^TM^, Johnston, IA, USA; b Regulatory & Stewardship, Corteva Agriscience^TM^, Newark, DE, USA; c President & CEO, AHPharma, Hebron, MA, USA

**Keywords:** DP-ø23211-2 maize grain, genetically modified, rat, broiler chicken, safety, nutritional equivalency

## Abstract

Feeding studies were conducted with rats and broiler chickens to assess the safety and nutrition of maize grain containing event DP-Ø23211-2 (DP23211), a newly developed trait-pyramid product for corn rootworm management. Diets containing 50% ground maize grain from DP23211, non-transgenic control, or non-transgenic reference hybrids (P0928, P0993, and P1105) were fed to Crl:CD®(SD) rats for 90 days. Ross 708 broilers were fed phase diets containing up to 67% maize grain from each source for 42 days. Body weight, gain, and feed conversion were determined for comparisons between animals fed DP23211 and control diets in each study. Additional measures included clinical and neurobehavioral evaluations, ophthalmology, clinical pathology, organ weights, and gross and microscopic pathology for rats, and carcass parts and select organ yields for broilers. Reference groups were included to determine if any observed significant differences between DP23211 and control groups were likely due to natural variation. No diet-related effects on mortality or evaluation measures were observed between animal fed diets produced with DP23211 maize grain and animal fed diets produced with control maize grain. These studies show that maize grain containing event DP-Ø23211-2 is as safe and nutritious as non-transgenic maize grains when fed in nutritionally adequate diets. The results are consistent with previously published studies, providing further demonstration of the absence of hazards from edible-fraction consumption of genetically modified plants.

## Introduction

Animal feeding studies, particularly 90-day subchronic rat studies, using diets containing grains derived from genetically modified (GM) crops continue to be mandated by some regulatory agencies in support of new GM crop event safety assessments.^1–4^ Broiler chickens are a well-established model for GM crop nutritional equivalency studies due to a rapid growth rate (approximately 60-fold growth during a typical 42-day study) and the ability to include high concentrations of maize grains in their diet. Animal feeding studies have consistently confirmed the safety of GM crops shown to be compositionally equivalent to their non-GM counterparts, calling into question the necessity and ethics, of continuing to perform these feeding studies when there are no substantial compositional changes within the crop’s edible fraction (grain and/or forage) of interest.^5,6^ However, it is critical to continue to publish the results of animal feeding studies with GM crops to supplement the overwhelming empirical evidence such that regulatory bodies feel comfortable modifying regulations to align with the scientific evidence.

Event DP-Ø23211-2 maize (DP23211) has been developed as a trait-pyramid product for corn rootworm (CRW; *Diabrotica* spp.) pest management through the expression of DvSSJ1 double-stranded RNA (dsRNA) and IPD072Aa protein. A trait-pyramid product, with multiple modes-of-action against a pest, has greater potential durability (longer time until the target pest develops resistance) when compared with single-mode-of-action products^7–9^ as each mode of action controls insects that are partially or completely resistant to the other mode of action.^10,11^Ingestion of DvSSJ1 dsRNA by western CRW (*Diabrotica virgifera virgifera*), results in suppression of the DvSSJ1 protein in the intestinal lining, leading to subsequent loss of gut epithelium barrier formation and cellular deformities, ultimately resulting in mortality of the CRW.^12^ The expressed IPD072Aa protein, encoded by the *ipd072Aa* gene cloned from a *Pseudomonas chlororaphis* strain,^13^ confers control of CRW pests through disruption of the midgut epithelium. DP23211 maize also expresses the phosphinothricin acetyltransferase (PAT) protein for glufosinate tolerance and the phosphomannose isomerase (PMI) protein is used as a selectable marker. The PAT protein is encoded by the *mo-pat* gene from *Streptomyces viridochromogenes*, and is widely expressed in commercialized GM crops.^14^ The expressed PMI protein is encoded by the *pmi* gene from *Escherichia coli*. This PMI protein is identical to the corresponding protein present in commercialized GM crops.^15^

Compositional equivalence between DP23211 maize and conventional maize has been confirmed.^16^ Two separate feeding studies were conducted to assess the safety and nutrition of DP23211 maize grain as compared with maize grain from its near-isogenic control through diet administration to rats or broilers. The results of these studies are reported herein.

## Materials and Methods

The maize grain and diet characterization analyses and the rat feeding study were conducted in compliance with the US EPA FIFRA (40 CFR part 160) Good Laboratory Practice Standards (GLPs) using validated methods. The broiler study was conducted in compliance with the US FDA GLP for Nonclinical Laboratory Studies (21 CFR part 58; September 1, 2014), with the statistical analysis performed in compliance with the US EPA FIFRA GLPs (40 CFR part 160). Housing and animal care practices for the rat and broiler studies were in accordance with published standards,^17,18^ and the study protocols were reviewed and approved by the respective facility’s animal care and use committee.

### Maize Grain Production & Characterization

All maize grains used in the feeding studies were produced by Corteva Agriscience (Johnston, IA) in a single location during the 2018 growing season. The DP23211 maize grain was produced from plants sprayed with glufosinate (Liberty 280 SL; Bayer CropScience, Research Triangle Park, NC, USA). Control maize grain was obtained from non-transgenic plants with a genetic background similar to DP23211 maize (near-isogenic) and was used to evaluate the gene addition effect. Commercially available non-transgenic hybrids P0928, P0993, and P1105 were included as reference maize grain sources to estimate the expected response range of animals obtained from the same supplier, exposed to the same conditions as those fed diets containing DP23211 or control maize grain, and fed an array of maize safe varieties with a history of safe use. To avoid cross-pollination the control and reference maize plants were produced in plots located 201 m from the DP23211 plot, and neither control nor reference maize plants were sprayed with glufosinate herbicide as they are susceptible to this herbicide. Identity preservation of individual maize grain sources was maintained throughout planting, harvesting, and inventory systems.

The presence of event DP-Ø23211-2 in DP23211 maize grain was confirmed using event-specific qualitative polymerase chain reaction (PCR) analysis, and its absence from control and reference maize grains was confirmed using digital PCR analysis (Corteva Agriscience, Johnston, IA). The DP23211 maize grain was evaluated (Corteva Agriscience, Johnston, IA) for expression of IPD072Aa, PAT, and PMI proteins by ELISA, and DvSSJ1 dsRNA concentration was determined using QuantiGene Plex assay (Thermo Fisher Scientific, Waltham, MA). Samples of all maize grain sources were submitted to EPL Bio Analytical Services, Inc. (EPL-BAS; Niantic, IL) for composition analysis, which included analyses for proximates (dry matter, crude protein, crude fat, ash, and carbohydrates), fiber (crude, acid detergent, neutral detergent), individual amino acids, minerals (calcium, copper, iron, magnesium, manganese, phosphorus, potassium, sodium, and zinc), vitamins (thiamine, riboflavin, folic acid, pyroxidine, niacin, pantothenic acid, and beta carotene), fatty acids, and anti-nutrients and secondary metabolites (inositol, raffinose, *p*-coumaric acid, ferulic acid, trypsin inhibitor, and phytic acid) with analyses conducted as previously described.^19–21^ Additional analyses conducted by EPL-BAS included selenium and contaminant analyses (mycotoxins, pesticides, and PCBs) as previously described,^5^ and gross energy determined by bomb calorimetry (Parr Instruments Model 6200, Parr Instruments, Moline, IL).

### Feeding Study Diet Productions – General

All maize grain sources were ground according to the Corteva Agriscience Standard Operating Procedures, ensuring maize grain particle size of 650–750 microns for each source. Diets in each feeding study were prepared in the order of control, references, and DP23211 to minimize the potential for cross-contamination of control and reference diets with DP23211 maize grain. All equipment was thoroughly cleaned before the use of the first maize source and then between each successive maize source. The confirmation of event DP-Ø23211-2 presence in all diets prepared with DP23211 maize grain and its absence from all diets prepared with control and reference maize grains was performed as described for maize grain (Corteva Agriscience, Johnston, IA). Concentration analyses of IPD072Aa, PAT, and PMI proteins and DvSSJ1 dsRNA in diets with DP23211 maize grain were performed as described for maize grains, with results used in the evaluation of homogeneity and stability. Diet homogeneity was determined based on the PAT protein concentration of samples collected at the beginning, middle, and end of each DP23211 diet production (Corteva Agriscience, Johnston, IA). Stabilities of the expressed IPD072Aa, PAT, and PMI proteins and DvSSJ1 dsRNA were evaluated using diet samples collected during the respective in-life phases of each study as described in the sections that follow.

### Safety Assessment Rat Study

Purina TestDiet (Richmond, IN) formulated and manufactured rat diets based on the nutritional profile of Purina Mills Inc. Certified Rodent LabDiet® 5002^22,23^ with diets formulated to balance crude protein. Ground maize grain was incorporated into each of the five diets at a fixed inclusion rate of 50% by weight for Control, P0928, P0993, and P1105, and DP23211 High; a sixth diet was formulated containing 33% DP23211 maize and 17% control maize (Table 1) to maintain a total maize content of 50% (DP23211 Low). Identities of the grain lots were blinded to diet manufacturing personnel, and diet identities were blinded to feeding study personnel.

**Table 1.** Maize incorporation rates of diets prepared from maize sources for feeding to rats

Maize Grain Lot	Control	DP23211 High	DP23211 Low	P0928	P0993	P1105
Control	50%	---	17%	---	---	---
DP23211	---	50%	33%	---	---	---
P0928	---	---	---	50%	---	---
P0993	---	---	---	---	50%	---
P1105	---	---	---	---	---	50%

Prepared diets were analyzed for nutrient composition and contaminants (EPL BAS Laboratories, Niantic, IL) as described for grains, with the omission of fatty acid and antinutrient analyses, and with the addition of selected metal, mineral, vitamin, and isoflavone analyses. Metals (arsenic, cadmium, cobalt, chromium, mercury, and lead) were analyzed using Inductively Coupled Plasma Mass Spectrometry (ICP-MS)^24^; chloride and fluoride were analyzed using Ion Selective Electrode,^24^ and iodide was analyzed using ICP-MS.^25^ Vitamins A, D3, and choline were analyzed using High-Performance Liquid Chromatography (HPLC)-Mass Spectrometry (MS)/MS^26–28^; vitamin B12 was analyzed using HPLC with Ultraviolet (UV) detection^29,30^; and biotin was analyzed using Ultra-Performance Liquid Chromatography (UPLC)-MS/MS.^31,32^ Isoflavones (daidzin, glycitin, genistin, daidzein, glycitein, and genistein) were analyzed using UPLC with UV Detection.^28,33–35^ Stability samples were collected from the DP23211 High and Low diets on study days 1, 15 and 87.

The feeding study was conducted in compliance with OECD Section 4 (Part 408) test guidelines,^36^ and with European Food Safety Authority (EFSA) guidance for 90-day rat feeding studies.^37,38^ All housing conditions, animal management practices, and acclimation were as previously described.^5^ Male and female Crl:CD®(SD) rats were obtained from Charles River Laboratories, Inc. (Raleigh, NC). Animals (n = 192; 96 males, 96 females) selected for the study following the acclimation period were approximately 7 weeks old at the start of the study and were within ± 20% of the mean weight of each sex. An experimental design of eight cages per treatment and sex (16 rats/sex/treatment) was used in this study, as that has been found to be sufficient in achieving greater than 80% power for detecting targeted effect sizes of biological relevance based on the statistical power analyses required by EFSA for 90-day feeding studies^4,38^ conducted using genetically modified whole food and feed.^37,39^ Assignments to cages and blocks and diet assignments to cages (Supplemental Figure 1) were as previously described.^5^ The body weight mean of each treatment group assessed by sex following randomization was considered acceptable as no statistically significant differences (overall F-Test) among groups were detected. All treatment diets were fed *ad libitum* for a minimum of 90 consecutive days.

Body weight and food consumption data were collected weekly, and food efficiency (weight gain:food consumption) was calculated on a cage-basis. Clinical evaluations were as previously described^39^ and included ophthalmological examinations pre-study and prior to euthanization, and neurobehavioral evaluations [functional observational battery (FOB) and motor activity assessment] prior to study start, and during study days 84 to 87. Detailed weekly clinical observations and daily clinical examinations were also performed. At the study's conclusion, rats were placed in metabolism cages for an overnight (minimum of 15 hours) fast and urine collection. Blood samples were collected on the day of sacrifice for hematology, clinical chemistry, and coagulation analyses; immediately following blood collection, animals were humanely euthanized by inhalation of isoflurane followed by exsanguination. Hematology and coagulation, urinalysis, and clinical chemistry parameters were evaluated as previously described.^5^ Thyroid hormones triiodothyronine (T3) and thyroxine (T4) were analyzed by liquid chromatography-mass spectrometry (Agilent 1290 Infinity UHPLC, Little Falls, DE, and Sciex QTRAP® 6500+ mass spectrometer, Framingham, MA), and thyroid stimulating hormone (TSH) was analyzed by radioimmunoassay (MP Biomedicals, LLC, Solon, OH).

Necropsy examinations for all animals included the external surface, all orifices, and the cranial, thoracic, abdominal, and pelvic cavities, including viscera. Absolute and relative (to terminal body and brain weights) organ weights were recorded, and tissues were collected and preserved as previously described^5^; preserved tissues were evaluated microscopically by a veterinary pathologist, and peer-reviewed by a second veterinary pathologist.

Data summarization and statistical analysis, including application of false discovery rate (FDR) to control for multiplicity, were all as previously described^5^ with endpoints for rats fed either DP23211 High or DP23211 Low diets statistically compared with endpoints of those fed the Control diet both across sex (when possible) and within sex. The importance of adjusting for multiplicity has been recognized in EFSA guidance^37^ and investigated in published research.^39^

### Nutritional Equivalency Broiler Study

All diets were prepared at the Corteva Agriscience Regulatory Science Grain Facility (Polk City, IA). A three-phase feeding program was used with mash-type diets offered *ad libitum* in starter (days 0 to 21), grower (days 22 to 35), and finisher (days 36 to 42) phases. Diets within each phase were formulated to meet nutrient requirements of a typical commercial broiler diet using the NRC 1994^40^ requirements as a guideline and taking into account commercial formulation practices that have progressed since NRC publication. Maize grain inclusion was equalized across treatments (Control, DP23211, P0928, P0993, and P1105) within each phase with concentrations of non-maize ingredients adjusted to meet and equalize essential nutrient (protein, lysine, methionine, cystine, calcium, and phosphorus) requirements within a phase (Table 2). Duplicate samples of each prepared diet were submitted for proximates, mineral (calcium and phosphorus only), amino acid, and gross energy analyses as previously described for grain samples (EPL BAS Laboratories, Niantic, IL). Stability samples were collected from DP23211 diets at individual phase beginning and end.

**Table 2.** Ingredient composition of phase^a^ diets prepared from maize sources for feeding to broilers

Ingredient, %	Control	DP23211	P0928	P0993	P1105
	Starter Phase				
Maize	63.500	63.500	63.500	63.500	63.500
Soybean meal	21.558	23.943	20.356	20.823	20.218
Protein blend^b^	7.952	6.583	9.934	9.341	10.123
Soybean oil	2.454	1.492	1.812	1.907	1.758
Sodium chloride	0.471	0.476	0.463	0.465	0.462
Limestone	1.370	1.405	1.280	1.302	1.280
Dicalcium phosphate	1.906	1.906	1.971	1.961	1.950
VM premix^c^	0.250	0.250	0.250	0.250	0.250
Choline chloride	0.011	–	0.017	0.015	0.017
DL-Methionine	0.240	0.208	0.153	0.174	0.177
L-Lysine-HCL	0.287	0.236	0.265	0.262	0.263
	Grower Phase				
Maize	67.000	67.000	67.000	67.000	67.000
Soybean meal	19.888	22.377	18.633	19.120	18.489
Protein blend	6.159	4.731	8.242	7.620	8.441
Soybean oil	2.911	1.896	2.234	2.334	2.177
Sodium chloride	0.429	0.434	0.420	0.422	0.419
Limestone	1.250	1.287	1.155	1.179	1.156
Dicalcium phosphate	1.617	1.617	1.685	1.675	1.663
VM premix	0.250	0.250	0.250	0.250	0.250
DL-Methionine	0.184	0.151	0.093	0.115	0.118
L-Lysine-HCL	0.312	0.259	0.288	0.285	0.287
	Finisher Phase				
Maize	64.000	64.000	64.000	64.000	64.000
Soybean meal	24.923	27.181	23.724	24.190	23.587
Protein blend	1.931	0.636	3.922	3.327	4.112
Soybean oil	5.449	4.478	4.802	4.898	4.748
Sodium chloride	0.396	0.401	0.388	0.391	0.387
Limestone	1.367	1.400	1.276	1.299	1.277
Dicalcium phosphate	1.432	1.433	1.497	1.487	1.475
VM premix	0.250	0.250	0.250	0.250	0.250
Choline chloride	–	0.051	–	–	–
DL-Methionine	0.203	0.170	0.115	0.136	0.139
L-Lysine-HCL	0.049	–	0.026	0.023	0.025

^a^Starter phase diets were formulated to contain: metabolizable energy, 3175 kcal/kg; protein, 22.5%; lysine, 1.28%; methionine + cystine, 1.07%; and calcium, 1.00%. Grower phase diets were formulated to contain: metabolizable energy, 3197 kcal/kg; protein, 20.5%; lysine, 1.21%; methionine + cystine, 0.93%; and calcium, 0.88%. Finisher phase diets were formulated to contain: metabolizable energy, 3208 kcal/kg; protein, 19.0%; lysine, 1.00%; methionine + cystine, 0.85%; and calcium, 0.85%.

^b^Protein blend was manufactured by Papillion Agricultural Company (Easton, MD, USA). Analyzed composition (as-fed basis): moisture, 6.8%; protein, 81.332%; gross energy, 5151 kcal/kg; lysine, 2.78%; methionine, 0.556%; methionine+cystine, 3.926%; tryptophan, 0.437%; threonine, 3.71%; and arginine, 5.44%.

^c^Vitamin-trace mineral premix supplied (minimum) per kg diet: iron, 115 mg; manganese, 88 mg; zinc, 88 mg; copper, 12 mg; iodine, 1.3 mg; selenium, 0.3 mg; vitamin A, 13,750 IU; vitamin D_3_, 4,620 IU; vitamin E, 28 IU; vitamin B_12_, 0.016 mg; menadione, 4.7 mg; riboflavin, 9.4 mg; D-pantothenic acid, 15 mg; niacin, 85 mg; thiamine, 1.1 mg; folic acid, 0.6 mg; vitamin B_12_, 3.3 mg; and biotin, 0.06 mg.

AHPharma, Inc. (Hebron, MD) incubated and hatched Ross 708 commercial broiler eggs (Longenecker’s Hatchery, Inc., Elizabethtown, PA) and transported the chicks on the day of hatch (day 0) to their farm facility where they were evaluated for signs of disease or other complications. Broilers deemed healthy for the conduct of the study were weighed individually, identified with a wing band, and assigned to the five dietary treatments in a randomized complete block design with 10 broilers per pen and 12 pens (6 males and 6 females) per treatment. Treatment group weight distribution assessed prior to feeding was considered acceptable as differences were within one standard deviation. All housing conditions and animal management practices were as previously described.^6^ Broilers were fed their respective dietary treatments from the day of hatching (day 0) to 42 days of age. Determination of body weights and feed intakes were made every 7 days to calculate cumulative (days 0 to 42) body weight gain, feed intake, and mortality-corrected feed conversion ratio. All surviving birds selected for carcass data collection were humanely euthanized by exsanguination with complete severing of the spinal column on day 42 with carcass, individual carcass-parts (breast, thigh, wing, leg, and abdominal fat), and organ (kidney, liver) yielded data collected from seven broilers per pen. Data summary and statistical analysis were as previously described^6^ with endpoints for broiler fed DP23211 diet statistically compared with endpoints of those fed the Control diet. As there were no significant treatment x sex interactions observed for any trait, the combined-sex analysis was interpreted for all measures.

## Results and Discussion

### Grain Characterization Results

Event DP-Ø23211-2 presence in DP23211 maize and its absence from control and reference maize sources was confirmed. Expression levels of IPD072Aa, PAT, and PMI proteins in DP23211 maize grain were determined to be 1.6, 2.8, and 2.5 ng/mg dry weight, respectively, and DvSSJ1 dsRNA was present at 3.55 pg/mg dry weight. Analyzed nutrient values were similar among all maize grain sources (Table 3, Supplemental Table S1) and fell within reference ranges for proximates, fiber, amino acids, fatty acids, most vitamins, and minerals.^41–43^ Lower β-carotene levels in DP23211, control, and P1105 maize grains were not a concern as vitamin premixes are typically included in diet formulations to ensure species-specific dietary requirements. Fumonisins (FB_1_, FB_2_, FB_3_), deoxynivalenol, moniliformin, and zearalenone (Supplemental Table S1) were present in some grain lots, but at concentrations below the recommended maximum dietary levels and/or the respective no observed effect levels.^44–50^ Pesticide and PCB residues were not detected in any grain source. All maize grain sources were considered suitable for rat and broiler diet production as no differences in key nutrients that would have impacted inclusion rates were observed between the grain sources, and no contaminants, anti-nutrients, or metabolites were present in quantities sufficient to preclude grain source use.

**Table 3.** Analyzed nutrient profiles^a^ (as-is basis) of maize sources are used to prepare diets for rat and broiler feeding studies

Nutrient	Control	DP23211	P0928	P0993	P1105
Proximates and fibers, %					
Moisture	15.5	15.3	15.8	15.8	16.0
Protein	8.245	8.325	6.778	7.158	6.603
Fat	3.50	3.52	3.62	3.77	3.75
Crude fiber	2.06	1.98	2.03	2.11	2.05
Ash	1.27	1.23	1.17	1.13	1.29
ADF	3.57	3.42	3.36	3.57	3.47
NDF	8.45	8.15	8.23	8.92	9.20
Carbohydrates	71.5	71.6	72.7	72.2	72.4
Gross energy, kcal/kg	3,745	3,862	3,717	3,733	3,716
Metabolizable energy, kcal/kg	3,296	3,399	3,271	3,285	3,270
Minerals, % (unless otherwise indicated)					
Calcium	0.00243	0.00227	0.00241	0.00254	0.00332
Phosphorus	0.312	0.287	0.259	0.264	0.278
Magnesium	0.0937	0.0900	0.0739	0.0737	0.0688
Potassium	0.283	0.267	0.273	0.277	0.305
Sodium	0.000252	<0.0000625	0.000980	0.00412	0.000304
Zinc	0.00164	0.00152	0.00143	0.00142	0.00148
Manganese	0.000517	0.000455	0.000339	0.000341	0.000399
Copper	0.000213	0.000214	0.000164	0.000172	0.000257
Iron	0.00217	0.00241	0.00180	0.00180	0.00144
Selenium (ppm)	0.160	<0.100	0.351	0.315	0.171
Amino acids, %					
Alanine	0.620	0.635	0.522	0.524	0.480
Arginine	0.396	0.367	0.371	0.344	0.338
Aspartic acid	0.520	0.562	0.479	0.487	0.453
Cystine	0.170	0.200	0.186	0.186	0.171
Glutamic acid	1.60	1.68	1.32	1.35	1.21
Glycine	0.349	0.326	0.332	0.308	0.297
Histidine	0.289	0.262	0.238	0.228	0.237
Isoleucine	0.307	0.300	0.259	0.249	0.237
Leucine	1.09	1.07	0.851	0.824	0.757
Lysine	0.229	0.248	0.232	0.240	0.232
Methionine	0.133	0.179	0.158	0.151	0.127
Phenylalanine	0.505	0.448	0.410	0.365	0.351
Proline	0.804	0.770	0.659	0.628	0.561
Serine	0.441	0.433	0.373	0.365	0.347
Threonine	0.327	0.324	0.294	0.286	0.271
Tryptophan	0.0634	0.0657	0.0651	0.0646	0.0637
Tyrosine	0.277	0.241	0.255	0.219	0.208
Valine	0.394	0.389	0.350	0.339	0.318
Fatty Acids, % total fatty acids					
C16:0, Palmitic acid	13.2	13.1	12.6	12.6	12.1
C16:1, Palmitoleic acid	0.102	0.101	0.112	0.116	0.103
C18:0, Stearic acid	1.70	1.75	1.64	1.67	1.71
C18:1, Oleic acid	20.4	20.4	20.3	20.4	21.0
C18:2, Linoleic acid	59.1	58.9	60.3	59.4	60.2
C18:2 (9,15)	3.06	3.20	2.26	2.96	2.24
C18:3, Linolenic acid	1.44	1.38	1.46	1.47	1.52
C20:0, Arachidic acid	0.353	0.359	0.387	0.403	0.374
C20:1, Eicosenoic acid	0.309	0.318	0.285	0.293	0.285
C22:0, Behenic acid	0.182	0.181	0.204	0.217	0.199
C24:0, Lignoceric acid	0.248	0.238	0.280	0.293	0.255
Vitamins, mg/100 g (unless otherwise indicated)					
Folic Acid	0.134	0.103	0.161	0.148	0.0671
Niacin	0.959	1.01	0.985	1.08	0.968
Thiamine	0.270	0.227	0.217	0.206	0.214
Riboflavin	<0.0900	<0.0900	<0.0900	<0.0900	<0.0900
Pyridoxine	0.239	0.205	0.256	0.281	0.244
Pantothenic acid	0.576	0.594	0.562	0.558	0.594
Tocopherols (Total)	2.41	2.51	1.93	2.20	3.15
Alpha	0.389	0.401	1.04	1.17	1.36
Beta	<0.0500	<0.0500	<0.0500	<0.0500	0.0563
Delta	0.0585	0.0829	<0.0500	<0.0500	0.0738
Gamma	1.91	1.98	0.785	0.930	1.66
Beta Carotene (mg/kg)	0.117	0.136	0.373	0.373	0.119

^a^Broiler diet formulation used only proximates, energy, amino acids, and calcium and phosphorus values. Metabolizable energy values were calculated from gross energy values using conversion factors based upon internal Corteva Agriscience data.

### Diet Characterizations and Compositions

The presence of event DP-Ø23211-2 in all DP23211 test diets and its absence from all control and reference diets used in each feeding study was confirmed (data not shown). Nutrient analyses demonstrated that all prepared rat and broiler diets were nutritionally adequate and suitable for use in the respective feeding studies (Supplemental Tables S2 [rat] and S3 [broiler]). All diets produced with DP23211 maize grain were blended homogeneously as indicated by PAT protein concentration, and the expressed IPD072Aa, PAT, and PMI proteins and DvSSJ1 dsRNA were stable for the duration of the respective diet exposures (data not shown).

### Safety Assessment Rat Study

## In-life Assessments

There were no deaths related to DP23211 consumption in this study. One female fed P0928 reference diet was found dead on day 11 with the cause of death undetermined as no significant gross findings or histopathologic findings were present. There were no diet-related effects on body weight or weight gain between DP23211 High or DP23211 Low groups and the Control group nor were there any statistically significant differences after application of FDR adjustment (Table 4; Supplemental Table S4). A similar lack of diet-related effects and statistically significant differences in post-FDR adjustment were observed with food consumption and efficiency measures (Table 4; Supplemental Table S5). There were no diet-related effects on clinical observations or ophthalmology measures (Supplemental Table S6), nor were forelimb and hindlimb grip strengths affected by diet (Supplemental Table S7). There were no diet-related effects on movement duration (Supplemental Table S7) when data from the DP23211 High and DP23211 Low test groups were compared with data from the Control group, and there were no statistically significant differences between either test and Control group after FDR adjustment was applied. No diet-related effects on the number of movements were observed between DP23211 High or DP23211 Low test and the Control group, and there were no statistically significant differences between the DP23211 High and Control groups following FDR adjustment.

**Table 4.** Body weight and weight gain, food consumption, and food efficiency values^a,b^ of rats fed diets containing non-transgenic maize grain or diets containing DP23211 maize grains

Item	Control	DP23211 High	DP23211 Low	P0928	P0993	P1105
Body weight (g), day 91						
Females	313.2	298.2	309.3	302.3	303.5	309.4
95% CI^c^	294.4 - 332.0	279.4 - 317.0	290.5 - 357.1			
Males	613.9	601.4	624.1	584.2	597.9	592.8
95% CI	583.9 - 643.9	571.4 - 631.4	594.1 - 654.1			
Body weight gain (g), day 1 to day 91						
Females	142.4	128.2	139.1	128.7	131.7	134.9
95% CI	131.2 - 153.7	116.9 - 139.5	127.9 - 150.4			
Males	363.9	351.9	374.8	335.8	347.7	342.6
95% CI	337.4 - 390.3	325.4 - 378.4	348.3 - 401.2			
Mean food consumption (g/day), day 1 to day 91						
Females	18.6	19.2	18.9	18.5	18.5	19.3
95% CI	17.3 - 19.9	17.9 - 20.6	17.6 - 20.2			
Males	28.8	29.2	29.2	27.3	28.6	27.4
95% CI	27.5 - 30.1	27.9 - 30.5	27.9 - 30.5			
Food efficiency (g/g)^d^, day 1 to day 91						
Females	0.085	0.075*	0.082	0.077	0.079	0.078
95% CI	0.080 - 0.090	0.069 - 0.080	0.076 - 0.087			
Males	0.140	0.134	0.143	0.137	0.136	0.139
95% CI	0.133 - 0.148	0.126 - 0.141	0.135 - 0.150			

^a^Mean body weight and weight gain: n = 16 for all treatments except P0928 where n = 15.

^b^Mean food consumption and food efficiency: n = 8 for all treatments except P0928 where n = 7.

^c^Confidence Interval (CI) around observed Control, DP23211 High, and DP23211 Low means.

^d^Food efficiency was calculated as average daily weight gain (g) / average daily food consumption (g).

*Statistically significant difference (P < 0.05) when compared to Control; FDR P value not significantly different (P > 0.05).

### Clinical and Anatomic Pathology

There were no diet-related effects on hematology or clinical chemistry parameters when DP23211 High or DP23211 Low groups were compared with the Control group (Supplemental Tables S8 and S10, respectively), nor were there any observed statistically significant differences after application of FDR adjustment. There were no diet-related effects on coagulation, hormone, or urinalysis parameters (Supplemental Tables S9 – S11). There were no diet-related effects on organ weight parameters (Supplemental Table S12) when data from DP23211 High or DP23211 Low groups were compared with data from the Control group, nor were there any statistically significant differences after FDR adjustment was applied. There were no diet-related microscopic findings (Supplemental Table S13); all microscopic observations were considered to be background findings not associated with consumption of DP23211 High or DP23211 Low diets.

The lack of diet-related or significant effects on growth and pathology evaluations indicates that maize grain containing event DP-Ø23211-2 is as safe and nutritious as maize grain not containing event DP-Ø23211-2. Other recently published 90-day subchronic feeding studies have also demonstrated that diets containing GM maize were as safe and nutritious as those containing non-GM maize.^5,51,52^

### Nutritional Equivalency Broiler Study

## In-life Growth Performance

As their rapid weight gain and mortality are sensitive indicators of changes in diet nutritional quality, broiler studies are designed to look for adverse effects on performance due to unexpected compositional changes in the GM crop. Mortality did not differ between the DP23211 and Control treatment groups and the values of those groups were similar to the observed reference range values (Table 5). There were no significant differences (*P* > .05) in body weights, cumulative body weight gains (days 0 to 42), or mortality-adjusted feed:gain ratios between broilers consuming diets produced with DP23211 maize grain and those consuming diets produced with control maize grain (Table 5).

**Table 5.** Body weights and gains, feed efficiency, and mortality values^a^ of broilers fed diets containing non-transgenic maize grains or diets containing DP23211 maize grains

Item	Control	DP23211	Difference with 95% CI^b^	Reference Maize Groups^c^
Initial weight (g), day 0	41.9	42.0	0.1 (-0.5, 0.8)	36.7 - 47.7
	(36.9 - 47.5)	(36.7 - 47.5)		
Females	41.6	41.6	0.0 (-0.8, 0.9)	36.7 - 46.9
	(36.6 - 46.6)	(36.7 - 46.7)		
Males	42.2	42.5	0.2 (-0.6, 1.1)	37.0 - 47.7
	(36.9 - 47.5)	(37.2 - 47.5)		
Final weight (g), day 42	2459.7	2458.2	-1.5 (-50.5, 47.6)	1713.5 - 3217.6
	(1690.7 - 3192.9)	(1784.4 - 3154.4)		
Females	2295.8	2307.5	11.7 (-58.0, 81.5)	1713.5 - 3176.2
	(1690.7 - 3107.2)	(1830.6 - 3154.4)		
Males	2623.7	2609.0	-14.7 (-83.7, 54.3)	1847.8 - 3217.6
	(2234.6 - 3192.9)	(1784.4 - 3144.1)		
Body weight gain (g), day 0 to day 42	2417.8	2416.2	-1.6 (-50.4, 47.3)	1675.5 - 3169.9
	(1653.8 - 3146.3)	(1745.7 - 3111.2)		
Females	2254.2	2266.0	11.8 (-57.6, 81.3)	1675.5 - 3130.6
	(1653.8 - 3061.1)	(1792.4 - 3111.2)		
Males	2581.4	2566.4	-15.0 (-83.7, 53.7)	1804.9 - 3169.9
	(2194.8 - 3146.3)	(1745.7 - 3097.9)		
FCR (g/g), day 0 to day 42^d^	1.752	1.747	-0.005 (-0.040, 0.031)	1.686 - 1.809
	(1.688 - 1.823)	(1.678 - 1.814)		
Females	1.744	1.758	-0.014 (-0.035, 0.064)	1.690 - 1.809
	(1.688 - 1.820)	(1.689 - 1.800)		
Males	1.760	1.736	-0.024 (-0.073, 0.026)	1.686 - 1.806
	(1.688 - 1.823)	(1.678 - 1.814)		
Mortality (%)	4.17	3.33	---	3.33 - 4.17
Females	5.00	3.33	---	3.33 - 5.00
Males	3.33	3.33	---	1.67 - 5.00

^a^Overall (combined gender) treatment growth performance means represent 12 pens per treatment group with 10 birds/pen; female and males means represent 6 pens each per treatment. Value in () is the range of observed values for that treatment.

^b^Confidence Interval (CI) of observed difference between DP23211 and Control treatment groups

^c^Range of values observed across all reference commercial maize treatment groups (P0928, P0993, and P1105).

^d^Feed Conversion Ratio (FCR) is calculated as g of feed intake per g of body weight gain and was adjusted for mortality.

### Organ and Carcass Yields

Organ yields, such as those of the liver and kidneys, may be indicative of negative effects on broiler health due to dietary inadequacies^53–55^ or the presence of antinutritional factors.^56–59^ There were no significant differences in organ yields (*P* > .05) between broilers consuming DP23211 diets and those consuming Control diets in this study (Table 6). There were no significant differences (*P* > .05) in carcass yield, as expressed as the percentage of whole live bird weight, between broilers consuming DP23211 diets and broilers consuming Control diets (Table 6). A similar lack of diet-related effects and statistically significant differences in post-FDR adjustment was observed with individual carcass parts yields, expressed as the percentage of post-chilled dressed carcass weight (Table 6).

**Table 6.** Pre-chill organ yields and post-chill carcass and parts yields^a^ of broilers fed diets containing non-transgenic controlled maize grains or diets containing DP23211 maize grains

**Item**	**Control**	**DP23211**	**Difference with** **95% CI^b^**	**Reference Maize** **Groups^c^**
Pre-chill organ yields				
Kidney (%)	1.16	1.16	-0.01 (-0.13, 0.12)	0.55 - 1.84
	(0.55 - 1.82)	(0.56 - 1.83)		
Females	1.15	1.20	0.05 (-0.13, 0.22)	0.56 - 1.83
	(0.63 - 1.82)	(0.60 - 1.83)		
Males	1.17	1.11	-0.06 (-0.24, 0.12)	0.55 - 1.79
	(0.55 - 1.79)	(0.56 - 1.83)		
Liver (%)	2.77	2.68	-0.09 (-0.26, 0.07)	1.76 - 3.66
	(1.82 - 3.63)	(1.77 - 3.67)		
Females	2.79	2.68	-0.11 (-0.35, 0.12)	1.77 - 3.66
	(1.82 - 3.60)	(1.77 - 3.67)		
Males	2.75	2.68	-0.07 (-0.30, 0.16)	1.76 - 3.64
	(1.83 - 3.63)	(1.79 - 3.62)		
Post-chill carcass and parts yields				
Carcass (%)	73.12	73.16	0.24 (-1.07, 1.54)	65.82 - 79.46
	(66.24 - 79.54)	(66.13 - 79.54)		
Females	72.30	73.54	1.24 (-0.31, 2.79)	66.37 - 79.33
	(66.24 - 78.90)	(66.14 - 79.54)		
Males	73.94	72.79	-1.15 (-2.70, 0.40)	65.82 - 79.46
	(66.91 - 79.54)	(66.13 - 79.45)		
Breast (%)	24.11	24.14	0.03 (-0.89, 0.95)	18.92 - 29.20
	(18.69 - 29.20)	(19.06 - 30.28)		
Females	24.28	24.52	0.24 (-1.07, 1.54)	18.92 - 29.75
	(19.72 - 28.76)	(19.06 - 30.28)		
Males	23.94	23.77	-0.17 (-1.47, 1.13)	19.93 - 29.87
	(18.69 - 29.20)	(19.26 - 29.84)		
Thigh (%)	16.56	16.84	0.28 (-0.31, 0.87)	13.02 - 21.48
	(12.68 - 20.05)	(13.24 - 20.48)		
Females	16.64	16.69	0.05 (-0.78, 0.88)	13.02 - 21.17
	(13.17 - 19.37)	(13.24 - 19.97)		
Males	16.48	16.99	0.51 (-0.32, 1.34)	13.16 - 21.48
	(12.68 - 20.05)	(13.76 - 20.48)		
Leg (%)	14.10	14.06	-0.04 (-0.43, 0.35)	11.25 - 17.16
	(11.63 - 16.88)	(11.42 - 16.93)		
Females	14.27	13.96	-0.31 (-0.86, 0.24)	11.25 - 17.16
	(11.76 - 16.88)	(11.42 - 16.93)		
Males	13.94	14.17	0.23 (-0.31, 0.78)	11.25 - 16.71
	(11.63 - 16.87)	(11.74 - 16.46)		
Wing (%)	10.74	11.06*	0.31 (0.01, 0.62)	8.59 - 13.54
	(8.80 - 13.41)	(8.64 - 13.57)		
Females	10.80	10.86	0.07 (-0.37, 0.50)	8.59 - 13.54
	(8.80 - 12.81)	(8.64 - 13.57)		
Males	10.69	11.25	0.56 (0.13, 1.00)	9.18 - 13.41
	(8.85 - 13.41)	(8.92 - 13.26)		
Abdominal fat (%)	1.42	1.51	0.08 (-0.07, 0.23)	0.68 - 2.48
	(0.67 - 2.40)	(0.68 - 2.51)		
Females	1.48	1.55	0.07 (-0.14, 0.28)	0.68 - 2.44
	(0.75 - 2.32)	(0.76 - 2.42)		
Males	1.37	1.46	0.10 (-0.12, 0.31)	0.71 - 2.48
	(0.67 - 2.40)	(0.68 - 2.51)		

^a^Pre-chill organ and carcass yields were calculated as percent of live bird weight; parts yield was calculated as percent of post-chill carcass weight. Overall (combined gender) treatment means represent 12 pens per treatment group with 7 birds/pen; female and males means represent 6 pens each per treatment 7 birds/pen. Value in () is the range of observed values for that treatment.

^b^Confidence Interval (CI) of observed difference between DP23211 and Control treatment groups.

^c^Range of values observed across all reference commercial maize treatment groups (P0928, P0993, and P1105).

*Statistically significant difference (P < 0.05) when compared to Control; FDR P value not significantly different (P > 0.05).

Other researchers have similarly reported a lack of diet-related or biologically significant differences in growth performance, organ yields, and carcass or individual parts yields between broilers fed diets formulated with GM maize grains and broilers fed diets formulated with non-GM near-isogenic control maize grains.^6,60–65^ The observed lack of effects in this study demonstrates that DP23211 maize grain is as safe and nutritious as maize grain not containing event DP-Ø23211-2.

## Conclusions

The results of the subchronic rat feeding study and of the broiler grow-out study confirm the nutritional equivalence and safety to conventional maize observed in the direct compositional analyses of DP23211 maize.^16^ The consistency of these results with, and their addition to the already-extensive literature that exists on GM animal feeding studies should aid authorities and policy-makers in the modernization of animal-use regulations, thus reducing mandatory animal research requirements in favor of hypothesis-based investigations of factors that have a true potential to impact health and safety.^66,67^

## Supplementary Material

Supplemental Material
