# Supplementary material for: Evaluation of the safety and nutritional equivalency of maize grain with genetically modified event DP-Ø23211-2
Source: GM Crops Food. 2021 Aug 29;12(1):396–408. doi: 10.1080/21645698.2021.1963614 (PMC8409786; doi:10.1080/21645698.2021.1963614)
Supplement: Supplemental Material [file KGMC_A_1963614_SM3631.zip › DP23211 Manuscript Supplemental Information.doc]

Evaluation of the Safety and Nutritional Equivalency of Maize Grain with Genetically Modified Event

DP-Ø23211-2

Brenda L. Smith*^1^, Cindi S. Zimmermann*, Anne B. Carlson*, Carey A. Mathesius*, Pushkor Mukerji^†^, James L. McNaughton^‡^, Carl A. Walker*, and Jason M. Roper^†^

*Corteva AgriscienceTM, 7300 NW 62nd Ave, Johnston, Iowa 50131, USA

^†^Corteva AgriscienceTM, 1090 Elkton Road, Newark, Delaware 19714, USA

^‡^AHPharma, 27013 East Lillian St, Hebron, Maryland, 21830

^1^Corresponding author: Brenda.Smith@corteva.com

| Male | | Column | | | | | |  | Female | | Column | | | | | |
| --- | --- | --- | --- | --- | --- | --- | --- | --- | --- | --- | --- | --- | --- | --- | --- | --- |
| Rack | Row | 1 | 2 | 3 | 4 | 5 | 6 |  | Rack | Row | 1 | 2 | 3 | 4 | 5 | 6 |
| 1 | 1 | X | * | X | X | X | X |  | 3 | 1 | X | X | X | X | X | X |
|  | 2 | 6 | 1 | 2 | 1 | 4 | 2 |  |  | 2 | 6 | 5 | 3 | 2 | 6 | 2  Block 1  Block 3  Block 1  Block 3 |
|  | 3 | 4 | 5* | 3 | 3 | 5 | 6 |  |  | 3 | 2 | 1 | 4 | 4 | 5 | 1 |
|  | 4 | 1 | 6 | 2 | 3 | 2 | 5 |  |  | 4 | 3 | 5 | 1 | 2 | 3 | 5  Block 2  Block 4  Block 2  Block 4 |
|  | 5 | 5 | 3 | 4 | 1 | 4 | 6 |  |  | 5 | 4 | 2 | 6 | 1 | 6 | 4 |
|  | 6 | X | X | X | X | X | X |  |  | 6 | X | X | X | X | X | X |
|  |  |  |  |  |  |  |  |  |  |  |  |  |  |  |  |  |
| Male | | Column | | | | | |  | Female | | Column | | | | | |
| Rack | Row | 1 | 2 | 3 | 4 | 5 | 6 |  | Rack | Row | 1 | 2 | 3 | 4 | 5 | 6 |
| 2 | 1 | X | X | X | X | X | X |  | 4 | 1 | X | X | X | X | X | X |
|  | 2 | 1  Block 7  Block 5  Block 7 | 5 | 4 | 6 | 1 | 2 |  |  | 2 | 1  Block 5 | 2 | 3 | 5 | 3 | 1 |
|  | 3 | 6 | 3 | 2 | 4 | 5 | 3 |  |  | 3 | 6 | 4 | 5 | 2 | 6 | 4 |
|  | 4 | 2  Block 6 | 1 | 4 | 5  Block 8 | 6 | 4 |  |  | 4 | 4  Block 6 | 5 | 2 | 4  Block 8 | 6 | 1 |
|  | 5 | 6 | 3 | 5 | 2 | 3 | 1 |  |  | 5 | 1 | 3 | 6 | 3 | 5 | 2 |
|  | 6 | X | X | X | X | X | X |  |  | 6 | X | X | X | X | X | X |

**Figure S1. Arrangement of Cages on Racks Utilized in Rat Feeding Study**

Numbers within each block denote the diet/group code (1-6) for each cage. Unused but available cage positions are represented by ‘X’.

*One male from the DP23211 High group was removed from its original cage on day 84 due to injury likely resulting from cagemate fighting and was housed in a new cage in the closest available empty space for the rest of the study; its cagemate remained in the original cage.

**Table S1. Analyzed Fatty Acid, Anti-nutrient, and Mycotoxin Profiles (as-is basis) of Maize Grain Sources**

| **Item** | **Control** | **DP23211** | **P0928** | **P0993** | **P1105** |
| --- | --- | --- | --- | --- | --- |
| Fatty acid^1^, % total fatty acid | | | | | |
| Palmitic (C16:0) | 13.2 | 13.1 | 12.6 | 12.6 | 12.1 |
| Palmitoleic (C16:1) | 0.102 | 0.101 | 0.112 | 0.116 | 0.103 |
| Heptadecanoic (C17:0) | 0 | 0.0907 | 0.0929 | 0.0962 | 0.103 |
| Stearic (C18:0) | 1.70 | 1.75 | 1.64 | 1.67 | 1.71 |
| Oleic (C18:1) | 20.4 | 20.4 | 20.3 | 20.4 | 21 |
| Linoleic (C18:2) | 59.1 | 58.9 | 60.3 | 59.4 | 60.2 |
| Linolenic (C18:3) | 1.44 | 1.38 | 1.46 | 1.47 | 1.52 |
| C18:2 (9,15) | 3.06 | 3.20 | 2.26 | 2.96 | 2.24 |
| Nonadecenoic (C19:1) | 0.35 | 0.36 | 0.39 | 0.40 | 0.37 |
| Arachidic (C20:0) | 0.353 | 0.359 | 0.387 | 0.403 | 0.374 |
| Eicosenoic (C20:1) | 0.309 | 0.318 | 0.285 | 0.293 | 0.285 |
| Behenic (C22:0) | 0.182 | 0.181 | 0.204 | 0.217 | 0.199 |
| Lignoceric (C24:0) | 0.248 | 0.238 | 0.280 | 0.293 | 0.255 |
|  | | | | | |
| Anti-nutrients and secondary metabolites^2^, % (unless indicated otherwise) | | | | | |
| Phytic acid | 1.05 | 0.834 | 0.739 | 0.835 | 0.872 |
| Raffinose | 0.200 | 0.234 | 0.191 | 0.188 | 0.160 |
| Inositol | < 0.00400 | 0.00422 | 0.00600 | 0.00497 | 0.00631 |
| Trypsin inhibitor activity (TIU/g) | 2400 | 2072 | 2257 | 1969 | 2174 |
| *p*-Coumaric acid | 0.0182 | 0.0178 | 0.0141 | 0.0136 | 0.0154 |
| Ferulic acid | 0.191 | 0.207 | 0.176 | 0.177 | 0.179 |
|  | | | | | |
| Mycotoxins^3^, ppm, (unless indicated otherwise) | | | | | |
| Deoxynivalenol (DON) | <0.05 | <0.05 | 0.05<0.16 | 0.05<0.16 | 0.212 |
| Zearalenone, ppb | 7.86 | <1 | 1<4 | 11.3 | 49.1 |
| Fumonisin B1 | 3.10 | 2.62 | 3.18 | 4.17 | 5.28 |
| Fumonisin B2 | 0.840 | 0.710 | 0.787 | 1.13 | 1.45 |
| Fumonisin B3 | 0.423 | 0.356 | 0.432 | 0.516 | 0.783 |
| Moniliformin | 0.06<0.2 | 0.226 | 0.405 | 2.55 | 1.45 |

^1^	The following fatty acids were “0.00” for all maize sources: caprylic (C8:0), capric (C10:0), lauric (C12:0), myristic (C14:0), myristoleic (C14:1), pentadecanoic (C15:0), pentadecenoic (C15:1), heptadecenoic (C17:1), heptadecadienoic (C17:2), gamma linolenic (C18:3), nonadecanoic (C19:0), eicosadienoic (C20:2), eicosatrienoic (C20:3), arachidonic (C20:4, heneicosanoic (C21:0), erucic (C22:1) and tricosanoic (C23:0).

^2^	Furfural was below the limit of detection for all maize sources.

^3^	The following mycotoxins were below the limit of detection or between the limit of detection and below the limit of quantification for all maize sources: aflatoxin B1, B2, G1 and G2, ochratoxin A, oosporein, ergosine, ergotamine, ergocronine, ergocryptine, ergocristine, T-2 toxin, 3/15-Acetyl-DON, and cyclopiazonic acid.

**^Table S2. Analyzed Nutrient and Contaminant Composition (as-is basis) of Diets Fed to Rodents^**

| **Item** | **Control** | **DP23211 High** | **DP23211 Low** | **P0928** | **P0993** | **P1105** |
| --- | --- | --- | --- | --- | --- | --- |
| Proximates, fibers, and carbohydrates, % (unless otherwise indicated) | | | | | | |
| Dry Matter | 87.8 | 88.0 | 87.9 | 87.0 | 87.3 | 87.3 |
| Crude Protein | 19.371 | 19.949 | 19.861 | 19.474 | 19.849 | 19.546 |
| Crude Fat | 6.47 | 6.90 | 7.61 | 4.98 | 5.99 | 5.50 |
| Acid Detergent Fiber | 6.12 | 6.58 | 6.37 | 5.43 | 5.69 | 5.51 |
| Neutral Detergent Fiber | 14.9 | 10.7 | 13.5 | 11.2 | 11.9 | 11.6 |
| Crude Fiber | 4.45 | 4.12 | 4.16 | 4.25 | 4.34 | 3.69 |
| Ash | 5.10 | 4.85 | 5.08 | 4.4 | 4.93 | 4.53 |
| Carbohydrates | 56.8 | 56.3 | 55.3 | 58.2 | 56.5 | 57.8 |
| Gross Energy, Kcal/100g | 403 | 400 | 402 | 399 | 398 | 399 |
|  | | | | | | |
| Amino acids, % | | | | | | |
| Alanine | 1.03 | 1.03 | 0.993 | 0.888 | 0.931 | 0.948 |
| Arginine | 1.21 | 1.21 | 1.21 | 1.11 | 1.18 | 1.22 |
| Aspartic Acid | 1.85 | 1.82 | 1.76 | 1.83 | 1.85 | 1.97 |
| Cystine | 0.415 | 0.381 | 0.382 | 0.315 | 0.346 | 0.311 |
| Glutamic Acid | 3.54 | 3.54 | 3.44 | 3.37 | 3.38 | 3.57 |
| Glycine | 1.02 | 1.04 | 0.979 | 0.886 | 0.947 | 0.952 |
| Histidine | 0.571 | 0.591 | 0.590 | 0.533 | 0.586 | 0.573 |
| Isoleucine | 0.823 | 0.860 | 0.832 | 0.785 | 0.827 | 0.828 |
| Leucine | 1.65 | 1.69 | 1.71 | 1.52 | 1.58 | 1.59 |
| Lysine | 1.18 | 1.16 | 1.10 | 1.06 | 1.10 | 1.18 |
| Methionine | 0.553 | 0.490 | 0.516 | 0.288 | 0.480 | 0.323 |
| Phenylalanine | 1.11 | 1.12 | 1.16 | 1.04 | 1.12 | 1.12 |
| Proline | 1.26 | 1.29 | 1.27 | 1.11 | 1.16 | 1.16 |
| Serine | 0.988 | 1.02 | 1.01 | 0.933 | 0.98 | 0.997 |
| Threonine | 0.833 | 0.850 | 0.827 | 0.771 | 0.796 | 0.812 |
| Tryptophan | 0.189 | 0.189 | 0.178 | 0.191 | 0.198 | 0.189 |
| Tyrosine | 0.554 | 0.571 | 0.576 | 0.430 | 0.555 | 0.559 |
| Valine | 1.03 | 1.05 | 1.03 | 0.968 | 0.998 | 1.02 |
|  | | | | | | |
| Minerals & heavy metals^1^, ppm | | | | | | |
| Calcium | 0.000980 | 0.00133 | 0.00369 | 0.000774 | 0.000843 | 0.00107 |
| Copper | 0.0192 | 0.0213 | 0.0153 | 0.0101 | 0.0117 | 0.0127 |
| Iron | 0.183 | 0.184 | 0.179 | 0.168 | 0.164 | 0.166 |
| Magnesium | 0.00927 | 0.00996 | 0.00953 | 0.00353 | 0.00456 | 0.00543 |
| Manganese | 0.723 | 0.817 | 0.740 | 0.504 | 0.561 | 0.608 |
| Phosphorus | 0.898 | 0.858 | 0.868 | 1.02 | 1.01 | 1.03 |
| Potassium | 0.259 | 0.275 | 0.261 | 0.349 | 0.376 | 0.364 |
| Sodium | 0.00948 | 0.0108 | 0.0106 | 0.00443 | 0.00632 | 0.00671 |
| Zinc | 0.323 | 0.353 | 0.343 | 0.476 | 0.375 | 0.316 |
| Selenium | 0.326 | 0.583 | 0.520 | 0.283 | 0.227 | 0.296 |
| Cadmium | 1.74 | 1.78 | 2.06 | 1.87 | 2.05 | 1.91 |
| Mercury | 0.223 | 0.367 | 0.279 | 0.184 | 0.176 | 0.222 |
| Cobalt | 0.143 | 0.266 | 0.191 | 0.115 | <0.100 | 0.126 |
| Lead | 7703 | 6994 | 5569 | 7308 | 6892 | 6306 |
| Chloride | 5.73 | 7.52 | 4.59 | 8.24 | 9.08 | 5.57 |
| Fluoride | 0.548 | 0.786 | 0.805 | 0.496 | 0.566 | 0.561 |
| Iodide | 0.000980 | 0.00133 | 0.00369 | 0.000774 | 0.000843 | 0.00107 |
|  | | | | | | |
| Vitamins^2^, mg/100g (unless otherwise indicated) | | | | | | |
| Thiamine | 1.02 | 0.870 | 0.722 | 0.391 | 0.559 | 0.578 |
| Riboflavin | 0.693 | 0.453 | 0.673 | 0.280 | 0.513 | 0.412 |
| Pyridoxine | 0.442 | 0.563 | 0.508 | 0.953 | 0.614 | 0.679 |
| Folic acid | 0.462 | 0.596 | 1.09 | 0.616 | 0.511 | 0.765 |
| Niacin | 2.24 | 4.03 | 2.25 | 2.17 | 2.33 | 2.28 |
| Pantothenic acid | 1.79 | 0.778 | 1.38 | 1.00 | 1.44 | 1.48 |
| Beta carotene, IU/100g | 59.8 | 70.8 | 54.8 | 65.3 | 77.3 | 46.7 |
| Total tocopherols | 5.77 | 5.97 | 6.11 | 4.27 | 4.80 | 6.07 |
| alpha tocopherol | 1.04 | 1.05 | 1.09 | 0.954 | 0.718 | 1.25 |
| beta tocopherol | 0.429 | 0.419 | 0.441 | 0.186 | 0.190 | 0.311 |
| gamma tocopherol | 3.19 | 3.31 | 3.39 | 2.20 | 2.98 | 3.26 |
| delta tocopherol | 1.11 | 1.19 | 1.19 | 0.932 | 0.908 | 1.25 |
| Vitamin A (as retinol), IU RE/100g | 674 | 112 | 313 | <72.0 | 355 | 2189 |
| Vitamin B12 | 0.0033 | 0.00407 | 0.00357 | 0.00214 | 0.00175 | 0.00172 |
| Biotin | 0.0813 | 0.0826 | 0.0660 | 0.0841 | 0.0842 | 0.0727 |
| Choline | 176 | 168 | 169 | 141 | 144 | 127 |

**^Table S2. Analyzed Nutrient and Contaminant Composition (as-is basis) of Diets Fed to Rodents (continued)^**

| **Item** | **Control** | **DP23211 High** | **DP23211 Low** | **P0928** | **P0993** | **P1105** |
| --- | --- | --- | --- | --- | --- | --- |
| Isoflavones^3^, mg/kg | | | | | | |
| Daidzin | 218 | 196 | 233 | 255 | 277 | 276 |
| Glycitin | 35.4 | 36.3 | 45.5 | 51 | 53.6 | 58.6 |
| Genistin | 230 | 199 | 266 | 309 | 353 | 324 |
| Daidzein | 7.72 | 6.0 | 7.4 | 12.8 | 16 | 13.6 |
| Genistein | 11.6 | 17.5 | 10.9 | 12.0 | 13.1 | 12.0 |
|  | | | | | | |
| Mycotoxins^4^, ppm (unless otherwise indicated) | | | | | | |
| Aflatoxin G1 (ppb) | <1 | 4.36 | <1 | <1 | <1 | <1 |
| Deoxynivalenol (DON) | 0.05<0.16 | <0.05 | <0.05 | 0.05<0.16 | <0.05 | 0.248 |
| Zearalenone (ppb) | 5.59 | 4.30 | 5.41 | 89.6 | 8.21 | 19.3 |
| Fumonisin B1 | 1.51 | 1.34 | 1.46 | 1.70 | 1.29 | 4.42 |
| Fumonisin B2 | 0.541 | 0.375 | 0.351 | 0.546 | 0.317 | 0.991 |
| Fumonisin B3 | 0.205 | 0.196 | 0.146 | 0.171 | 0.157 | 0.599 |
| Monoliformin | 0.06<0.2 | 0.06<0.2 | 0.210 | 0.447 | 0.06<0.2 | 0.553 |
|  | | | | | | |
| Pesticides^5^ and PCBs^6^, ppm | | | | | | |
| Malathion | 0.003<0.01 | 0.0352 | 0.0156 | 0.003<0.01 | 0.003<0.01 | 0.003<0.01 |

^1^	Heavy metals arsenic and chromium were below their respective limit of detection for all diets.

^2^	Vitamin D3 was below the limit of detection for all diets.

^3^	The isoflavone glycitein was below the limit of detection for all diets.

^4^	The following mycotoxins were below the limit of detection or between the limit of detection and below the limit of quantification for all diets: aflatoxin B1, B2, and G2, T-2 toxin, ochratoxin A, cyclopiazonic acid, 3/15-acetyl-DON, ergosine, ergotamine, ergocornine, ergocryptine, ergocristine, and oosporein.

^5^	Pesticides (alpha-BHC, beta-BHC, gamma-BHC, delta-BHC, HCB, diazinon, disulfoton, heptachlor, aldrin, heptachlor epoxide, Endosulfan I, Endosulfan II, Endosulfan Sulfate, endrin, 4,4’-DDE, dieldrin, 4,4’-DDD, 4,4’-DDT, carbophenothion (Trithion), disulfoton, ethion, ethyl parathion, methyl parathion, phorate, mirex, methoxychlor, cis-chlordane, and trans-chlordane) were below the limit of detection or between the limit of detection and below the limit of quantification for all diets.

^6^	All PCBs were the below the limits of detection for all diets.

**Table S3. Analyzed Nutrient Composition (as-is basis) of Diets Fed to Broilers**

| **Item** | **Control** | **DP23211** | **P0928** | **P0993** | **P1105** |
| --- | --- | --- | --- | --- | --- |
|  | ========================================Starter Phase==================================== | | | | |
| Proximates and minerals, % | | | | | |
| Moisture | 11.3 | 11.0 | 11.6 | 11.7 | 11.6 |
| Protein | 22.516 | 22.612 | 22.950 | 22.726 | 22.771 |
| Fat | 5.41 | 4.87 | 5.09 | 4.98 | 5.16 |
| Fiber | 2.21 | 2.12 | 2.02 | 2.00 | 2.05 |
| Ash | 5.19 | 5.42 | 5.04 | 5.04 | 5.23 |
| Calcium | 1.04 | 1.16 | 1.12 | 1.17 | 1.15 |
| Phosphorus | 0.754 | 0.827 | 0.772 | 0.773 | 0.803 |
| Gross energy, kcal/kg | 4140 | 4037 | 4090 | 4097 | 4068 |
| Essential amino acids, % | | | | | |
| Arginine | 1.38 | 1.34 | 1.48 | 1.44 | 1.49 |
| Histidine | 0.547 | 0.549 | 0.559 | 0.542 | 0.550 |
| Isoleucine | 0.971 | 0.981 | 1.01 | 0.994 | 1.02 |
| Leucine | 2.05 | 2.05 | 2.02 | 2.02 | 2.01 |
| Lysine | 1.26 | 1.35 | 1.27 | 1.20 | 1.24 |
| Methionine | 0.493 | 0.420 | 0.396 | 0.458 | 0.411 |
| Methionine + Cystine | 1.09 | 0.969 | 1.07 | 1.10 | 1.05 |
| Phenylalanine | 1.18 | 1.13 | 1.23 | 1.22 | 1.23 |
| Threonine | 0.980 | 0.982 | 1.03 | 1.00 | 1.02 |
| Tryptophan | 0.188 | 0.203 | 0.179 | 0.189 | 0.199 |
| Valine | 1.24 | 1.23 | 1.34 | 1.29 | 1.34 |
|  | | | | | |
|  | ========================================Grower Phase==================================== | | | | |
| Proximates and minerals, % | | | | | |
| Moisture | 11.5 | 11.3 | 11.8 | 11.5 | 11.3 |
| Protein | 20.626 | 20.846 | 20.822 | 20.767 | 20.691 |
| Fat | 6.09 | 4.96 | 5.44 | 5.74 | 5.52 |
| Fiber | 2.16 | 2.18 | 2.06 | 1.97 | 2.07 |
| Ash | 4.65 | 4.57 | 4.33 | 4.47 | 4.64 |
| Calcium | 0.889 | 0.963 | 0.952 | 0.913 | 1.00 |
| Phosphorus | 0.698 | 0.678 | 0.717 | 0.694 | 0.701 |
| Gross energy, kcal/kg | 4105 | 4097 | 4081 | 4191 | 4106 |
| Essential amino acids, % | | | | | |
| Arginine | 1.30 | 1.34 | 1.28 | 1.26 | 1.29 |
| Histidine | 0.537 | 0.583 | 0.504 | 0.503 | 0.502 |
| Isoleucine | 0.896 | 0.930 | 0.894 | 0.901 | 0.889 |
| Leucine | 1.94 | 2.00 | 1.83 | 1.86 | 1.79 |
| Lysine | 1.19 | 1.18 | 1.18 | 1.17 | 1.19 |
| Methionine | 0.419 | 0.405 | 0.356 | 0.359 | 0.368 |
| Methionine + Cystine | 0.957 | 0.886 | 0.943 | 0.924 | 0.937 |
| Phenylalanine | 1.13 | 1.17 | 1.06 | 1.09 | 1.07 |
| Threonine | 0.907 | 0.930 | 0.906 | 0.906 | 0.913 |
| Tryptophan | 0.178 | 0.187 | 0.181 | 0.185 | 0.194 |
| Valine | 1.13 | 1.14 | 1.18 | 1.16 | 1.16 |
|  | | | | | |
|  | =======================================Finisher Phase==================================== | | | | |
| Proximates and minerals, % | | | | | |
| Moisture | 11.3 | 11.1 | 11.5 | 11.1 | 11.4 |
| Protein | 19.312 | 19.424 | 19.396 | 19.314 | 18.984 |
| Fat | 8.44 | 7.32 | 7.83 | 7.65 | 7.76 |
| Fiber | 2.24 | 2.27 | 2.15 | 2.06 | 2.02 |
| Ash | 4.67 | 4.55 | 4.63 | 4.59 | 4.64 |
| Calcium | 0.900 | 0.884 | 0.975 | 0.954 | 0.960 |
| Phosphorus | 0.738 | 0.695 | 0.767 | 0.725 | 0.713 |
| Gross energy, kcal/kg | 4222 | 4168 | 4181 | 4197 | 4190 |
| Essential amino acids, % | | | | | |
| Arginine | 1.23 | 1.21 | 1.24 | 1.30 | 1.24 |
| Histidine | 0.580 | 0.575 | 0.546 | 0.572 | 0.512 |
| Isoleucine | 0.833 | 0.835 | 0.851 | 0.857 | 0.841 |
| Leucine | 1.78 | 1.81 | 1.72 | 1.76 | 1.69 |
| Lysine | 1.01 | 1.04 | 1.03 | 0.940 | 0.984 |
| Methionine | 0.448 | 0.425 | 0.376 | 0.382 | 0.382 |
| Methionine + Cystine | 0.858 | 0.812 | 0.840 | 0.809 | 0.815 |
| Phenylalanine | 1.04 | 1.04 | 1.02 | 1.12 | 1.02 |
| Threonine | 0.832 | 0.834 | 0.857 | 0.863 | 0.837 |
| Tryptophan | 0.181 | 0.200 | 0.179 | 0.180 | 0.182 |
| Valine | 0.963 | 0.939 | 1.04 | 1.03 | 1.01 |

**Table S4. Body Weight and Body Weight Gain Summary of Rats fed Maize Source Diets**

|  | **Gender** | **Statistical Parameter** | **Control** | **DP23211**  **High** | **DP23211**  **Low** | **P0928** | **P0993** | **P1105** |
| --- | --- | --- | --- | --- | --- | --- | --- | --- |
| **Body Weight (BW) (g)** | | | | | | | | |
| **BW:**  **DAY 1** | Female | Mean | 170.8 | 170.0 | 170.1 | 172.4 | 171.7 | 174.5 |
|  |  | 95% CI | 161.7 - 179.9 | 160.9 - 179.1 | 161.0 - 179.2 | -- | -- | -- |
|  |  | Range | 153.5 - 187.1 | 150.7 - 189.3 | 152.5 - 193.0 | 154.2 - 192.3 | 142.8 - 192.0 | 157.3 - 194.4 |
|  | Male | Mean | 250.1 | 249.5 | 249.4 | 248.4 | 250.2 | 250.2 |
|  |  | 95% CI | 241.0 - 259.2 | 240.4 - 258.6 | 240.3 - 258.4 | -- | -- | -- |
|  |  | Range | 228.1 - 263.9 | 222.6 - 266.4 | 235.9 - 264.4 | 227.8 - 262.7 | 229.9 - 272.1 | 237.7 - 262.9 |
| **BW:**  **DAY 8** | Female | Mean | 196.2 | 190.7 | 194.1 | 192.8^a^ | 196.9 | 195.5 |
|  |  | 95% CI | 184.6 - 207.9 | 179.1 - 202.4 | 182.4 - 205.7 | -- | -- | -- |
|  |  | Range | 166.8 - 223.3 | 166.2 - 210.6 | 168.4 - 229.5 | 173.8 - 212.2 | 173.4 - 213.3 | 176.3 - 234.3 |
|  | Male | Mean | 310.5 | 313.0 | 312.6 | 309.3 | 312.9 | 312.0 |
|  |  | 95% CI | 298.8 - 322.1 | 301.4 - 324.7 | 301.0 - 324.3 | -- | -- | -- |
|  |  | Range | 276.6 - 332.1 | 286.2 - 337.5 | 293.6 - 338.5 | 278.3 - 337.1 | 286.1 - 343.8 | 290.3 - 324.4 |
| **BW:**  **DAY 15** | Female | Mean | 222.7 | 212.8** | 216.7** | 219.6^a^ | 220.5 | 220.6 |
|  |  | 95% CI | 209.4 - 236.0 | 199.5 - 226.1 | 203.4 - 230.0 | -- | -- | -- |
|  |  | Range | 199.9 - 255.4 | 182.3 - 244.8 | 177.3 - 248.8 | 203.2 - 243.7 | 192.6 - 248.7 | 199.4 - 262.3 |
|  | Male | Mean | 368.4 | 369.0 | 370.8 | 361.5 | 369.3 | 365.2 |
|  |  | 95% CI | 353.6 - 383.1 | 354.2 - 383.8 | 356.0 - 385.6 | -- | -- | -- |
|  |  | Range | 324.2 - 414.5 | 330.5 - 392.8 | 346.1 - 416.1 | 322.2 - 395.5 | 333.5 - 409.3 | 342.3 - 383.8 |
| **BW:**  **DAY 22** | Female | Mean | 239.6 | 227.1** | 237.4 | 234.7^a^ | 237.8 | 239.1 |
|  |  | 95% CI | 223.5 - 255.8 | 210.9 - 243.2 | 221.2 - 253.5 | -- | -- | -- |
|  |  | Range | 211.3 - 276.7 | 193.0 - 256.4 | 191.5 - 282.8 | 201.6 - 261.1 | 205.0 - 268.6 | 206.3 - 284.4 |
|  | Male | Mean | 410.1 | 411.7 | 414.3 | 402.1 | 409.4 | 405.3 |
|  |  | 95% CI | 392.7 - 427.6 | 394.3 - 429.2 | 396.9 - 431.8 | -- | -- | -- |
|  |  | Range | 355.9 - 469.5 | 370.9 - 438.0 | 384.7 - 473.6 | 364.7 - 453.5 | 365.0 - 459.9 | 376.2 - 431.6 |
| **BW:**  **DAY 29** | Female | Mean | 251.3 | 238.3** | 248.5 | 248.2^a^ | 249.6 | 252.2 |
|  |  | 95% CI | 234.0 - 268.7 | 221.0 - 255.6 | 231.2 - 265.9 | -- | -- | -- |
|  |  | Range | 222.1 - 293.0 | 197.3 - 274.3 | 205.0 - 293.6 | 217.5 - 275.1 | 209.4 - 284.6 | 214.6 - 307.0 |
|  | Male | Mean | 448.6 | 450.8 | 453.4 | 436.3 | 446.1 | 441.8 |
|  |  | 95% CI | 428.4 - 468.8 | 430.6 - 471.0 | 433.2 - 473.6 | -- | -- | -- |
|  |  | Range | 383.1 - 515.7 | 405.4 - 485.8 | 413.7 - 535.3 | 396.7 - 484.6 | 399.0 - 503.4 | 405.2 - 474.3 |
| **BW:**  **DAY 36** | Female | Mean | 264.1 | 250.8** | 261.6 | 255.7^a^ | 258.1 | 262.1 |
|  |  | 95% CI | 246.1 - 282.0 | 232.9 - 268.8 | 243.7 - 279.6 | -- | -- | -- |
|  |  | Range | 220.7 - 311.4 | 211.2 - 288.7 | 217.6 - 321.6 | 227.5 - 288.0 | 221.3 - 280.6 | 206.2 - 314.7 |
|  | Male | Mean | 479.7 | 479.7 | 483.3 | 461.8 | 473.9 | 470.0 |
|  |  | 95% CI | 457.9 - 501.4 | 458.0 - 501.5 | 461.5 - 505.0 | -- | -- | -- |
|  |  | Range | 408.6 - 562.3 | 423.8 - 517.4 | 439.3 - 584.4 | 416.6 - 515.6 | 414.8 - 533.8 | 432.6 - 503.2 |
| **BW:**  **DAY 43** | Female | Mean | 277.3 | 262.5** | 271.1 | 268.7^a^ | 270.5 | 274.2 |
|  |  | 95% CI | 258.9 - 295.8 | 244.0 - 281.0 | 252.6 - 289.6 | -- | -- | -- |
|  |  | Range | 238.1 - 328.9 | 219.7 - 299.9 | 216.5 - 332.4 | 243.3 - 294.4 | 229.6 - 314.5 | 222.8 - 333.8 |
|  | Male | Mean | 507.2 | 505.4 | 511.6 | 486.2 | 496.7 | 494.4 |
|  |  | 95% CI | 483.7 - 530.8 | 481.8 - 528.9 | 488.0 - 535.1 | -- | -- | -- |
|  |  | Range | 433.2 - 600.8 | 440.5 - 545.2 | 460.5 - 624.5 | 434.6 - 544.2 | 426.1 - 550.4 | 451.9 - 531.9 |
| **BW:**  **DAY 51** | Female | Mean | 287.7 | 270.7** | 282.4 | 277.9^a^ | 277.9 | 283.4 |
|  |  | 95% CI | 268.7 - 306.6 | 251.7 - 289.6 | 263.5 - 301.4 | -- | -- | -- |
|  |  | Range | 247.1 - 342.0 | 234.9 - 307.8 | 226.1 - 338.3 | 249.5 - 309.4 | 234.0 - 324.0 | 227.9 - 343.4 |
|  | Male | Mean | 534.9 | 529.1 | 538.3 | 511.3 | 524.1 | 519.2 |
|  |  | 95% CI | 510.4 - 559.4 | 504.5 - 553.6 | 513.8 - 562.8 | -- | -- | -- |
|  |  | Range | 455.6 - 639.2 | 459.1 - 578.4 | 480.5 - 661.2 | 458.4 - 583.3 | 457.6 - 582.4 | 469.2 - 562.1 |
| **BW:**  **DAY 57** | Female | Mean | 288.6 | 275.2** | 288.9 | 283.3^a^ | 283.7 | 291.2 |
|  |  | 95% CI | 269.5 - 307.8 | 256.0 - 294.4 | 269.8 - 308.1 | -- | -- | -- |
|  |  | Range | 249.8 - 335.9 | 236.0 - 305.0 | 238.3 - 351.2 | 245.1 - 327.8 | 236.2 - 330.8 | 234.3 - 353.8 |
|  | Male | Mean | 555.1 | 545.6 | 559.0 | 526.9 | 542.2 | 539.5 |
|  |  | 95% CI | 527.9 - 582.3 | 518.4 - 572.8 | 531.8 - 586.2 | -- | -- | -- |
|  |  | Range | 470.3 - 666.1 | 476.3 - 597.9 | 498.8 - 695.8 | 470.4 - 607.8 | 470.8 - 595.6 | 491.7 - 586.2 |
| **BW:**  **DAY 64** | Female | Mean | 300.3 | 282.7** | 296.1 | 290.9^a^ | 290.1 | 298.0 |
|  |  | 95% CI | 281.1 - 319.5 | 263.5 - 301.8 | 277.0 - 315.3 | -- | -- | -- |
|  |  | Range | 255.6 - 358.7 | 246.8 - 325.6 | 245.5 - 365.0 | 262.3 - 328.2 | 249.3 - 326.8 | 249.3 - 354.9 |
|  | Male | Mean | 573.6 | 561.5 | 577.7 | 544.3 | 556.8 | 555.9 |
|  |  | 95% CI | 547.1 - 600.2 | 534.9 - 588.0 | 551.2 - 604.2 | -- | -- | -- |
|  |  | Range | 488.5 - 680.6 | 483.6 - 618.3 | 519.2 - 711.3 | 489.7 - 635.3 | 485.6 - 619.2 | 476.0 - 606.1 |

**Table S4. Body Weight and Body Weight Gain Summary of Rats fed Maize Source Diets (continued)**

|  | **Gender** | **Statistical Parameter** | **Control** | **DP23211**  **High** | **DP23211**  **Low** | **P0928** | **P0993** | **P1105** |
| --- | --- | --- | --- | --- | --- | --- | --- | --- |
| **Body Weight (BW) (g) (continued)** | | | | | | | | |
| **BW:**  **DAY 71** | Female | Mean | 306.5 | 291.7** | 302.7 | 296.7^a^ | 297.8 | 302.6 |
|  |  | 95% CI | 286.6 - 326.3 | 271.9 - 311.5 | 282.8 - 322.5 | -- | -- | -- |
|  |  | Range | 270.4 - 362.7 | 254.6 - 334.5 | 244.3 - 356.3 | 269.0 - 335.7 | 259.8 - 346.1 | 252.3 - 367.4 |
|  | Male | Mean | 585.2 | 573.8 | 592.0 | 557.0 | 567.7 | 569.0 |
|  |  | 95% CI | 557.2 - 613.2 | 545.8 - 601.8 | 564.0 - 620.0 | -- | -- | -- |
|  |  | Range | 491.5 - 696.7 | 487.5 - 623.1 | 516.4 - 744.2 | 495.6 - 646.6 | 493.7 - 631.5 | 478.0 - 624.1 |
| **BW:**  **DAY 78** | Female | Mean | 311.6 | 295.0** | 306.9 | 303.2^a^ | 304.3 | 308.8 |
|  |  | 95% CI | 291.1 - 332.1 | 274.5 - 315.5 | 286.4 - 327.4 | -- | -- | -- |
|  |  | Range | 273.8 - 365.5 | 255.8 - 337.4 | 256.1 - 358.4 | 269.3 - 340.5 | 251.9 - 356.4 | 260.0 - 378.4 |
|  | Male | Mean | 600.3 | 585.8 | 609.4 | 572.9 | 585.9 | 585.2 |
|  |  | 95% CI | 571.6 - 628.9 | 557.1 - 614.5 | 580.8 - 638.1 | -- | -- | -- |
|  |  | Range | 512.1 - 719.0 | 498.4 - 639.6 | 532.8 - 764.7 | 507.6 - 667.0 | 506.6 - 651.5 | 491.0 - 651.3 |
| **BW:**  **DAY 85** | Female | Mean | 306.6 | 293.3 | 303.3 | 300.2^a^ | 300.1 | 309.3 |
|  |  | 95% CI | 286.1 - 327.2 | 272.7 - 313.8 | 282.7 - 323.8 | -- | -- | -- |
|  |  | Range | 272.7 - 349.5 | 251.7 - 330.3 | 251.4 - 364.0 | 260.1 - 338.2 | 244.7 - 349.5 | 256.0 - 383.7 |
|  | Male | Mean | 608.5 | 594.3 | 616.2 | 578.5 | 592.1 | 588.9 |
|  |  | 95% CI | 578.3 - 638.7 | 564.1 - 624.5 | 586.0 - 646.4 | -- | -- | -- |
|  |  | Range | 511.9 - 741.2 | 507.3 - 654.6 | 536.4 - 772.7 | 515.1 - 684.0 | 505.8 - 661.5 | 475.5 - 650.9 |
| **BW:**  **DAY 91** | Female | Mean | 313.2 | 298.2** | 309.3 | 302.3^a^ | 303.5 | 309.4 |
|  |  | 95% CI | 294.4 - 332.0 | 279.4 - 317.0 | 290.5 - 328.1 | -- | -- | -- |
|  |  | Range | 276.6 - 362.9 | 266.7 - 336.4 | 254.0 - 357.1 | 269.1 - 333.3 | 255.6 - 354.7 | 265.9 - 373.0 |
|  | Male | Mean | 613.9 | 601.4 | 624.1 | 584.2 | 597.9 | 592.8 |
|  |  | 95% CI | 583.9 - 643.9 | 571.4 - 631.4 | 594.1 - 654.1 | -- | -- | -- |
|  |  | Range | 515.4 - 751.5 | 519.5 - 664.9 | 544.2 - 781.7 | 514.4 - 687.1 | 511.5 - 670.9 | 467.9 - 657.4 |
|  | **Body Weight Gain (BWG) (g)** | | | | | | | |
| **BWG:**  **DAY 1 -**  **DAY 8** | Female | Mean | 25.4 | 20.7 | 23.9 | 20.4 | 25.1 | 21.0 |
|  |  | 95% CI | 21.0 - 29.9 | 16.2 - 25.2 | 19.5 - 28.4 | -- | -- | -- |
|  |  | Range | 6.6 - 40.3 | 4.7 - 35.7 | 8.2 - 39.4 | 9.5 - 33.8 | 18.9 - 33.6 | 1.7 - 43.4 |
|  | Male | Mean | 60.4 | 63.6 | 63.3 | 60.9 | 62.6 | 61.8 |
|  |  | 95% CI | 55.9 - 64.9 | 59.1 - 68.1 | 58.8 - 67.8 | -- | -- | -- |
|  |  | Range | 43.3 - 75.6 | 46.3 - 75.2 | 52.5 - 74.1 | 50.1 - 76.2 | 51.6 - 71.7 | 52.6 - 71.6 |
| **BWG:**  **DAY 8 -**  **DAY 15** | Female | Mean | 26.5 | 22.1 | 22.6 | 26.1^a^ | 23.7 | 25.1 |
|  |  | 95% CI | 22.5 - 30.4 | 18.1 - 26.0 | 18.7 - 26.5 | -- | -- | -- |
|  |  | Range | 20.3 - 36.5 | 14.2 - 35.4 | 8.9 - 34.4 | 13.2 - 42.7 | 13.1 - 35.4 | 9.2 - 38.4 |
|  | Male | Mean | 57.9 | 56.0 | 58.2 | 52.3 | 56.4 | 53.2 |
|  |  | 95% CI | 54.0 - 61.8 | 52.0 - 59.9 | 54.2 - 62.1 | -- | -- | -- |
|  |  | Range | 47.6 - 82.4 | 43.8 - 73.6 | 49.2 - 77.6 | 39.7 - 70.6 | 38.9 - 66.6 | 41.6 - 62.3 |
| **BWG:**  **DAY 15 -**  **DAY 22** | Female | Mean | 16.9 | 14.3 | 20.7 | 15.2^a^ | 17.2 | 18.5 |
|  |  | 95% CI | 12.1 - 21.7 | 9.5 - 19.1 | 15.9 - 25.5 | -- | -- | -- |
|  |  | Range | 7.4 - 27.0 | 2.5 - 23.3 | 8.2 - 37.2 | -1.6 - 33.0 | 8.0 - 29.0 | 6.4 - 32.2 |
|  | Male | Mean | 41.8 | 42.8 | 43.5 | 40.5 | 40.1 | 40.1 |
|  |  | 95% CI | 37.6 - 45.9 | 38.6 - 46.9 | 39.4 - 47.7 | -- | -- | -- |
|  |  | Range | 15.1 - 55.3 | 34.2 - 50.7 | 32.8 - 57.5 | 25.9 - 63.1 | 31.3 - 50.6 | 31.3 - 49.4 |
| **BWG:**  **DAY 22 -**  **DAY 29** | Female | Mean | 11.7 | 11.2 | 11.2 | 13.5^a^ | 11.8 | 13.1 |
|  |  | 95% CI | 8.0 - 15.5 | 7.5 - 15.0 | 7.4 - 14.9 | -- | -- | -- |
|  |  | Range | 2.7 - 17.3 | 2.8 - 27.0 | -3.0 - 18.1 | -5.0 - 24.2 | 4.4 - 18.3 | -5.5 - 25.1 |
|  | Male | Mean | 38.4 | 39.0 | 39.1 | 34.3 | 36.7 | 36.4 |
|  |  | 95% CI | 34.7 - 42.2 | 35.3 - 42.8 | 35.3 - 42.8 | -- | -- | -- |
|  |  | Range | 27.2 - 54.9 | 29.6 - 53.4 | 25.6 - 61.7 | 23.6 - 47.2 | 22.5 - 45.6 | 21.9 - 52.6 |
| **BWG:**  **DAY 29 -**  **DAY 36** | Female | Mean | 12.7 | 12.5 | 13.1 | 7.5^a^ | 8.6 | 9.9 |
|  |  | 95% CI | 9.1 - 16.4 | 8.9 - 16.2 | 9.5 - 16.8 | -- | -- | -- |
|  |  | Range | -6.1 - 24.5 | -3.5 - 27.0 | -3.3 - 39.7 | -2.1 - 23.4 | -4.0 - 19.4 | -8.4 - 30.1 |
|  | Male | Mean | 31.1 | 29.0 | 29.9 | 25.5 | 27.8 | 28.3 |
|  |  | 95% CI | 27.4 - 34.7 | 25.3 - 32.6 | 26.2 - 33.5 | -- | -- | -- |
|  |  | Range | 19.8 - 46.6 | 17.5 - 39.7 | 15.7 - 49.1 | 17.0 - 44.5 | 14.4 - 37.4 | 11.2 - 39.6 |
| **BWG:**  **DAY 36 -**  **DAY 43** | Female | Mean | 13.3 | 11.7 | 9.5 | 13.0^a^ | 12.4 | 12.1 |
|  |  | 95% CI | 10.2 - 16.3 | 8.6 - 14.7 | 6.4 - 12.6 | -- | -- | -- |
|  |  | Range | 5.9 - 22.1 | -2.6 - 22.1 | -15.4 - 22.1 | 1.4 - 24.1 | -5.4 - 33.9 | -5.2 - 21.8 |
|  | Male | Mean | 27.6 | 25.6 | 28.3 | 24.4 | 22.7 | 24.3 |
|  |  | 95% CI | 24.5 - 30.7 | 22.5 - 28.7 | 25.2 - 31.4 | -- | -- | -- |
|  |  | Range | 16.6 - 38.5 | 16.7 - 36.0 | 18.5 - 40.1 | 12.7 - 40.4 | 11.3 - 35.2 | 15.9 - 36.1 |

**Table S4. Body Weight and Body Weight Gain Summary of Rats fed Maize Source Diets (continued)**

|  | **Gender** | **Statistical Parameter** | **Control** | **DP23211**  **High** | **DP23211**  **Low** | **P0928** | **P0993** | **P1105** |
| --- | --- | --- | --- | --- | --- | --- | --- | --- |
|  | **Body Weight Gain (BWG) (g) (continued)** | | | | | | | |
| **BWG:**  **DAY 43 - DAY 51** | Female | Mean | 10.3 | 8.2 | 11.3 | 9.2^a^ | 7.4 | 9.2 |
|  |  | 95% CI | 6.9 - 13.8 | 4.7 - 11.6 | 7.9 - 14.8 | -- | -- | -- |
|  |  | Range | 3.8 - 19.7 | -0.6 - 19.7 | 1.0 - 35.8 | -1.0 - 15.0 | 0.9 - 15.6 | 2.7 - 18.2 |
|  | Male | Mean | 27.7 | 23.7 | 26.7 | 25.1 | 27.4 | 24.9 |
|  |  | 95% CI | 24.2 - 31.1 | 20.2 - 27.1 | 23.3 - 30.1 | -- | -- | -- |
|  |  | Range | 18.1 - 41.6 | 16.3 - 39.5 | 16.6 - 40.4 | 10.1 - 39.1 | 13.0 - 39.1 | 10.2 - 39.1 |
| **BWG:**  **DAY 51 -**  **DAY 57** | Female | Mean | 1.0 | 4.5 | 6.5 | 5.4^a^ | 5.7 | 7.8 |
|  |  | 95% CI | -3.6 - 5.6 | -0.0 - 9.1 | 1.9 - 11.1 | -- | -- | -- |
|  |  | Range | -8.5 - 18.8 | -5.2 - 17.5 | -8.0 - 25.3 | -11.0 - 27.1 | -11.8 - 21.9 | -15.9 - 28.0 |
|  | Male | Mean | 20.3 | 16.6 | 20.8 | 15.6 | 18.2 | 20.3 |
|  |  | 95% CI | 16.7 - 23.8 | 13.0 - 20.1 | 17.2 - 24.3 | -- | -- | -- |
|  |  | Range | 2.6 - 26.9 | 3.3 - 24.8 | 5.2 - 34.6 | 8.7 - 27.6 | 13.2 - 25.8 | 5.6 - 31.6 |
| **BWG:**  **DAY 57 -**  **DAY 64** | Female | Mean | 11.7 | 7.5! | 7.2 | 7.6^a^ | 6.4 | 6.8 |
|  |  | 95% CI | 8.4 - 14.9 | 4.2 - 10.7 | 4.0 - 10.4 | -- | -- | -- |
|  |  | Range | -3.7 - 24.6 | -6.6 - 21.6 | -0.3 - 16.0 | -6.6 - 31.8 | -7.9 - 25.8 | -6.2 - 26.6 |
|  | Male | Mean | 18.5 | 15.8! | 18.7 | 17.4 | 14.6 | 16.4 |
|  |  | 95% CI | 15.3 - 21.7 | 12.6 - 19.1 | 15.4 - 21.9 | -- | -- | -- |
|  |  | Range | 10.2 - 28.8 | 7.3 - 27.5 | 9.0 - 32.6 | -2.2 - 27.5 | 3.2 - 29.5 | -15.7 - 29.3 |
| **BWG:**  **DAY 64 -**  **DAY 71** | Female | Mean | 6.2 | 9.0 | 6.5 | 5.8^a^ | 7.8 | 4.7 |
|  |  | 95% CI | 2.5 - 9.8 | 5.4 - 12.7 | 2.9 - 10.2 | -- | -- | -- |
|  |  | Range | -6.1 - 19.0 | 1.3 - 15.8 | -9.2 - 24.2 | -6.0 - 13.2 | -0.2 - 19.3 | -11.8 - 16.2 |
|  | Male | Mean | 11.5 | 12.3 | 14.3 | 12.7 | 10.8 | 13.0 |
|  |  | 95% CI | 7.9 - 15.2 | 8.7 - 16.0 | 10.6 - 17.9 | -- | -- | -- |
|  |  | Range | -5.3 - 17.3 | 3.9 - 20.0 | -4.2 - 32.9 | 4.6 - 26.5 | 5.3 - 18.1 | 2.0 - 19.7 |
| **BWG:**  **DAY 71 -**  **DAY 78** | Female | Mean | 5.2 | 3.3 | 4.2 | 6.5^a^ | 6.5 | 6.2 |
|  |  | 95% CI | 1.4 - 9.0 | -0.5 - 7.1 | 0.4 - 8.0 | -- | -- | -- |
|  |  | Range | -13.0 - 25.2 | -9.8 - 12.9 | -10.1 - 16.7 | -5.6 - 14.4 | -7.9 - 13.6 | -13.4 - 18.6 |
|  | Male | Mean | 15.1 | 12.0 | 17.5 | 15.9 | 18.3 | 16.3 |
|  |  | 95% CI | 11.3 - 18.9 | 8.2 - 15.8 | 13.7 - 21.3 | -- | -- | -- |
|  |  | Range | 5.5 - 23.1 | 3.7 - 21.7 | -2.4 - 35.1 | 9.5 - 20.4 | 12.3 - 25.8 | 2.3 - 27.2 |
| **BWG:**  **DAY 78 -**  **DAY 85** | Female | Mean | -5.0 | -1.8 | -3.6 | -3.0^a^ | -4.2 | 0.5 |
|  |  | 95% CI | -9.1 - -0.9 | -5.9 - 2.4 | -7.7 - 0.5 | -- | -- | -- |
|  |  | Range | -27.6 - 15.9 | -10.8 - 18.9 | -22.3 - 10.4 | -25.1 - 9.1 | -18.0 - 3.1 | -12.2 - 15.5 |
|  | Male | Mean | 8.2 | 8.5 | 6.8 | 5.6 | 6.2 | 3.7 |
|  |  | 95% CI | 4.2 - 12.3 | 4.4 - 12.6 | 2.7 - 10.9 | -- | -- | -- |
|  |  | Range | -11.6 - 22.2 | -5.3 - 16.9 | -7.0 - 20.2 | -9.7 - 21.6 | -4.1 - 21.2 | -15.5 - 13.1 |
| **BWG:**  **DAY 85 -**  **DAY 91** | Female | Mean | 6.6 | 4.9 | 6.0 | 2.2^a^ | 3.3 | 0.0 |
|  |  | 95% CI | 2.5 - 10.7 | 0.8 - 9.0 | 1.9 - 10.1 | -- | -- | -- |
|  |  | Range | -7.2 - 18.0 | -10.4 - 15.8 | -9.7 - 25.7 | -20.1 - 27.1 | -9.8 - 24.4 | -16.2 - 21.0 |
|  | Male | Mean | 5.4 | 7.1 | 7.9 | 5.8 | 5.8 | 4.0 |
|  |  | 95% CI | 1.3 - 9.5 | 3.0 - 11.2 | 3.8 - 12.0 | -- | -- | -- |
|  |  | Range | -19.7 - 20.5 | -9.7 - 18.3 | -3.1 - 19.8 | -5.0 - 24.0 | -4.8 - 20.5 | -10.8 - 18.8 |
| **BWG:**  **DAY 1 -**  **DAY 91** | Female | Mean | 142.4 | 128.2** | 139.1 | 128.7^a^ | 131.7 | 134.9 |
|  |  | 95% CI | 131.2 - 153.7 | 116.9 - 139.5 | 127.9 - 150.4 | -- | -- | -- |
|  |  | Range | 116.2 - 179.9 | 106.5 - 156.9 | 101.5 - 180.4 | 108.3 - 162.7 | 112.0 - 165.8 | 87.9 - 182.1 |
|  | Male | Mean | 363.9 | 351.9 | 374.8 | 335.8 | 347.7 | 342.6 |
|  |  | 95% CI | 337.4 - 390.3 | 325.4 - 378.4 | 348.3 - 401.2 | -- | -- | -- |
|  |  | Range | 263.0 - 487.6 | 279.6 - 414.7 | 291.0 - 517.3 | 259.9 - 435.8 | 281.6 - 402.3 | 226.4 - 400.7 |

Note: Test and control means are the LS-Means from the linear mixed model. Reference means are the arithmetic means. When a reported statistic was estimated to be < 10^-10^ in absolute value, the value was set to zero.

^a^	n=15, otherwise n=16

**	Statistically significant difference when compared to control at the 0.05 level; FDR not significantly different

!	Combined statistically significant difference when compared to combined control at the 0.05 level; combined FDR not significantly different

**Table S5. Food Consumption and Food Efficiency Summary of Rats fed Maize Source Diets**

|  | **Gender** | **Statistical Parameter** | **Control** | **DP23211**  **High** | **DP23211**  **Low** | **P0928** | **P0993** | **P1105** |
| --- | --- | --- | --- | --- | --- | --- | --- | --- |
|  | **Food Consumption (FC) (g/day)** | | | | | | | |
| **FC:**  **DAY 1 -**  **DAY 8** | Female | Mean | 17.1 | 17.9 | 17.3 | 17.2 | 17.4 | 18.4 |
|  |  | 95% CI | 16.0 - 18.3 | 16.8 - 19.1 | 16.1 - 18.4 | -- | -- | -- |
|  |  | Range | 15.1 - 19.3 | 15.1 - 20.8 | 16.2 - 19.5 | 14.4 - 19.5 | 15.0 - 19.7 | 16.7 - 19.5 |
|  | Male | Mean | 25.1 | 25.5 | 25.3 | 25.0 | 25.1 | 25.2 |
|  |  | 95% CI | 23.9 - 26.2 | 24.4 - 26.7 | 24.2 - 26.5 | -- | -- | -- |
|  |  | Range | 21.5 - 27.8 | 23.1 - 27.1 | 22.9 - 27.5 | 22.9 - 29.3 | 23.3 - 27.1 | 22.7 - 26.9 |
| **FC:**  **DAY 8 -**  **DAY 15** | Female | Mean | 18.4 | 19.1 | 19.0 | 18.3^a^ | 18.7 | 20.9 |
|  |  | 95% CI | 17.2 - 19.5 | 17.9 - 20.2 | 17.8 - 20.1 | -- | -- | -- |
|  |  | Range | 16.6 - 20.6 | 16.6 - 21.3 | 16.6 - 23.7 | 16.1 - 20.1 | 15.4 - 20.6 | 17.8 - 26.7 |
|  | Male | Mean | 26.7 | 27.6 | 27.0 | 26.6 | 27.0 | 26.0 |
|  |  | 95% CI | 25.5 - 27.8 | 26.4 - 28.7 | 25.8 - 28.1 | -- | -- | -- |
|  |  | Range | 24.4 - 29.5 | 26.2 - 28.9 | 25.1 - 29.9 | 24.9 - 30.5 | 24.1 - 29.1 | 24.0 - 27.1 |
| **FC:**  **DAY 15 -**  **DAY 22** | Female | Mean | 20.0 | 21.0 | 20.6 | 20.9^a^ | 19.0 | 21.3 |
|  |  | 95% CI | 17.6 - 22.3 | 18.6 - 23.3 | 18.2 - 23.0 | -- | -- | -- |
|  |  | Range | 16.8 - 23.7 | 15.8 - 25.1 | 16.2 - 26.5 | 17.8 - 24.4 | 15.2 - 22.3 | 17.7 - 25.7 |
|  | Male | Mean | 27.3 | 28.2 | 27.7 | 27.5 | 27.2 | 26.6 |
|  |  | 95% CI | 25.8 - 28.9 | 26.7 - 29.7 | 26.1 - 29.2 | -- | -- | -- |
|  |  | Range | 24.1 - 31.2 | 24.5 - 30.7 | 25.0 - 31.2 | 24.7 - 34.8 | 24.6 - 29.5 | 24.9 - 29.3 |
| **FC:**  **DAY 22 -**  **DAY 29** | Female | Mean | 18.8 | 19.5 | 19.2 | 19.2^a^ | 17.7 | 20.4 |
|  |  | 95% CI | 17.0 - 20.7 | 17.6 - 21.4 | 17.3 - 21.1 | -- | -- | -- |
|  |  | Range | 16.6 - 20.6 | 15.7 - 24.5 | 16.0 - 24.7 | 14.2 - 25.0 | 15.0 - 18.8 | 17.3 - 24.9 |
|  | Male | Mean | 28.5 | 29.0 | 29.3 | 28.9 | 27.7 | 26.8 |
|  |  | 95% CI | 26.6 - 30.4 | 27.1 - 30.9 | 27.4 - 31.1 | -- | -- | -- |
|  |  | Range | 26.3 - 34.4 | 26.5 - 31.8 | 24.6 - 33.0 | 24.1 - 42.1 | 24.7 - 29.4 | 24.0 - 28.7 |
| **FC:**  **DAY 29 -**  **DAY 36** | Female | Mean | 19.7 | 19.2 | 19.1 | 19.4^a^ | 18.7 | 20.2 |
|  |  | 95% CI | 17.9 - 21.5 | 17.4 - 21.0 | 17.3 - 20.9 | -- | -- | -- |
|  |  | Range | 17.4 - 22.7 | 15.1 - 21.4 | 17.0 - 25.1 | 16.5 - 22.7 | 15.5 - 21.9 | 16.9 - 24.0 |
|  | Male | Mean | 29.5 | 29.5 | 28.6 | 27.9 | 29.1 | 27.8 |
|  |  | 95% CI | 27.7 - 31.3 | 27.7 - 31.3 | 26.8 - 30.4 | -- | -- | -- |
|  |  | Range | 24.4 - 36.1 | 26.5 - 31.2 | 25.2 - 32.7 | 23.5 - 35.8 | 24.5 - 34.2 | 25.4 - 32.8 |
| **FC:**  **DAY 36 -**  **DAY 43** | Female | Mean | 19.0 | 19.3 | 18.6 | 18.8^a^ | 19.2 | 20.0 |
|  |  | 95% CI | 17.3 - 20.7 | 17.5 - 21.0 | 16.9 - 20.3 | -- | -- | -- |
|  |  | Range | 17.3 - 23.4 | 15.4 - 24.2 | 16.2 - 23.5 | 15.4 - 20.6 | 16.4 - 22.3 | 18.0 - 24.2 |
|  | Male | Mean | 30.1 | 29.1 | 28.4 | 26.4 | 27.8 | 27.9 |
|  |  | 95% CI | 28.4 - 31.8 | 27.4 - 30.8 | 26.6 - 30.1 | -- | -- | -- |
|  |  | Range | 26.1 - 33.0 | 26.4 - 32.1 | 25.0 - 33.1 | 22.6 - 31.6 | 25.0 - 32.8 | 24.2 - 34.5 |
| **FC:**  **DAY 43 -**  **51** | Female | Mean | 19.0 | 19.5 | 20.4 | 18.7^a^ | 19.7 | 19.2 |
|  |  | 95% CI | 17.1 - 20.8 | 17.7 - 21.3 | 18.6 - 22.2 | -- | -- | -- |
|  |  | Range | 17.4 - 20.9 | 15.6 - 26.5 | 17.8 - 25.0 | 14.9 - 21.3 | 14.8 - 24.1 | 17.3 - 21.5 |
|  | Male | Mean | 30.0 | 29.7 | 29.6 | 27.9 | 29.7 | 28.5 |
|  |  | 95% CI | 28.2 - 31.8 | 27.9 - 31.5 | 27.7 - 31.4 | -- | -- | -- |
|  |  | Range | 24.5 - 33.2 | 26.5 - 33.0 | 25.7 - 33.1 | 25.3 - 34.0 | 26.8 - 31.0 | 25.4 - 33.0 |
| **FC:**  **DAY 51 -**  **DAY 57** | Female | Mean | 18.3 | 20.4**! | 19.8 | 17.7^a^ | 18.4 | 19.0 |
|  |  | 95% CI | 16.6 - 20.1 | 18.7 - 22.1 | 18.1 - 21.5 | -- | -- | -- |
|  |  | Range | 17.0 - 20.2 | 15.3 - 24.3 | 17.4 - 23.5 | 14.3 - 20.6 | 17.0 - 20.7 | 16.9 - 21.3 |
|  | Male | Mean | 29.3 | 31.0! | 30.9 | 27.4 | 28.1 | 28.8 |
|  |  | 95% CI | 27.1 - 31.6 | 28.7 - 33.2 | 28.7 - 33.2 | -- | -- | -- |
|  |  | Range | 25.1 - 33.5 | 26.2 - 34.8 | 27.4 - 34.2 | 23.9 - 30.3 | 23.5 - 31.3 | 25.2 - 33.1 |
| **FC:**  **DAY 57 -**  **DAY 64** | Female | Mean | 19.9 | 18.7 | 18.8 | 18.5^a^ | 20.0 | 19.3 |
|  |  | 95% CI | 18.3 - 21.6 | 17.1 - 20.4 | 17.2 - 20.5 | -- | -- | -- |
|  |  | Range | 17.3 - 23.6 | 15.4 - 22.6 | 16.4 - 25.3 | 17.2 - 21.5 | 17.0 - 28.0 | 17.3 - 21.6 |
|  | Male | Mean | 30.5 | 30.4 | 30.1 | 27.7 | 29.5 | 28.9 |
|  |  | 95% CI | 28.9 - 32.2 | 28.8 - 32.1 | 28.5 - 31.8 | -- | -- | -- |
|  |  | Range | 28.4 - 32.6 | 27.0 - 32.1 | 26.6 - 33.4 | 24.1 - 32.0 | 25.8 - 33.9 | 25.5 - 35.1 |
| **FC:**  **DAY 64 -**  **DAY 71** | Female | Mean | 18.3 | 18.6 | 18.7 | 18.9^a^ | 18.6 | 18.8 |
|  |  | 95% CI | 16.7 - 19.9 | 17.0 - 20.2 | 17.1 - 20.3 | -- | -- | -- |
|  |  | Range | 16.2 - 21.2 | 15.2 - 22.8 | 16.4 - 21.0 | 15.6 - 23.1 | 16.2 - 23.3 | 17.2 - 20.4 |
|  | Male | Mean | 30.2 | 29.5 | 30.5 | 26.9 | 31.3 | 28.7 |
|  |  | 95% CI | 28.6 - 31.8 | 27.9 - 31.1 | 28.9 - 32.1 | -- | -- | -- |
|  |  | Range | 26.8 - 32.9 | 26.3 - 33.2 | 24.9 - 34.1 | 23.6 - 32.0 | 26.6 - 33.5 | 25.4 - 33.4 |

**Table S5. Food Consumption and Food Efficiency Summary Rats fed Maize Source Diets (continued)**

|  | **Gender** | **Statistical Parameter** | **Control** | **DP23211**  **High** | **DP23211**  **Low** | **P0928** | **P0993** | **P1105** |
| --- | --- | --- | --- | --- | --- | --- | --- | --- |
|  | **Food Consumption (FC) (g/day) (continued)** | | | | | | | |
| **FC:**  **DAY 71 -**  **DAY 78** | Female | Mean | 17.9 | 18.9 | 18.4 | 18.5^a^ | 17.4 | 18.3 |
|  |  | 95% CI | 16.4 - 19.5 | 17.4 - 20.5 | 16.9 - 19.9 | -- | -- | -- |
|  |  | Range | 16.2 - 20.5 | 15.3 - 21.6 | 14.8 - 23.4 | 15.6 - 28.5 | 14.3 - 19.6 | 16.6 - 21.6 |
|  | Male | Mean | 29.7 | 29.7 | 31.0 | 27.1 | 29.6 | 27.3 |
|  |  | 95% CI | 28.1 - 31.2 | 28.2 - 31.3 | 29.4 - 32.5 | -- | -- | -- |
|  |  | Range | 27.6 - 32.8 | 27.8 - 31.0 | 25.6 - 33.6 | 22.9 - 30.4 | 27.1 - 31.9 | 24.7 - 31.3 |
| **FC:**  **DAY 78 -**  **DAY 85** | Female | Mean | 17.5 | 19.6 | 17.3 | 16.8^a^ | 17.7 | 18.2 |
|  |  | 95% CI | 15.0 - 20.0 | 17.1 - 22.0 | 14.8 - 19.7 | -- | -- | -- |
|  |  | Range | 15.1 - 20.2 | 15.2 - 22.4 | 11.4 - 24.4 | 12.4 - 19.6 | 14.1 - 22.9 | 16.2 - 20.3 |
|  | Male | Mean | 27.6 | 29.3 | 30.9 | 28.2 | 29.3 | 27.4 |
|  |  | 95% CI | 25.1 - 30.0 | 26.8 - 31.7 | 28.5 - 33.4 | -- | -- | -- |
|  |  | Range | 13.5 - 34.1 | 26.8 - 31.0 | 25.7 - 34.2 | 23.7 - 33.7 | 24.9 - 33.9 | 25.2 - 30.7 |
| **FC:**  **DAY 85 -**  **DAY 91** | Female | Mean | 17.7 | 18.3 | 18.9 | 17.2^a^ | 17.7 | 17.0 |
|  |  | 95% CI | 15.5 - 19.8 | 16.2 - 20.5 | 16.8 - 21.1 | -- | -- | -- |
|  |  | Range | 16.3 - 21.1 | 14.7 - 20.4 | 15.1 - 29.2 | 15.5 - 20.2 | 14.5 - 24.5 | 15.7 - 18.2 |
|  | Male | Mean | 29.7 | 31.3^b^ | 30.3 | 27.3 | 29.8 | 26.6 |
|  |  | 95% CI | 27.5 - 31.8 | 29.1 - 33.5 | 28.2 - 32.5 | -- | -- | -- |
|  |  | Range | 26.5 - 35.9 | 27.4 - 36.1 | 25.8 - 33.5 | 23.4 - 33.5 | 27.9 - 31.1 | 23.6 - 32.1 |
| **FC:**  **DAY 1 -**  **DAY 91** | Female | Mean | 18.6 | 19.2 | 18.9 | 18.5^a^ | 18.5 | 19.3 |
|  |  | 95% CI | 17.3 - 19.9 | 17.9 - 20.6 | 17.6 - 20.2 | -- | -- | -- |
|  |  | Range | 17.2 - 20.3 | 15.5 - 22.4 | 17.1 - 21.7 | 16.1 - 20.8 | 15.6 - 19.8 | 17.4 - 20.8 |
|  | Male | Mean | 28.8 | 29.2 | 29.2 | 27.3 | 28.6 | 27.4 |
|  |  | 95% CI | 27.5 - 30.1 | 27.9 - 30.5 | 27.9 - 30.5 | -- | -- | -- |
|  |  | Range | 26.4 - 32.5 | 27.2 - 31.1 | 25.8 - 31.9 | 24.1 - 33.0 | 26.2 - 29.9 | 24.9 - 31.2 |
|  | **Food Efficiency (FE)** | | | | | | | |
| **FE:**  **DAY 1 -**  **DAY 8** | Female | Mean | 0.210 | 0.164 | 0.196 | 0.169 | 0.209 | 0.163 |
|  |  | 95% CI | 0.176 - 0.244 | 0.130 - 0.197 | 0.162 - 0.230 | -- | -- | -- |
|  |  | Range | 0.152 - 0.266 | 0.110 - 0.217 | 0.091 - 0.267 | 0.091 - 0.218 | 0.150 - 0.268 | 0.073 - 0.278 |
|  | Male | Mean | 0.344 | 0.355 | 0.357 | 0.347 | 0.356 | 0.351 |
|  |  | 95% CI | 0.331 - 0.357 | 0.342 - 0.369 | 0.344 - 0.370 | -- | -- | -- |
|  |  | Range | 0.316 - 0.367 | 0.326 - 0.386 | 0.335 - 0.372 | 0.325 - 0.374 | 0.341 - 0.368 | 0.334 - 0.369 |
| **FE:**  **DAY 8 -**  **DAY 15** | Female | Mean | 0.205 | 0.165**! | 0.172** | 0.200^a^ | 0.179 | 0.175 |
|  |  | 95% CI | 0.185 - 0.226 | 0.144 - 0.185 | 0.152 - 0.193 | -- | -- | -- |
|  |  | Range | 0.157 - 0.249 | 0.135 - 0.189 | 0.110 - 0.236 | 0.147 - 0.275 | 0.121 - 0.242 | 0.109 - 0.234 |
|  | Male | Mean | 0.309 | 0.290! | 0.308 | 0.280 | 0.298 | 0.293 |
|  |  | 95% CI | 0.289 - 0.329 | 0.269 - 0.310 | 0.287 - 0.328 | -- | -- | -- |
|  |  | Range | 0.295 - 0.349 | 0.229 - 0.347 | 0.286 - 0.319 | 0.238 - 0.318 | 0.270 - 0.323 | 0.252 - 0.312 |
| **FE:**  **DAY 15 -**  **DAY 22** | Female | Mean | 0.124 | 0.099 | 0.141 | 0.101^a^ | 0.130 | 0.128 |
|  |  | 95% CI | 0.095 - 0.154 | 0.070 - 0.129 | 0.112 - 0.170 | -- | -- | -- |
|  |  | Range | 0.064 - 0.166 | 0.067 - 0.144 | 0.071 - 0.203 | -0.010 - 0.193 | 0.077 - 0.172 | 0.037 - 0.204 |
|  | Male | Mean | 0.218 | 0.218 | 0.224 | 0.212 | 0.211 | 0.215 |
|  |  | 95% CI | 0.199 - 0.236 | 0.200 - 0.236 | 0.206 - 0.242 | -- | -- | -- |
|  |  | Range | 0.179 - 0.239 | 0.180 - 0.256 | 0.191 - 0.257 | 0.183 - 0.235 | 0.184 - 0.224 | 0.199 - 0.236 |
| **FE:**  **DAY 22 -**  **DAY 29** | Female | Mean | 0.089 | 0.082 | 0.086 | 0.101^a^ | 0.094 | 0.092 |
|  |  | 95% CI | 0.069 - 0.109 | 0.062 - 0.101 | 0.067 - 0.106 | -- | -- | -- |
|  |  | Range | 0.047 - 0.117 | 0.029 - 0.125 | 0.040 - 0.128 | 0.057 - 0.141 | 0.048 - 0.126 | 0.036 - 0.134 |
|  | Male | Mean | 0.192 | 0.193 | 0.191 | 0.172 | 0.189 | 0.194 |
|  |  | 95% CI | 0.172 - 0.212 | 0.173 - 0.212 | 0.171 - 0.211 | -- | -- | -- |
|  |  | Range | 0.149 - 0.225 | 0.163 - 0.243 | 0.161 - 0.225 | 0.136 - 0.197 | 0.157 - 0.212 | 0.158 - 0.246 |
| **FE:**  **DAY 29 -**  **DAY 36** | Female | Mean | 0.092 | 0.094 | 0.097 | 0.057^a^ | 0.069 | 0.068 |
|  |  | 95% CI | 0.066 - 0.117 | 0.069 - 0.120 | 0.072 - 0.123 | -- | -- | -- |
|  |  | Range | 0.044 - 0.135 | 0.031 - 0.152 | 0.067 - 0.133 | 0.017 - 0.127 | -0.016 - 0.133 | 0.016 - 0.157 |
|  | Male | Mean | 0.151 | 0.140 | 0.149 | 0.132 | 0.137 | 0.146 |
|  |  | 95% CI | 0.134 - 0.169 | 0.123 - 0.158 | 0.131 - 0.167 | -- | -- | -- |
|  |  | Range | 0.128 - 0.195 | 0.111 - 0.162 | 0.110 - 0.187 | 0.072 - 0.176 | 0.111 - 0.151 | 0.118 - 0.185 |
| **FE:**  **DAY 36 -**  **DAY 43*** | Female | Mean | 0.100 | 0.089 | 0.072 | 0.101^a^ | 0.091 | 0.087 |
|  |  | 95% CI | 0.080 - 0.119 | 0.069 - 0.108 | 0.053 - 0.092 | -- | -- | -- |
|  |  | Range | 0.060 - 0.129 | 0.026 - 0.125 | 0.026 - 0.111 | 0.037 - 0.165 | 0.026 - 0.166 | 0.040 - 0.109 |
|  | Male | Mean | 0.131 | 0.125 | 0.142 | 0.130 | 0.117 | 0.125 |
|  |  | 95% CI | 0.118 - 0.143 | 0.113 - 0.138 | 0.129 - 0.155 | -- | -- | -- |
|  |  | Range | 0.109 - 0.161 | 0.101 - 0.153 | 0.123 - 0.163 | 0.109 - 0.182 | 0.089 - 0.166 | 0.093 - 0.168 |

**Table S5. Food Consumption and Food Efficiency Summary Rats fed Maize Source Diets (continued)**

|  | **Gender** | **Statistical Parameter** | **Control** | **DP23211**  **High** | **DP23211**  **Low** | **P0928** | **P0993** | **P1105** |
| --- | --- | --- | --- | --- | --- | --- | --- | --- |
|  | **Food Efficiency (FE) (g/day) (continued)** | | | | | | | |
| **FE:**  **DAY 43 -**  **DAY 51** | Female | Mean | 0.068 | 0.055 | 0.072 | 0.060^a^ | 0.047 | 0.059 |
|  |  | 95% CI | 0.049 - 0.086 | 0.036 - 0.073 | 0.054 - 0.091 | -- | -- | -- |
|  |  | Range | 0.036 - 0.103 | 0.016 - 0.088 | 0.014 - 0.157 | 0.012 - 0.086 | 0.020 - 0.090 | 0.028 - 0.088 |
|  | Male | Mean | 0.116 | 0.100 | 0.113 | 0.113 | 0.115 | 0.110 |
|  |  | 95% CI | 0.098 - 0.135 | 0.081 - 0.118 | 0.095 - 0.132 | -- | -- | -- |
|  |  | Range | 0.074 - 0.148 | 0.080 - 0.127 | 0.092 - 0.154 | 0.090 - 0.155 | 0.059 - 0.159 | 0.069 - 0.149 |
| **FE:**  **DAY 51 -**  **DAY 57** | Female | Mean | 0.007 | 0.036 | 0.052 | 0.039^a^ | 0.050 | 0.067 |
|  |  | 95% CI | -0.030 - 0.044 | -0.001 - 0.073 | 0.016 - 0.089 | -- | -- | -- |
|  |  | Range | -0.066 - 0.126 | -0.007 - 0.096 | -0.002 - 0.134 | -0.101 - 0.184 | -0.076 - 0.173 | -0.026 - 0.146 |
|  | Male | Mean | 0.118 | 0.089 | 0.112 | 0.095 | 0.108 | 0.117 |
|  |  | 95% CI | 0.097 - 0.138 | 0.069 - 0.110 | 0.091 - 0.133 | -- | -- | -- |
|  |  | Range | 0.048 - 0.168 | 0.059 - 0.121 | 0.066 - 0.140 | 0.056 - 0.125 | 0.088 - 0.119 | 0.078 - 0.147 |
| **FE:**  **DAY 57 -**  **DAY 64** | Female | Mean | 0.083 | 0.057 | 0.057 | 0.068^a^ | 0.043 | 0.048 |
|  |  | 95% CI | 0.055 - 0.110 | 0.030 - 0.084 | 0.030 - 0.084 | -- | -- | -- |
|  |  | Range | 0.018 - 0.180 | 0.028 - 0.095 | 0.014 - 0.110 | -0.024 - 0.182 | -0.040 - 0.124 | 0.005 - 0.117 |
|  | Male | Mean | 0.087 | 0.075 | 0.089 | 0.090 | 0.070 | 0.081 |
|  |  | 95% CI | 0.072 - 0.102 | 0.060 - 0.090 | 0.074 - 0.104 | -- | -- | -- |
|  |  | Range | 0.048 - 0.118 | 0.050 - 0.106 | 0.065 - 0.115 | 0.053 - 0.108 | 0.033 - 0.102 | -0.006 - 0.111 |
| **FE:**  **DAY 64 -**  **DAY 71** | Female | Mean | 0.048 | 0.070 | 0.050 | 0.047^a^ | 0.060 | 0.036 |
|  |  | 95% CI | 0.019 - 0.077 | 0.041 - 0.098 | 0.021 - 0.079 | -- | -- | -- |
|  |  | Range | -0.045 - 0.110 | 0.041 - 0.113 | -0.010 - 0.116 | 0.003 - 0.103 | 0.009 - 0.105 | -0.028 - 0.078 |
|  | Male | Mean | 0.055 | 0.060 | 0.068 | 0.067 | 0.049 | 0.066 |
|  |  | 95% CI | 0.037 - 0.072 | 0.042 - 0.077 | 0.051 - 0.085 | -- | -- | -- |
|  |  | Range | 0.028 - 0.077 | 0.027 - 0.096 | 0.006 - 0.118 | 0.047 - 0.085 | 0.038 - 0.071 | 0.041 - 0.086 |
| **FE:**  **DAY 71 -**  **DAY 78** | Female | Mean | 0.039 | 0.026 | 0.030 | 0.052^a^ | 0.051 | 0.048 |
|  |  | 95% CI | 0.006 - 0.072 | -0.006 - 0.059 | -0.003 - 0.063 | -- | -- | -- |
|  |  | Range | -0.048 - 0.134 | -0.025 - 0.069 | -0.045 - 0.112 | -0.003 - 0.108 | -0.017 - 0.092 | -0.022 - 0.094 |
|  | Male | Mean | 0.073 | 0.058 | 0.080 | 0.085 | 0.088 | 0.085 |
|  |  | 95% CI | 0.057 - 0.089 | 0.042 - 0.074 | 0.064 - 0.096 | -- | -- | -- |
|  |  | Range | 0.034 - 0.093 | 0.019 - 0.085 | 0.033 - 0.112 | 0.072 - 0.103 | 0.066 - 0.114 | 0.044 - 0.105 |
| **FE:**  **DAY 78 -**  **DAY 85** | Female | Mean | -0.043 | -0.014 | -0.026 | -0.033^a^ | -0.035 | 0.004 |
|  |  | 95% CI | -0.076 - -0.010 | -0.047 - 0.019 | -0.058 - 0.007 | -- | -- | -- |
|  |  | Range | -0.126 - 0.045 | -0.058 - 0.063 | -0.068 - 0.009 | -0.177 - 0.058 | -0.091 - 0.002 | -0.045 - 0.040 |
|  | Male | Mean | 0.047 | 0.042 | 0.031 | 0.027 | 0.030 | 0.019 |
|  |  | 95% CI | 0.024 - 0.069 | 0.019 - 0.064 | 0.008 - 0.054 | -- | -- | -- |
|  |  | Range | -0.017 - 0.113 | 0.005 - 0.069 | -0.012 - 0.086 | -0.022 - 0.075 | -0.014 - 0.071 | -0.033 - 0.060 |
| **FE:**  **DAY 85 -**  **DAY 91** | Female | Mean | 0.064 | 0.045 | 0.052 | 0.018^a^ | 0.033 | -0.001 |
|  |  | 95% CI | 0.024 - 0.103 | 0.006 - 0.084 | 0.013 - 0.092 | -- | -- | -- |
|  |  | Range | 0.006 - 0.141 | -0.020 - 0.105 | -0.059 - 0.128 | -0.140 - 0.241 | -0.067 - 0.144 | -0.073 - 0.109 |
|  | Male | Mean | 0.031 | 0.037 | 0.044 | 0.037 | 0.032 | 0.024 |
|  |  | 95% CI | 0.007 - 0.056 | 0.012 - 0.061 | 0.019 - 0.068 | -- | -- | -- |
|  |  | Range | -0.063 - 0.075 | -0.008 - 0.058 | 0.021 - 0.084 | -0.006 - 0.117 | 0.004 - 0.078 | -0.025 - 0.094 |
| **FE:**  **DAY 1 -**  **DAY 91** | Female | Mean | 0.085 | 0.075**! | 0.082 | 0.077^a^ | 0.079 | 0.078 |
|  |  | 95% CI | 0.080 - 0.090 | 0.069 - 0.080 | 0.076 - 0.087 | -- | -- | -- |
|  |  | Range | 0.077 - 0.092 | 0.064 - 0.086 | 0.068 - 0.092 |  |  |  |
|  | Male | Mean | 0.140 | 0.134! | 0.143 | 0.137 | 0.136 | 0.139 |
|  |  | 95% CI | 0.133 - 0.148 | 0.126 - 0.141 | 0.135 - 0.150 | -- | -- | -- |
|  |  | Range | 0.122 - 0.154 | 0.121 - 0.152 | 0.124 - 0.157 | 0.125 - 0.147 | 0.129 - 0.140 | 0.119 - 0.152 |

Note: Test and control means are the LS-Means from the linear mixed model. Reference means are the arithmetic means. When a reported statistic was estimated to be < 10^-10^ in absolute value, the value was set to zero.

^a^	n=7, otherwise n=8

^b^	Average food consumption of two individual rats that were caged separately on day 84 was used as food consumption for the cage for the remainder of the study.

*	Sex x Treatment interaction statistically significant at the 0.05 level

**	Statistically significant difference when compared to control at the 0.05 level; FDR not significantly different

!	Combined statistically significant difference when compared to combined control at the 0.05 level; combined FDR not significantly different

**Table S6. Functional Observational Battery (FOB) Scores (Week 13) of Rats fed Maize Source Diets**

|  | **Gender** | **Score** | **Control** | **DP23211**  **High** | **DP23211**  **Low** | **P0928^a^** | **P0993** | **P1105** |
| --- | --- | --- | --- | --- | --- | --- | --- | --- |
| **Righting Reflex** | Female | Present | 16 | 16 | 16 | 15 | 16 | 16 |
|  | Male |  | 16 | 16 | 16 | 16 | 16 | 16 |
| **Approach & Touch** | Female | No Reaction | 16 | 16 | 16 | 15 | 16 | 16 |
|  | Male |  | 16 | 16 | 16 | 16 | 16 | 16 |
| **Auditory Stimulus** | Female | No Reaction | 16 | 16 | 16 | 15 | 16 | 16 |
|  | Male |  | 16 | 16 | 16 | 16 | 16 | 16 |
| **Tail Pinch** | Female | No Response | 16 | 16 | 16 | 15 | 16 | 16 |
|  | Male |  | 16 | 16 | 16 | 16 | 16 | 16 |
| **Pupillary Response** | Female | Present | 16 | 16 | 16 | 15 | 16 | 16 |
|  | Male |  | 16 | 16 | 16 | 16 | 16 | 16 |
| **Diarrhea** | Female | Absent | 16 | 16 | 16 | 15 | 16 | 16 |
|  | Male |  | 16 | 16 | 16 | 16 | 16 | 16 |
| **Polyuria** | Female | Absent | 16 | 16 | 16 | 15 | 16 | 16 |
|  | Male |  | 16 | 16 | 16 | 16 | 16 | 16 |

^a^	n=15; females only, otherwise n=16

**Table S7. Neurobehavioral Summary of Rats fed Maize Source Diets**

|  | **Gender** | **Statistical Parameter** | **Control** | **DP23211**  **High** | **DP23211**  **Low** | **P0928^a^** | **P0993** | **P1105** |
| --- | --- | --- | --- | --- | --- | --- | --- | --- |
|  | **Forelimb and Hindlimb Grip Strength (week 13)** | | | | | | | |
| **Forelimb**  **Grip**  **Strength**  **(kg)** | Female | Mean | 1.59 | 1.61 | 1.55 | 1.48 | 1.62 | 1.62 |
|  |  | 95% CI | 1.42 - 1.75 | 1.45 - 1.78 | 1.38 - 1.72 | -- | -- | -- |
|  |  | Range | 1.00 - 2.27 | 1.30 - 2.03 | 1.04 - 1.92 | 0.88 - 2.34 | 1.18 - 2.17 | 1.26 - 1.93 |
|  | Male | Mean | 1.94 | 2.11 | 2.11 | 1.91 | 2.00 | 2.05 |
|  |  | 95% CI | 1.78 - 2.11 | 1.94 - 2.28 | 1.94 - 2.28 | -- | -- | -- |
|  |  | Range | 1.42 - 2.46 | 1.57 - 2.55 | 1.50 - 2.60 | 0.90 - 2.43 | 1.36 - 2.42 | 1.40 - 2.54 |
| **Hindlimb**  **Grip**  **Strength**  **(kg)** | Female | Mean | 1.29 | 1.16 | 1.20 | 1.26 | 1.22 | 1.26 |
|  |  | 95% CI | 1.17 - 1.41 | 1.05 - 1.28 | 1.08 - 1.31 | -- | -- | -- |
|  |  | Range | 0.99 - 1.58 | 0.77 - 1.58 | 0.92 - 1.51 | 0.88 - 1.76 | 0.98 - 1.60 | 0.84 - 1.60 |
|  | Male | Mean | 1.46 | 1.51 | 1.43 | 1.49 | 1.49 | 1.49 |
|  |  | 95% CI | 1.34 - 1.57 | 1.40 - 1.63 | 1.32 - 1.55 | -- | -- | -- |
|  |  | Range | 1.07 - 1.89 | 1.30 - 1.73 | 1.03 - 1.87 | 1.02 - 2.04 | 1.14 - 1.83 | 1.15 - 1.87 |
|  | **Motor Activity Assessment (week 13)** | | | | | | | |
| **Movement Duration (sec)** | | | | | | | | |
| **Movement**  **Duration**  **Interval 1^#^**  **(sec)** | Female | Mean | 360 | 380 | 338 | 350 | 354 | 354 |
|  |  | 95% CI | 337 - 381 | 358 - 400 | 313 - 361 | -- | -- | -- |
|  |  | Range | 245 - 476 | 298 - 430 | 188 - 386 | 241 - 436 | 178 - 430 | 221 - 428 |
|  | Male | Mean | 317 | 335 | 336 | 343 | 332 | 330 |
|  |  | 95% CI | 291 - 342 | 310 - 358 | 311 - 359 | -- | -- | -- |
|  |  | Range | 140 - 395 | 172 - 400 | 205 - 409 | 195 - 407 | 261 - 390 | 218 - 427 |
| **Movement Duration Interval 2**  **(sec)*** | Female | Mean | 189 | 267** | 243** | 257 | 241 | 248 |
|  |  | 95% CI | 151 - 227 | 229 - 304 | 205 - 281 | -- | -- | -- |
|  |  | Range | 22 - 321 | 152 - 352 | 102 - 358 | 139 - 384 | 51 - 356 | 145 - 365 |
|  | Male | Mean | 216 | 198 | 192 | 235 | 217 | 201 |
|  |  | 95% CI | 178 - 254 | 160 - 235 | 154 - 230 | -- | -- | -- |
|  |  | Range | 49 - 357 | 51 - 314 | 11 - 295 | 49 - 359 | 138 - 299 | 6 - 339 |
| **Movement Duration**  **Interval 3**  **(sec)** | Female | Mean | 159 | 228** | 191 | 205 | 197 | 194 |
|  |  | 95% CI | 121 - 198 | 190 - 267 | 153 - 230 | -- | -- | -- |
|  |  | Range | 1 - 302 | 92 - 374 | 51 - 394 | 64 - 336 | 67 - 298 | 4 - 326 |
|  | Male | Mean | 163 | 155 | 138 | 187 | 143 | 177 |
|  |  | 95% CI | 125 - 202 | 117 - 194 | 100 - 177 | -- | -- | -- |
|  |  | Range | 6 - 266 | 4 - 241 | 6 - 252 | 39 - 304 | 16 - 253 | 36 - 287 |
| **Movement Duration**  **Interval 4**  **(sec)** | Female | Mean | 200 | 199 | 170 | 156 | 187 | 183 |
|  |  | 95% CI | 154 - 246 | 153 - 245 | 124 - 216 | -- | -- | -- |
|  |  | Range | 12 - 341 | 11 - 366 | 8 - 300 | 0 - 347 | 51 - 422 | 47 - 305 |
|  | Male | Mean | 111 | 127 | 135 | 168 | 155 | 125 |
|  |  | 95% CI | 65 - 157 | 81 - 173 | 89 - 181 | -- | -- | -- |
|  |  | Range | 3 - 245 | 11 - 267 | 0 - 285 | 8 - 296 | 0 - 305 | 0 - 277 |
| **Movement Duration Interval 5**  **(sec)** | Female | Mean | 198 | 164 | 184 | 168 | 206 | 179 |
|  |  | 95% CI | 151 - 245 | 117 - 211 | 137 - 230 | -- | -- | -- |
|  |  | Range | 85 - 313 | 22 - 312 | 4 - 281 | 3 - 307 | 22 - 303 | 22 - 322 |
|  | Male | Mean | 103 | 129 | 126 | 128 | 152 | 131 |
|  |  | 95% CI | 56 - 149 | 83 - 176 | 79 - 172 | -- | -- | -- |
|  |  | Range | 2 - 251 | 16 - 265 | 2 - 267 | 1 - 244 | 0 - 276 | 0 - 290 |
| **Movement Duration**  **Interval 6**  **(sec)** | Female | Mean | 126 | 136 | 167 | 173 | 179 | 160 |
|  |  | 95% CI | 76 - 176 | 86 - 186 | 117 - 217 | -- | -- | -- |
|  |  | Range | 0 - 279 | 4 - 261 | 3 - 299 | 24 - 325 | 26 - 300 | 25 - 279 |
|  | Male | Mean | 80 | 122 | 115 | 118 | 114 | 139 |
|  |  | 95% CI | 30 - 130 | 72 - 172 | 66 - 165 | -- | -- | -- |
|  |  | Range | 0 - 271 | 0 - 302 | 2 - 340 | 5 - 277 | 0 - 278 | 0 - 314 |
| **Movement Duration**  **Total**  **(sec)** | Female | Mean | 1228 | 1372 | 1289 | 1308 | 1364 | 1317 |
|  |  | 95% CI | 1097 - 1360 | 1240 - 1503 | 1157 - 1420 | -- | -- | -- |
|  |  | Range | 933 - 1666 | 971 - 1708 | 862 - 2008 | 725 - 1916 | 876 - 1763 | 652 - 1811 |
|  | Male | Mean | 982 | 1062 | 1038 | 1180 | 1113 | 1103 |
|  |  | 95% CI | 851 - 1114 | 930 - 1193 | 907 - 1170 | -- | -- | -- |
|  |  | Range | 497 - 1584 | 789 - 1370 | 565 - 1309 | 505 - 1597 | 593 - 1534 | 430 - 1400 |

**Table S7. Neurobehavioral Summary of Rats fed Maize Source Diets (continued)**

|  | **Gender** | **Statistical Parameter** | **Control** | **DP23211**  **High** | **DP23211**  **Low** | **P0928^a^** | **P0993** | **P1105** |
| --- | --- | --- | --- | --- | --- | --- | --- | --- |
| **Number of Movements (week 13)** | | | | | | | | |
| **Number**  **of**  **Movements**  **Interval 1**  **(count)** | Female | Mean | 362 | 418 | 318 | 365 | 383 | 368 |
|  |  | 95% CI | 288 - 435 | 344 - 491 | 244 - 392 | -- | -- | -- |
|  |  | Range | 159 - 594 | 278 - 585 | 119 - 508 | 146 - 587 | 91 - 635 | 211 - 586 |
|  | Male | Mean | 166 | 188 | 168 | 177 | 184 | 152 |
|  |  | 95% CI | 127 - 205 | 149 - 227 | 129 - 207 | -- | -- | -- |
|  |  | Range | 35 - 293 | 100 - 302 | 31 - 334 | 66 - 322 | 86 - 286 | 58 - 250 |
| **Number**  **of Movements Interval 2^#^**  **(count)** | Female | Mean | 103 | 190**! | 153 | 203 | 176 | 158 |
|  |  | 95% CI | 71 - 141 | 145 - 241 | 113 - 198 | -- | -- | -- |
|  |  | Range | 0 - 401 | 46 - 398 | 38 - 450 | 51 - 560 | 2 - 329 | 22 - 344 |
|  | Male | Mean | 51 | 54! | 44 | 80 | 69 | 54 |
|  |  | 95% CI | 32 - 74 | 35 - 78 | 27 - 65 | -- | -- | -- |
|  |  | Range | 10 - 134 | 5 - 136 | 0 - 118 | 2 - 177 | 27 - 126 | 0 - 98 |
| **Number**  **of**  **Movements**  **Interval 3^#^**  **(count)** | Female | Mean | 76 | 156**! | 91 | 154 | 104 | 148 |
|  |  | 95% CI | 37 - 127 | 98 - 227 | 48 - 147 | -- | -- | -- |
|  |  | Range | 0 - 310 | 41 - 309 | 0 - 503 | 22 - 422 | 1 - 248 | 0 - 370 |
|  | Male | Mean | 30 | 37! | 34 | 63 | 39 | 55 |
|  |  | 95% CI | 15 - 50 | 20 - 60 | 18 - 56 | -- | -- | -- |
|  |  | Range | 0 - 137 | 0 - 81 | 0 - 118 | 0 - 146 | 0 - 94 | 0 - 118 |
| **Number**  **of Movements**  **Interval 4^#^**  **(count)** | Female | Mean | 114 | 110 | 96 | 99 | 140 | 147 |
|  |  | 95% CI | 64 - 179 | 60 - 173 | 50 - 156 | -- | -- | -- |
|  |  | Range | 0 - 285 | 0 - 365 | 0 - 324 | 0 - 462 | 6 - 760 | 0 - 383 |
|  | Male | Mean | 17 | 37 | 35 | 60 | 54 | 31 |
|  |  | 95% CI | 4 - 39 | 15 - 67 | 14 - 66 | -- | -- | -- |
|  |  | Range | 0 - 112 | 0 - 125 | 0 - 240 | 0 - 134 | 0 - 145 | 0 - 79 |
| **Number**  **of Movements**  **Interval 5**  **(count)** | Female | Mean | 126 | 117 | 125 | 124 | 150 | 119 |
|  |  | 95% CI | 77 - 174 | 68 - 165 | 76 - 174 | -- | -- | -- |
|  |  | Range | 7 - 218 | 0 - 306 | 0 - 308 | 0 - 293 | 0 - 287 | 8 - 358 |
|  | Male | Mean | 28 | 48 | 35 | 38 | 41 | 52 |
|  |  | 95% CI | 6 - 50 | 25 - 70 | 13 - 57 | -- | -- | -- |
|  |  | Range | 0 - 96 | 0 - 163 | 0 - 122 | 0 - 94 | 0 - 107 | 0 - 183 |
| **Number**  **of Movements**  **Interval 6**  **(count)** | Female | Mean | 73 | 111 | 125 | 132 | 125 | 93 |
|  |  | 95% CI | 18 - 129 | 55 - 166 | 69 - 180 | -- | -- | -- |
|  |  | Range | 0 - 226 | 0 - 346 | 0 - 331 | 4 - 424 | 0 - 273 | 0 - 256 |
|  | Male | Mean | 21 | 51** | 37 | 41 | 42 | 53 |
|  |  | 95% CI | -1 - 42 | 29 - 72 | 15 - 58 | -- | -- | -- |
|  |  | Range | 0 - 132 | 0 - 160 | 0 - 133 | 0 - 197 | 0 - 173 | 0 - 156 |
| **Number of Movements**  **Total^#^**  **(count)** | Female | Mean | 860 | 1092! | 877 | 1077 | 1078 | 1032 |
|  |  | 95% CI | 672 - 1099 | 854 - 1397 | 686 - 1122 | -- | -- | -- |
|  |  | Range | 417 - 1702 | 616 - 2120 | 475 - 2363 | 379 - 2537 | 383 - 1798 | 290 - 2047 |
|  | Male | Mean | 303 | 428! | 337 | 458 | 429 | 397 |
|  |  | 95% CI | 237 - 387 | 335 - 548 | 263 - 431 | -- | -- | -- |
|  |  | Range | 113 - 685 | 266 - 641 | 82 - 758 | 145 - 901 | 224 - 657 | 111 - 560 |

Note: When a reported statistic was estimated to be < 10^-10^ in absolute value, the value was set to zero.

^a^	n=15; females only, otherwise n=16

^#^	Test and control data were transformed prior to analysis. The means are presented on the data scale (back-transformed), confidence interval (CI) are presented on the transformed scale. Reference arithmetic means were calculated from the raw data.

*	Sex x Treatment interaction statistically significant at the 0.05 level

**	Statistically significant difference when compared to control at the 0.05 level; FDR not significantly different

!	Combined statistically significant difference when compared to combined control at the 0.05 level; combined FDR not significantly different

**Table S8. Hematology Summary of Rats fed Maize Source Diets**

|  | **Gender** | **Statistical Parameter** | **Control^a^** | **DP23211**  **High** | **DP23211**  **Low^a^** | **P0928^a^** | **P0993** | **P1105** |
| --- | --- | --- | --- | --- | --- | --- | --- | --- |
| **RBC**  **(×10^6^/μL)** | Female | Mean | 7.66 | 7.70 | 7.64 | 7.91 | 7.78 | 7.73 |
|  |  | 95% CI | 7.43 - 7.89 | 7.47 - 7.92 | 7.41 - 7.87 | -- | -- | -- |
|  |  | Range | 6.99 - 8.48 | 6.77 - 8.09 | 7.10 - 8.71 | 7.32 - 8.40 | 7.01 - 8.68 | 7.10 - 8.84 |
|  | Male | Mean | 8.48 | 8.52 | 8.57 | 8.52 | 8.78 | 8.61 |
|  |  | 95% CI | 8.26 - 8.71 | 8.30 - 8.75 | 8.34 - 8.79 | -- | -- | -- |
|  |  | Range | 8.00 - 9.30 | 7.73 - 9.81 | 7.86 - 9.13 | 8.08 - 9.16 | 8.08 - 9.44 | 7.98 - 9.63 |
| **HGB**  **(g/dL)** | Female | Mean | 13.8 | 13.6 | 13.7 | 14.1 | 13.9 | 13.8 |
|  |  | 95% CI | 13.5 - 14.2 | 13.3 - 14.0 | 13.3 - 14.1 | -- | -- | -- |
|  |  | Range | 12.2 - 15.5 | 12.5 - 14.4 | 12.8 - 14.9 | 12.9 - 15.1 | 12.5 - 15.4 | 12.5 - 16.2 |
|  | Male | Mean | 14.4 | 14.6 | 14.5 | 14.6 | 14.5 | 14.6 |
|  |  | 95% CI | 14.0 - 14.7 | 14.2 - 14.9 | 14.1 - 14.8 | -- | -- | -- |
|  |  | Range | 13.1 - 15.5 | 13.2 - 16.5 | 13.5 - 15.3 | 13.7 - 16.2 | 13.7 - 15.3 | 13.6 - 15.9 |
| **HCT**  **(%)** | Female | Mean | 42.4 | 42.0 | 42.4 | 43.6 | 42.5 | 42.8 |
|  |  | 95% CI | 41.2 - 43.5 | 40.9 - 43.2 | 41.2 - 43.5 | -- | -- | -- |
|  |  | Range | 37.3 - 45.3 | 39.1 - 44.6 | 39.7 - 46.5 | 40.0 - 46.7 | 38.2 - 48.3 | 38.4 - 50.7 |
|  | Male | Mean | 45.4 | 46.0 | 45.5 | 45.7 | 45.6 | 46.1 |
|  |  | 95% CI | 44.2 - 46.5 | 44.8 - 47.1 | 44.3 - 46.6 | -- | -- | -- |
|  |  | Range | 42.1 - 50.2 | 41.8 - 51.6 | 41.7 - 48.7 | 43.7 - 48.3 | 43.5 - 48.2 | 43.3 - 50.7 |
| **MCV**  **(fL)** | Female | Mean | 55.4 | 54.6 | 55.5 | 55.1 | 54.7 | 55.4 |
|  |  | 95% CI | 54.5 - 56.2 | 53.8 - 55.4 | 54.7 - 56.3 | -- | -- | -- |
|  |  | Range | 52.3 - 58.2 | 52.6 - 58.6 | 53.2 - 58.5 | 52.8 - 58.5 | 52.7 - 58.6 | 52.5 - 57.4 |
|  | Male | Mean | 53.5 | 54.0 | 53.1 | 53.6 | 51.9 | 53.6 |
|  |  | 95% CI | 52.7 - 54.3 | 53.2 - 54.8 | 52.2 - 53.9 | -- | -- | -- |
|  |  | Range | 50.8 - 56.0 | 50.1 - 58.2 | 50.8 - 55.3 | 51.3 - 56.0 | 48.4 - 54.3 | 49.1 - 57.0 |
| **MCH**  **(pg)** | Female | Mean | 18.1 | 17.7 | 18.0 | 17.8 | 17.9 | 17.9 |
|  |  | 95% CI | 17.7 - 18.4 | 17.4 - 18.0 | 17.6 - 18.3 | -- | -- | -- |
|  |  | Range | 17.1 - 20.5 | 17.2 - 18.5 | 17.1 - 18.7 | 17.0 - 19.0 | 16.9 - 19.3 | 16.7 - 18.8 |
|  | Male | Mean | 17.0 | 17.1 | 16.9 | 17.1 | 16.5 | 17.0 |
|  |  | 95% CI | 16.6 - 17.3 | 16.8 - 17.4 | 16.6 - 17.2 | -- | -- | -- |
|  |  | Range | 16.2 - 17.8 | 15.7 - 18.6 | 16.2 - 17.7 | 16.2 - 19.0 | 15.5 - 17.2 | 15.5 - 18.3 |
| **MCHC**  **(g/dL)** | Female | Mean | 32.7 | 32.4 | 32.3** | 32.3 | 32.8 | 32.3 |
|  |  | 95% CI | 32.4 - 33.0 | 32.2 - 32.7 | 32.1 - 32.6 | -- | -- | -- |
|  |  | Range | 31.4 - 35.3 | 31.6 - 33.1 | 31.5 - 33.5 | 31.3 - 33.4 | 31.7 - 35.2 | 31.8 - 33.3 |
|  | Male | Mean | 31.7 | 31.7 | 31.9 | 32.0 | 31.7 | 31.7 |
|  |  | 95% CI | 31.4 - 32.0 | 31.4 - 32.0 | 31.6 - 32.1 | -- | -- | -- |
|  |  | Range | 30.9 - 32.5 | 30.9 - 32.4 | 31.1 - 32.8 | 31.0 - 34.9 | 30.9 - 32.8 | 30.6 - 32.5 |
| **RDW**  **(%)** | Female | Mean | 11.5 | 11.6 | 11.5 | 11.6 | 11.6 | 11.5 |
|  |  | 95% CI | 11.3 - 11.7 | 11.5 - 11.8 | 11.3 - 11.6 | -- | -- | -- |
|  |  | Range | 10.8 - 12.1 | 11.2 - 12.2 | 10.7 - 12.0 | 11.0 - 12.3 | 10.9 - 12.5 | 10.8 - 12.5 |
|  | Male | Mean | 12.6 | 12.6 | 12.8 | 12.8 | 12.9 | 12.5 |
|  |  | 95% CI | 12.3 - 12.8 | 12.3 - 12.9 | 12.5 - 13.1 | -- | -- | -- |
|  |  | Range | 11.7 - 13.2 | 11.8 - 13.8 | 11.9 - 13.8 | 12.2 - 13.6 | 12.1 - 14.2 | 11.9 - 13.9 |
| **ARET**  **(×10^6^/μL)** | Female | Mean | 82.4 | 91.1 | 89.3 | 97.8 | 86.5 | 91.7 |
|  |  | 95% CI | 70.8 - 94.0 | 79.9 - 102.3 | 77.6 - 100.9 | -- | -- | -- |
|  |  | Range | 29.5 - 118.1 | 55.3 - 113.2 | 33.3 - 129.7 | 33.6 - 138.7 | 46.9 - 140.8 | 47.8 - 137.4 |
|  | Male | Mean | 105.9 | 101.2 | 100.9 | 95.7 | 99.4 | 101.8 |
|  |  | 95% CI | 94.7 - 117.1 | 90.0 - 112.4 | 89.7 - 112.1 | -- | -- | -- |
|  |  | Range | 60.2 - 143.1 | 71.3 - 158.5 | 61.4 - 166.9 | 55.7 - 146.2 | 70.9 - 133.3 | 66.7 - 125.4 |
| **PLT**  **(×10^3^/μL)** | Female | Mean | 1113 | 1076 | 1038 | 1125 | 1043 | 1118 |
|  |  | 95% CI | 1053 - 1173 | 1018 - 1135 | 978 - 1098 | -- | -- | -- |
|  |  | Range | 955 - 1272 | 886 - 1238 | 838 - 1278 | 935 - 1336 | 803 - 1274 | 885 - 2140 |
|  | Male | Mean | 1099 | 1073 | 1060 | 1110 | 1128 | 1040 |
|  |  | 95% CI | 1040 - 1157 | 1015 - 1132 | 1002 - 1118 | -- | -- | -- |
|  |  | Range | 801 - 1275 | 881 - 1275 | 908 - 1283 | 894 - 1465 | 866 - 1323 | 807 - 1297 |
| **WBC**  **(×10^3^/μL)** | Female | Mean | 6.97 | 6.07 | 5.82** | 5.81 | 6.66 | 6.53 |
|  |  | 95% CI | 6.10 - 7.84 | 5.23 - 6.91 | 4.96 - 6.69 | -- | -- | -- |
|  |  | Range | 3.36 - 9.24 | 3.27 - 9.50 | 2.69 - 8.92 | 3.70 - 8.52 | 4.71 - 9.62 | 4.13 - 8.99 |
|  | Male | Mean | 8.43 | 9.63 | 9.17 | 9.35 | 8.43 | 9.60 |
|  |  | 95% CI | 6.77 - 10.10 | 7.97 - 11.30 | 7.51 - 10.83 | -- | -- | -- |
|  |  | Range | 5.60 - 12.81 | 3.51 - 14.65 | 3.36 - 16.60 | 5.13 - 15.60 | 4.21 - 13.24 | 5.83 - 15.05 |

**Table S8. Hematology Summary of Rats fed Maize Source Diets (continued)**

|  | **Gender** | **Statistical Parameter** | **Control^a^** | **DP23211**  **High** | **DP23211**  **Low^a^** | **P0928^a^** | **P0993** | **P1105** |
| --- | --- | --- | --- | --- | --- | --- | --- | --- |
| **ANEU^#^**  **(×10^3^/μL)** | Female | Mean | 0.97 | 1.06 | 1.04 | 0.94 | 1.02 | 1.18 |
|  |  | 95% CI | 0.77 - 1.22 | 0.84 - 1.33 | 0.83 - 1.32 | -- | -- | -- |
|  |  | Range | 0.56 - 2.65 | 0.54 - 1.98 | 0.35 - 2.55 | 0.49 - 1.52 | 0.40 - 1.98 | 0.52 - 2.71 |
|  | Male | Mean | 1.47 | 1.90 | 1.77 | 1.73 | 1.74 | 2.33 |
|  |  | 95% CI | 1.17 - 1.84 | 1.51 - 2.38 | 1.41 - 2.22 | -- | -- | -- |
|  |  | Range | 0.78 - 2.36 | 0.79 - 3.79 | 0.80 - 4.60 | 0.71 - 3.72 | 0.98 - 4.10 | 1.16 - 9.78 |
| **ALYM**  **(×10^3^/μL)** | Female | Mean | 5.57 | 4.58** | 4.34** | 4.52 | 5.28 | 5.01 |
|  |  | 95% CI | 4.89 - 6.25 | 3.93 - 5.23 | 3.66 - 5.01 | -- | -- | -- |
|  |  | Range | 2.57 - 7.43 | 2.44 - 7.86 | 2.14 - 6.12 | 2.76 - 6.73 | 3.92 - 8.07 | 2.62 - 6.93 |
|  | Male | Mean | 6.48 | 7.05 | 6.73 | 7.12 | 6.28 | 6.68 |
|  |  | 95% CI | 5.01 - 7.94 | 5.58 - 8.51 | 5.27 - 8.20 | -- | -- | -- |
|  |  | Range | 4.07 - 10.44 | 2.56 - 12.50 | 2.42 - 13.00 | 4.13 - 11.56 | 3.07 - 9.78 | 4.18 - 10.41 |
| **AMON (×10^3^/μL)** | Female | Mean | 0.22 | 0.24 | 0.20 | 0.22 | 0.22 | 0.21 |
|  |  | 95% CI | 0.16 - 0.28 | 0.18 - 0.30 | 0.14 - 0.26 | -- | -- | -- |
|  |  | Range | 0.13 - 0.38 | 0.14 - 0.44 | 0.04 - 0.41 | 0.10 - 0.44 | 0.14 - 0.34 | 0.11 - 0.26 |
|  | Male | Mean | 0.25 | 0.29 | 0.30 | 0.31 | 0.23 | 0.40 |
|  |  | 95% CI | 0.19 - 0.32 | 0.23 - 0.35 | 0.23 - 0.36 | -- | -- | -- |
|  |  | Range | 0.06 - 0.45 | 0.09 - 0.43 | 0.05 - 0.58 | 0.16 - 0.61 | 0.08 - 0.44 | 0.13 - 0.82 |
| **AEOS^#^**  **(×10^3^/μL)** | Female | Mean | 0.06 | 0.07 | 0.06 | 0.07 | 0.08 | 0.08 |
|  |  | 95% CI | 0.05 - 0.07 | 0.06 - 0.09 | 0.05 - 0.08 | -- | -- | -- |
|  |  | Range | 0.04 - 0.09 | 0.03 - 0.14 | 0.02 - 0.16 | 0.03 - 0.13 | 0.05 - 0.14 | 0.02 - 0.13 |
|  | Male | Mean | 0.10 | 0.12 | 0.10 | 0.12 | 0.10 | 0.12 |
|  |  | 95% CI | 0.08 - 0.13 | 0.09 - 0.14 | 0.08 - 0.13 | -- | -- | -- |
|  |  | Range | 0.04 - 0.18 | 0.06 - 0.26 | 0.04 - 0.22 | 0.07 - 0.20 | 0.05 - 0.17 | 0.06 - 0.30 |
| **ABAS^#^ (×10^3^/μL)** | Female | Mean | 0.02 | 0.02 | 0.02 | 0.02 | 0.02 | 0.02 |
|  |  | 95% CI | 0.01 - 0.02 | 0.01 - 0.02 | 0.01 - 0.02 | -- | -- | -- |
|  |  | Range | 0.00 - 0.05 | 0.00 - 0.04 | 0.00 - 0.04 | 0.01 - 0.05 | 0.01 - 0.04 | 0.01 - 0.04 |
|  | Male | Mean | 0.02 | 0.03 | 0.02 | 0.03 | 0.03 | 0.03 |
|  |  | 95% CI | 0.02 - 0.03 | 0.02 - 0.04 | 0.02 - 0.03 | -- | -- | -- |
|  |  | Range | 0.01 - 0.04 | 0.00 - 0.07 | 0.00 - 0.07 | 0.01 - 0.08 | 0.02 - 0.05 | 0.00 - 0.05 |
| **ALUC^#^ (×10^3^/μL)** | Female | Mean | 0.04 | 0.04 | 0.04 | 0.04 | 0.05 | 0.04 |
|  |  | 95% CI | 0.03 - 0.05 | 0.03 - 0.04 | 0.04 - 0.05 | -- | -- | -- |
|  |  | Range | 0.02 - 0.07 | 0.01 - 0.10 | 0.01 - 0.11 | 0.01 - 0.07 | 0.02 - 0.10 | 0.02 - 0.10 |
|  | Male | Mean | 0.04 | 0.04 | 0.04 | 0.05 | 0.04 | 0.05^b^ |
|  |  | 95% CI | 0.02 - 0.05 | 0.03 - 0.06 | 0.03 - 0.06 | -- | -- | -- |
|  |  | Range | 0.01 - 0.09 | 0.01 - 0.07 | 0.01 - 0.14 | 0.01 - 0.11 | 0.01 - 0.08 | 0.00 - 0.10 |

^a^	n=15, females only; ^b^n=15; otherwise n=16

^#^	Test and control data were transformed prior to analysis. The means are presented on the data scale (back-transformed), confidence interval (CI) are presented on the transformed scale. Reference arithmetic means were calculated from the raw data.

**	Statistically significant difference when compared to control at the 0.05 level; FDR not significantly different.

**Table S9. Coagulation Summary of Rats fed Maize Source Diets**

|  | **Gender** | **Statistical Parameter** | **Control** | **DP23211**  **High** | **DP23211**  **Low** | **P0928** | **P0993** | **P1105** |
| --- | --- | --- | --- | --- | --- | --- | --- | --- |
| **PT**  **(sec)** | Female | Mean | 15.5^a^ | 15.6 | 15.5 | 15.9^b^ | 15.8 | 15.3^b^ |
|  |  | 95% CI | 15.1 - 15.8 | 15.2 - 15.9 | 15.1 - 15.8 | -- | -- | -- |
|  |  | Range | 14.3 - 16.1 | 14.8 - 16.8 | 14.3 - 16.8 | 14.7 - 19.6 | 14.8 - 17.4 | 13.9 - 16.6 |
|  | Male | Mean | 17.0 | 17.1 | 17.2 | 16.9 | 17.3 | 16.9 |
|  |  | 95% CI | 16.5 - 17.6 | 16.6 - 17.6 | 16.6 - 17.7 | -- | -- | -- |
|  |  | Range | 15.1 - 19.0 | 15.5 - 18.7 | 15.1 - 18.2 | 15.4 - 19.6 | 15.8 - 19.1 | 15.7 - 18.6 |
| **APTT**  **(sec)** | Female | Mean | 14.5^a^ | 14.7 | 14.1 | 14.0^b^ | 14.2 | 13.9^c^ |
|  |  | 95% CI | 13.7 - 15.2 | 14.0 - 15.5 | 13.4 - 14.9 | -- | -- | -- |
|  |  | Range | 12.6 - 17.0 | 12.0 - 17.4 | 11.8 - 17.2 | 11.4 - 17.2 | 12.0 - 15.8 | 11.8 - 15.8 |
|  | Male | Mean | 15.5 | 15.7 | 16.0 | 15.6 | 15.8 | 15.6 |
|  |  | 95% CI | 14.8 - 16.3 | 15.0 - 16.5 | 15.3 - 16.7 | -- | -- | -- |
|  |  | Range | 13.3 - 18.2 | 12.7 - 17.6 | 13.3 - 18.1 | 12.4 - 19.8 | 12.3 - 17.9 | 12.1 - 17.9 |

Note: Test and control means are the LS-Means from the linear mixed model. Reference means are the arithmetic means. When a reported statistic was estimated to be < 10^-10^ in absolute value, the value was set to zero.

^a^	n=15; ^b^ n=14; ^c^ n=13; otherwise n=16

**Table S10. Clinical Chemistry Summary of Rats fed Maize Source Diets**

|  | **Gender** | **Statistical Parameter** | **Control** | **DP23211**  **High** | **DP23211**  **Low** | **P0928** | **P0993** | **P1105** |
| --- | --- | --- | --- | --- | --- | --- | --- | --- |
| **AST^#^**  **(U/L)** | Female | Mean | 92^a^ | 89 | 88^a^ | 95^b^ | 98 | 86 |
|  |  | 95% CI | 80 - 109 | 77 - 103 | 77 - 102 | -- | -- | -- |
|  |  | Range | 59 - 145 | 68 - 179 | 66 - 188 | 70 - 147 | 62 - 137 | 65 - 122 |
|  | Male | Mean | 91 | 95 | 85 | 112^a^ | 89^a^ | 88 |
|  |  | 95% CI | 79 - 106 | 83 - 112 | 75 - 98 | -- | -- | -- |
|  |  | Range | 62 - 141 | 68 - 284 | 63 - 160 | 60 - 387 | 71 - 138 | 67 - 118 |
| **ALT^#^**  **(U/L)** | Female | Mean | 28^a^ | 24** | 26 | 24^a^ | 31 | 26 |
|  |  | 95% CI | 25 - 33 | 21 - 27 | 23 - 30 | -- | -- | -- |
|  |  | Range | 20 - 57 | 17 - 67 | 16 - 43 | 18 - 36 | 17 - 62 | 17 - 52 |
|  | Male | Mean | 29 | 33 | 31 | 36 | 33 | 27 |
|  |  | 95% CI | 26 - 35 | 28 - 40 | 27 - 37 | -- | -- | -- |
|  |  | Range | 19 - 53 | 26 - 56 | 25 - 99 | 21 - 118 | 24 - 70 | 21 - 36 |
| **SDH^#^**  **(U/L)** | Female | Mean | 14.0^a^ | 13.3 | 12.8^a^ | 13.7^b^ | 16.2 | 14.5 |
|  |  | 95% CI | 10.8 - 18.0 | 10.4 - 17.0 | 9.9 - 16.5 | -- | -- | -- |
|  |  | Range | 5.5 - 49.1 | 6.6 - 36.6 | 9.1 - 20.1 | 8.6 - 20.6 | 6.0 - 28.3 | 9.2 - 23.9 |
|  | Male | Mean | 14.4 | 15.5 | 15.7 | 20.1^a^ | 16.9^a^ | 13.1 |
|  |  | 95% CI | 11.2 - 18.4 | 12.1 - 19.8 | 12.2 - 20.1 | -- | -- | -- |
|  |  | Range | 8.6 - 24.9 | 7.8 - 35.9 | 9.3 - 59.9 | 9.7 - 83.6 | 9.3 - 43.7 | 9.0 - 21.2 |
| **ALKP**  **(U/L)** | Female | Mean | 46^a^ | 49 | 56**! | 54^a^ | 54 | 50 |
|  |  | 95% CI | 36 - 55 | 40 - 58 | 47 - 65 | -- | -- | -- |
|  |  | Range | 31 - 79 | 29 - 73 | 31 - 83 | 29 - 106 | 25 - 108 | 24 - 84 |
|  | Male | Mean | 81 | 83 | 91! | 82 | 85 | 76 |
|  |  | 95% CI | 70 - 92 | 72 - 93 | 80 - 102 | -- | -- | -- |
|  |  | Range | 57 - 108 | 52 - 127 | 58 - 135 | 51 - 149 | 52 - 150 | 54 - 128 |
| **BILI**  **(mg/dL)** | Female | Mean | 0.20^a^ | 0.20 | 0.21 | 0.20^a^ | 0.20 | 0.21 |
|  |  | 95% CI | 0.18 - 0.22 | 0.18 - 0.22 | 0.19 - 0.23 | -- | -- | -- |
|  |  | Range | 0.16 - 0.24 | 0.16 - 0.25 | 0.14 - 0.28 | 0.16 - 0.34 | 0.16 - 0.31 | 0.15 - 0.28 |
|  | Male | Mean | 0.19 | 0.18 | 0.19 | 0.19 | 0.19 | 0.17 |
|  |  | 95% CI | 0.17 - 0.21 | 0.16 - 0.20 | 0.17 - 0.21 | -- | -- | -- |
|  |  | Range | 0.17 - 0.23 | 0.12 - 0.27 | 0.14 - 0.29 | 0.14 - 0.42 | 0.14 - 0.29 | 0.14 - 0.22 |
| **BUN**  **(mg/dL)** | Female | Mean | 16^a^ | 17 | 18** | 16^a^ | 18 | 17 |
|  |  | 95% CI | 14 - 18 | 15 - 19 | 16 - 20 | -- | -- | -- |
|  |  | Range | 12 - 23 | 11 - 24 | 13 - 25 | 12 - 19 | 12 - 27 | 13 - 21 |
|  | Male | Mean | 16 | 17 | 16 | 16 | 16 | 15 |
|  |  | 95% CI | 14 - 18 | 15 - 19 | 14 - 18 | -- | -- | -- |
|  |  | Range | 13 - 18 | 14 - 20 | 11 - 19 | 11 - 19 | 14 - 21 | 12 - 22 |
| **CREA**  **(mg/dL)** | Female | Mean | 0.34^a^ | 0.37 | 0.38** | 0.38^a^ | 0.37 | 0.38 |
|  |  | 95% CI | 0.31 - 0.37 | 0.34 - 0.40 | 0.35 - 0.41 | -- | -- | -- |
|  |  | Range | 0.30 - 0.44 | 0.30 - 0.46 | 0.28 - 0.50 | 0.32 - 0.50 | 0.31 - 0.47 | 0.28 - 0.45 |
|  | Male | Mean | 0.32 | 0.33 | 0.32 | 0.33 | 0.33 | 0.34 |
|  |  | 95% CI | 0.29 - 0.35 | 0.31 - 0.36 | 0.29 - 0.35 | -- | -- | -- |
|  |  | Range | 0.25 - 0.40 | 0.27 - 0.39 | 0.19 - 0.43 | 0.28 - 0.39 | 0.23 - 0.47 | 0.23 - 0.43 |
| **CHOL^#^**  **(mg/dL)** | Female | Mean | 65.94^a^ | 61.55 | 61.14 | 60.47^a^ | 64.88 | 69.25 |
|  |  | 95% CI | 58.54 - 74.28 | 54.84 - 69.07 | 54.48 - 68.62 | -- | -- | -- |
|  |  | Range | 47.00 - 85.00 | 43.00 - 109.00 | 42.00 - 96.00 | 32.00 - 86.00 | 46.00 - 80.00 | 24.00 - 99.00 |
|  | Male | Mean | 48.71 | 47.69 | 50.07 | 51.63 | 49.06 | 47.44 |
|  |  | 95% CI | 43.41 - 54.67 | 42.49 - 53.52 | 44.62 - 56.19 | -- | -- | -- |
|  |  | Range | 36.00 - 72.00 | 28.00 - 69.00 | 33.00 - 73.00 | 27.00 - 95.00 | 32.00 - 77.00 | 35.00 - 66.00 |
| **HDL**  **(mg/dL)** | Female | Mean | 45.8^a^ | 42.9 | 42.3 | 42.0^a^ | 43.5 | 46.8 |
|  |  | 95% CI | 41.8 - 49.8 | 39.1 - 46.8 | 38.4 - 46.2 | -- | -- | -- |
|  |  | Range | 33.3 - 57.9 | 32.3 - 68.7 | 30.9 - 60.9 | 25.2 - 57.8 | 33.2 - 52.3 | 17.2 - 62.5 |
|  | Male | Mean | 30.8 | 30.6 | 31.9 | 31.8 | 31.2 | 29.0 |
|  |  | 95% CI | 26.9 - 34.7 | 26.7 - 34.4 | 28.0 - 35.8 | -- | -- | -- |
|  |  | Range | 21.3 - 41.4 | 19.8 - 39.8 | 23.0 - 45.0 | 18.8 - 50.1 | 20.6 - 47.9 | 18.9 - 38.5 |
| **nHDL**  **(mg/dL)** | Female | Mean | 21.3^a^ | 20.6 | 19.8 | 18.5^a^ | 21.4 | 22.4 |
|  |  | 95% CI | 18.2 - 24.4 | 17.5 - 23.6 | 16.8 - 22.9 | -- | -- | -- |
|  |  | Range | 13.0 - 29.1 | 10.7 - 40.3 | 11.1 - 35.1 | 6.8 - 30.6 | 12.8 - 30.6 | 6.8 - 37.0 |
|  | Male | Mean | 19.1 | 18.4 | 19.8 | 19.9 | 17.9 | 18.4 |
|  |  | 95% CI | 16.0 - 22.1 | 15.4 - 21.5 | 16.8 - 22.8 | -- | -- | -- |
|  |  | Range | 12.5 - 30.6 | 8.2 - 29.2 | 9.9 - 31.4 | 8.2 - 44.9 | 10.0 - 29.1 | 10.6 - 27.7 |

**Table S10. Clinical Chemistry Summary of Rats fed Maize Source Diets (continued)**

|  | **Gender** | **Statistical Parameter** | **Control** | **DP23211**  **High** | **DP23211**  **Low** | **P0928** | **P0993** | **P1105** |
| --- | --- | --- | --- | --- | --- | --- | --- | --- |
| **TRIG^#*^**  **(mg/dL)** | Female | Mean | 34^a^ | 37! | 39 | 42^a^ | 37 | 43 |
|  |  | 95% CI | 30 - 39 | 33 - 42 | 34 - 44 | -- | -- | -- |
|  |  | Range | 27 - 44 | 23 - 51 | 24 - 87 | 25 - 82 | 25 - 61 | 24 - 59 |
|  | Male | Mean | 69 | 46**! | 56 | 53 | 49 | 44 |
|  |  | 95% CI | 58 - 82 | 38 - 55 | 47 - 66 | -- | -- | -- |
|  |  | Range | 35 - 130 | 29 - 79 | 30 - 91 | 35 - 73 | 20 - 88 | 24 - 66 |
| **GLUC**  **(mg/dL)** | Female | Mean | 152^a^ | 149 | 147 | 146^a^ | 154 | 154 |
|  |  | 95% CI | 134 - 171 | 131 - 167 | 129 - 165 | -- | -- | -- |
|  |  | Range | 104 - 205 | 108 - 224 | 96 - 197 | 99 - 202 | 102 - 218 | 92 - 205 |
|  | Male | Mean | 179 | 185 | 187 | 183 | 192 | 169 |
|  |  | 95% CI | 161 - 197 | 167 - 203 | 169 - 205 | -- | -- | -- |
|  |  | Range | 138 - 218 | 120 - 240 | 120 - 235 | 117 - 242 | 137 - 226 | 115 - 224 |
| **TP**  **(g/dL)** | Female | Mean | 6.7^a^ | 6.9 | 6.7 | 6.8^a^ | 6.7 | 6.8 |
|  |  | 95% CI | 6.5 - 6.9 | 6.7 - 7.1 | 6.5 - 6.9 | -- | -- | -- |
|  |  | Range | 5.9 - 7.7 | 6.2 - 7.5 | 5.9 - 7.7 | 6.4 - 7.3 | 6.0 - 7.4 | 6.2 - 7.2 |
|  | Male | Mean | 6.2 | 6.1 | 6.2 | 6.1 | 6.3 | 6.0 |
|  |  | 95% CI | 6.0 - 6.3 | 6.0 - 6.2 | 6.1 - 6.3 | -- | -- | -- |
|  |  | Range | 5.8 - 6.6 | 5.8 - 6.4 | 5.8 - 6.6 | 5.7 - 6.4 | 5.8 - 6.7 | 5.6 - 6.3 |
| **ALB**  **(g/dL)** | Female | Mean | 3.5^a^ | 3.6 | 3.5 | 3.5^a^ | 3.4 | 3.6 |
|  |  | 95% CI | 3.4 - 3.6 | 3.5 - 3.7 | 3.4 - 3.6 | -- | -- | -- |
|  |  | Range | 3.1 - 4.0 | 3.2 - 4.0 | 3.0 - 4.1 | 3.2 - 3.7 | 2.9 - 3.7 | 3.3 - 3.8 |
|  | Male | Mean | 3.0 | 3.0 | 3.0 | 2.9 | 3.0 | 2.9 |
|  |  | 95% CI | 2.9 - 3.0 | 2.9 - 3.0 | 2.9 - 3.0 | -- | -- | -- |
|  |  | Range | 2.7 - 3.2 | 2.8 - 3.1 | 2.8 - 3.2 | 2.7 - 3.1 | 2.9 - 3.2 | 2.5 - 3.2 |
| **GLOB**  **(g/dL)** | Female | Mean | 3.2^a^ | 3.3 | 3.2 | 3.3^a^ | 3.3 | 3.2 |
|  |  | 95% CI | 3.1 - 3.3 | 3.2 - 3.4 | 3.1 - 3.3 | -- | -- | -- |
|  |  | Range | 2.8 - 3.7 | 3.0 - 3.7 | 2.8 - 3.6 | 3.1 - 3.6 | 3.0 - 3.7 | 2.9 - 3.4 |
|  | Male | Mean | 3.2 | 3.1 | 3.2 | 3.1 | 3.3 | 3.1 |
|  |  | 95% CI | 3.1 - 3.3 | 3.0 - 3.2 | 3.1 - 3.3 | -- | -- | -- |
|  |  | Range | 2.9 - 3.6 | 2.9 - 3.4 | 3.0 - 3.5 | 2.8 - 3.4 | 2.9 - 3.6 | 2.9 - 3.3 |
| **CALC**  **(mg/dL)** | Female | Mean | 10.9^a^ | 11.0 | 10.9 | 11.1^a^ | 10.8 | 10.9 |
|  |  | 95% CI | 10.7 - 11.1 | 10.8 - 11.2 | 10.7 - 11.1 | -- | -- | -- |
|  |  | Range | 10.1 - 11.9 | 10.5 - 11.3 | 9.6 - 11.8 | 10.5 - 11.7 | 10.1 - 11.5 | 10.4 - 11.5 |
|  | Male | Mean | 10.4 | 10.5 | 10.5 | 10.4 | 10.5 | 10.5 |
|  |  | 95% CI | 10.1 - 10.6 | 10.2 - 10.7 | 10.2 - 10.7 | -- | -- | -- |
|  |  | Range | 9.6 - 11.1 | 9.7 - 11.0 | 9.7 - 11.4 | 9.9 - 11.3 | 9.8 - 11.4 | 9.9 - 11.2 |
| **IPHS**  **(mg/dL)** | Female | Mean | 8.1^a^ | 8.5 | 8.2 | 8.6^a^ | 8.1 | 8.0 |
|  |  | 95% CI | 7.5 - 8.6 | 8.0 - 8.9 | 7.7 - 8.7 | -- | -- | -- |
|  |  | Range | 6.5 - 9.0 | 6.6 - 9.8 | 4.5 - 10.6 | 7.6 - 9.3 | 6.3 - 9.0 | 6.7 - 8.8 |
|  | Male | Mean | 7.9 | 8.2 | 8.0 | 7.9 | 8.0 | 8.3 |
|  |  | 95% CI | 7.4 - 8.4 | 7.7 - 8.7 | 7.5 - 8.5 | -- | -- | -- |
|  |  | Range | 6.2 - 9.1 | 6.8 - 9.7 | 6.8 - 9.7 | 6.5 - 9.6 | 6.8 - 9.1 | 6.7 - 9.6 |
| **NA**  **(mmol/L)** | Female | Mean | 143.4^a^ | 142.8 | 143.0 | 142.8^a^ | 142.5 | 142.8 |
|  |  | 95% CI | 142.8 - 144.0 | 142.2 - 143.4 | 142.4 - 143.6 | -- | -- | -- |
|  |  | Range | 142.5 - 145.2 | 142.0 - 143.5 | 140.3 - 145.3 | 141.3 - 144.0 | 140.0 - 144.7 | 140.2 - 144.4 |
|  | Male | Mean | 144.3 | 144.5 | 144.5 | 144.2 | 144.4 | 144.5 |
|  |  | 95% CI | 143.7 - 144.8 | 143.9 - 145.0 | 144.0 - 145.1 | -- | -- | -- |
|  |  | Range | 141.0 - 146.3 | 143.2 - 145.3 | 143.2 - 146.3 | 143.1 - 146.3 | 143.2 - 146.0 | 142.1 - 146.1 |
| **K**  **(mmol/L)** | Female | Mean | 5.58^a^ | 5.83 | 6.07^a^ | 6.47^b^ | 5.42 | 5.97 |
|  |  | 95% CI | 5.18 - 5.98 | 5.44 - 6.21 | 5.67 - 6.47 | -- | -- | -- |
|  |  | Range | 4.27 - 6.69 | 4.81 - 7.31 | 4.93 - 8.05 | 5.41 - 7.95 | 3.96 - 6.73 | 4.07 - 9.00 |
|  | Male | Mean | 6.24 | 6.24 | 6.38 | 6.00^a^ | 6.25^a^ | 6.05 |
|  |  | 95% CI | 5.85 - 6.62 | 5.85 - 6.62 | 6.00 - 6.77 | -- | -- | -- |
|  |  | Range | 4.66 - 8.14 | 5.36 - 7.36 | 4.78 - 7.71 | 4.55 - 6.62 | 4.38 - 7.28 | 4.68 - 6.84 |
| **CL**  **(mmol/L)** | Female | Mean | 103.8^a^ | 103.8 | 104.4 | 103.8^a^ | 103.5 | 103.5 |
|  |  | 95% CI | 102.8 - 104.7 | 102.9 - 104.7 | 103.4 - 105.3 | -- | -- | -- |
|  |  | Range | 100.9 - 106.0 | 100.9 - 106.0 | 100.5 - 108.1 | 100.2 - 106.0 | 101.3 - 105.3 | 101.2 - 106.8 |
|  | Male | Mean | 104.4 | 104.3 | 104.0 | 104.5 | 104.1 | 104.4 |
|  |  | 95% CI | 103.5 - 105.3 | 103.5 - 105.2 | 103.2 - 104.9 | -- | -- | -- |
|  |  | Range | 102.3 - 107.7 | 101.4 - 107.4 | 102.7 - 105.4 | 101.3 - 106.1 | 101.6 - 106.8 | 102.0 - 108.9 |

**Table S10. Clinical Chemistry Summary of Rats fed Maize Source Diets (continued)**

|  | **Gender** | **Statistical Parameter** | **Control** | **DP23211**  **High** | **DP23211**  **Low** | **P0928** | **P0993** | **P1105** |
| --- | --- | --- | --- | --- | --- | --- | --- | --- |
| **TBA^#^**  **(µmol/L)** | Female | Mean | 12.8^a^ | 13.5 | 12.8 | 13.7^a^ | 14.8 | 14.0 |
|  |  | 95% CI | 10.0 - 16.5 | 10.6 - 17.2 | 10.1 - 16.4 | -- | -- | -- |
|  |  | Range | 4.4 - 29.9 | 5.8 - 42.0 | 8.9 - 17.4 | 7.5 - 26.8 | 6.1 - 32.1 | 7.3 - 30.2 |
|  | Male | Mean | 10.9 | 11.7 | 9.2 | 17.8 | 15.0 | 13.5 |
|  |  | 95% CI | 8.6 - 13.9 | 9.2 - 14.9 | 7.2 - 11.8 | -- | -- | -- |
|  |  | Range | 4.7 - 31.7 | 5.2 - 32.9 | 3.8 - 28.0 | 4.7 - 93.7 | 5.1 - 38.1 | 5.6 - 25.5 |
| **T3^#^**  **(ng/mL)** | Female | Mean | 0.497^a^ | 0.552 | 0.473 | 0.533^a^ | 0.507 | 0.443 |
|  |  | 95% CI | 0.449 - 0.554 | 0.496 - 0.618 | 0.428 - 0.525 | -- | -- | -- |
|  |  | Range | 0.314 - 0.703 | 0.430 - 1.120 | 0.328 - 0.634 | 0.356 - 1.450 | 0.426 - 0.627 | 0.326 - 0.581 |
|  | Male | Mean | 0.353 | 0.372 | 0.352 | 0.373 | 0.366^a^ | 0.359 |
|  |  | 95% CI | 0.324 - 0.387 | 0.340 - 0.408 | 0.323 - 0.386 | -- | -- | -- |
|  |  | Range | 0.182 - 0.483 | 0.313 - 0.556 | 0.274 - 0.486 | 0.294 - 0.518 | 0.284 - 0.494 | 0.249 - 0.551 |
| **T4^#^**  **(ug/mL)** | Female | Mean | 43.0^a^ | 44.3 | 40.6 | 48.2^a^ | 41.7 | 37.0 |
|  |  | 95% CI | 37.5 - 49.2 | 38.7 - 50.8 | 35.5 - 46.5 | -- | -- | -- |
|  |  | Range | 25.2 - 57.6 | 24.1 - 87.4 | 32.0 - 58.3 | 32.8 - 88.1 | 22.0 - 57.2 | 24.5 - 56.7 |
|  | Male | Mean | 63.5 | 65.0 | 66.6 | 63.7 | 62.8^a^ | 59.7 |
|  |  | 95% CI | 55.4 - 72.6 | 56.8 - 74.4 | 58.1 - 76.2 | -- | -- | -- |
|  |  | Range | 32.0 - 120.0 | 46.8 - 99.0 | 54.2 - 120.0 | 43.0 - 85.6 | 45.5 - 84.9 | 32.2 - 83.9 |
| **TSH**  **(ng/mL)** | Female | Mean | 3.59^a^ | 3.60 | 3.73 | 3.50^a^ | 3.40 | 3.66^a^ |
|  |  | 95% CI | 3.44 - 4.14 | 3.05 - 4.15 | 3.18 - 4.27 | -- | -- | -- |
|  |  | Range | 2.34 - 5.52 | 1.10 - 5.04 | 2.29 - 6.37 | 2.53 - 4.56 | 2.37 - 5.38 | 2.74 - 5.47 |
|  | Male | Mean | 4.36 | 4.72 | 4.30 | 4.73 | 5.38 | 4.19^a^ |
|  |  | 95% CI | 3.81 - 4.91 | 4.17 - 5.26 | 3.75 - 4.85 | -- | -- | -- |
|  |  | Range | 3.33 - 5.61 | 3.27 - 7.89 | 3.07 - 6.04 | 2.98 - 7.62 | 3.29 - 9.68 | 3.04 - 6.32 |

Note: When a reported statistic was estimated to be < 10^-10^ in absolute value, the value was set to zero.

^a^	n=15; ^b^ n=13; otherwise n=16

^#^	Test and control data were transformed prior to analysis. The means are presented on the data scale (back-transformed), confidence interval (CI) are presented on the transformed scale. Reference arithmetic means were calculated from the raw data.

*	Sex x Treatment interaction statistically significant at the 0.05 level

**	Statistically significant difference when compared to control at the 0.05 level; FDR not significantly different

!	Combined statistically significant difference when compared to combined control at the 0.05 level; combined FDR not significantly different

**Table S11. Urinalysis Summary of Rats fed Maize Source Diets**

|  | **Gender** | **Statistical Parameter** | **Control** | **DP23211**  **High** | **DP23211**  **Low** | **P0928**^a^ | **P0993** | **P1105** |
| --- | --- | --- | --- | --- | --- | --- | --- | --- |
| **UVOL^#^**  **(mL)** | Female | Mean | 5.2 | 5.7 | 6.3 | 7.2 | 7.7 | 8.1 |
|  |  | 95% CI | 3.3 - 8.0 | 3.7 - 8.8 | 4.1 - 9.7 | -- | -- | -- |
|  |  | Range | 0.3 - 44.0 | 3.0 - 12.8 | 3.0 - 24.0 | 0.5 - 23.2 | 0.2 - 21.2 | 2.0 - 23.0 |
|  | Male | Mean | 8.5 | 8.9 | 7.8 | 11.0 | 15.9 | 11.4 |
|  |  | 95% CI | 6.6 - 10.9 | 6.9 - 11.4 | 6.1 - 9.9 | -- | -- | -- |
|  |  | Range | 3.6 - 18.4 | 3.6 - 19.0 | 4.6 - 17.8 | 4.4 - 28.0 | 4.8 - 30.0 | 3.8 - 20.0 |
| **SG** | Female | Mean | 1.040 | 1.036 | 1.037 | 1.037 | 1.034 | 1.033 |
|  |  | 95% CI | 1.032 - 1.049 | 1.028 - 1.045 | 1.028 - 1.045 | -- | -- | -- |
|  |  | Range | 1.006 - 1.100 | 1.017 - 1.059 | 1.013 - 1.061 | 1.013 - 1.100 | 1.016 - 1.100 | 1.011 - 1.069 |
|  | Male | Mean | 1.043 | 1.040 | 1.045 | 1.037 | 1.028 | 1.035 |
|  |  | 95% CI | 1.035 - 1.051 | 1.032 - 1.049 | 1.037 - 1.054 | -- | -- | -- |
|  |  | Range | 1.022 - 1.076 | 1.019 - 1.061 | 1.025 - 1.062 | 1.020 - 1.058 | 1.011 - 1.059 | 1.017 - 1.063 |
| **pH** | Female | Mean | 6.8 | 6.8 | 6.9 | 6.8 | 6.8 | 6.6 |
|  |  | 95% CI | 6.5 - 7.0 | 6.5 - 7.0 | 6.6 - 7.1 | -- | -- | -- |
|  |  | Range | 6.0 - 8.0 | 6.0 - 8.0 | 6.5 - 7.5 | 6.0 - 7.5 | 6.0 - 7.5 | 6.0 - 7.5 |
|  | Male | Mean | 7.2 | 7.3 | 7.1 | 7.3 | 7.5 | 7.2 |
|  |  | 95% CI | 6.9 - 7.4 | 7.0 - 7.5 | 6.8 - 7.4 | -- | -- | -- |
|  |  | Range | 6.5 - 8.0 | 7.0 - 8.5 | 6.5 - 8.0 | 6.5 - 8.5 | 7.0 - 8.5 | 6.5 - 8.5 |
| **UMTP^#^ (mg/dL)** | Female | Mean | 36 | 35 | 33 | 37 | 38 | 33 |
|  |  | 95% CI | 25 - 51 | 24 - 50 | 23 - 47 | -- | -- | -- |
|  |  | Range | 6 - 140 | 17 - 83 | 11 - 83 | 10 - 123 | 15 - 164 | 8 - 73 |
|  | Male | Mean | 156 | 140 | 165 | 139 | 101 | 145 |
|  |  | 95% CI | 133 - 182 | 119 - 164 | 141 - 194 | -- | -- | -- |
|  |  | Range | 63 - 428 | 64 - 274 | 106 - 300 | 69 - 186 | 38 - 207 | 65 - 288 |

^a^	n=15; females only; otherwise n=16

^#^	Test and control data were transformed prior to analysis. The means are presented on the data scale (back-transformed), confidence interval (CI) are presented on the transformed scale. Reference arithmetic means were calculated from the raw data.

**Table S12. Organ Weight, Organ to Terminal Body Weight, and Organ to Brain Weight Results for Rats fed Maize Source Diets**

|  | **Sex** | **Statistical Parameter** | **Control** | **DP23211**  **High** | **DP23211**  **Low** | **P0928^a^** | **P0993** | **P1105** |
| --- | --- | --- | --- | --- | --- | --- | --- | --- |
| **Terminal**  **Body Wt**  **(g)** | Female | Mean | 292.8 | 278.3 | 289.4 | 284.5 | 283.6 | 290.7 |
|  |  | 95% CI | 274.8 - 310.7 | 260.3 - 296.3 | 271.4 - 307.4 | -- | -- | -- |
|  |  | Range | 257.6 - 340.5 | 245.6 - 312.0 | 242.0 - 343.6 | 251.7 - 316.0 | 239.7 - 336.1 | 247.8 - 350.6 |
|  | Male | Mean | 587.2 | 574.3 | 597.1 | 556.3 | 571.6 | 568.2 |
|  |  | 95% CI | 558.4 - 616.1 | 545.5 - 603.2 | 568.2 - 625.9 | -- | -- | -- |
|  |  | Range | 493.8 - 724.0 | 495.1 - 633.7 | 515.0 - 744.6 | 495.1 - 655.8 | 489.4 - 639.8 | 446.8 - 636.1 |
| **Adrenal Glands**  **(g)** | Female | Mean | 0.070 | 0.075 | 0.075 | 0.070 | 0.070^b^ | 0.071 |
|  |  | 95% CI | 0.065 - 0.074 | 0.070 - 0.079 | 0.071 - 0.080 | -- | -- | -- |
|  |  | Range | 0.050 - 0.085 | 0.054 - 0.087 | 0.053 - 0.101 | 0.050 - 0.089 | 0.046 - 0.090 | 0.054 - 0.088 |
|  | Male | Mean | 0.062 | 0.060 | 0.062 | 0.058 | 0.058 | 0.067 |
|  |  | 95% CI | 0.058 - 0.067 | 0.055 - 0.065 | 0.057 - 0.067 | -- | -- | -- |
|  |  | Range | 0.053 - 0.080 | 0.045 - 0.075 | 0.047 - 0.079 | 0.042 - 0.077 | 0.044 - 0.074 | 0.044 - 0.089 |
| **Adrenal Glands / Brain Wt**  **(%)** | Female | Mean | 3.365 | 3.666 | 3.685 | 3.438 | 3.257^b^ | 3.512 |
|  |  | 95% CI | 3.116 - 3.614 | 3.417 - 3.914 | 3.436 - 3.934 | -- | -- | -- |
|  |  | Range | 2.413 - 4.140 | 2.832 - 4.199 | 2.519 - 5.073 | 2.449 - 4.376 | 2.101 - 4.274 | 2.582 - 4.581 |
|  | Male | Mean | 2.770 | 2.661 | 2.686 | 2.581 | 2.565 | 2.957 |
|  |  | 95% CI | 2.521 - 3.019 | 2.413 - 2.910 | 2.437 - 2.935 | -- | -- | -- |
|  |  | Range | 2.286 - 3.518 | 1.875 - 3.595 | 1.930 - 3.284 | 1.814 - 3.229 | 1.967 - 3.197 | 2.086 - 4.051 |
| **Adrenal Glands / Term BW**  **(%)** | Female | Mean | 0.024 | 0.027 | 0.026 | 0.025 | 0.025^b^ | 0.025 |
|  |  | 95% CI | 0.021 - 0.026 | 0.025 - 0.029 | 0.024 - 0.029 | -- | -- | -- |
|  |  | Range | 0.017 - 0.030 | 0.021 - 0.031 | 0.015 - 0.038 | 0.017 - 0.034 | 0.016 - 0.035 | 0.017 - 0.035 |
|  | Male | Mean | 0.011 | 0.010 | 0.010 | 0.011 | 0.010 | 0.012 |
|  |  | 95% CI | 0.010 - 0.012 | 0.010 - 0.011 | 0.009 - 0.011 | -- | -- | -- |
|  |  | Range | 0.008 - 0.016 | 0.007 - 0.013 | 0.008 - 0.013 | 0.007 - 0.014 | 0.008 - 0.013 | 0.008 - 0.015 |
| **Accessory Sex Organs**  **(g)** | Male | Mean | 3.579 | 3.526 | 3.662 | 3.430 | 3.573 | 3.516 |
|  |  | 95% CI | 3.396 - 3.763 | 3.342 - 3.709 | 3.479 - 3.846 | -- | -- | -- |
|  |  | Range | 2.685 - 4.299 | 2.608 - 4.772 | 3.079 - 4.253 | 2.866 - 3.955 | 2.688 - 4.264 | 2.724 - 4.347 |
| **Accessory Sex Organs / Brain Wt (%)** | Male | Mean | 159.717 | 156.429 | 159.407 | 151.935 | 157.484 | 155.878 |
|  |  | 95% CI | 152.068 - 167.366 | 148.779 - 164.078 | 151.758 - 167.057 | -- | -- | -- |
|  |  | Range | 114.842 - 190.446 | 118.545 - 218.598 | 129.328 - 191.318 | 130.218 - 175.934 | 121.136 - 178.673 | 123.650 - 204.180 |
| **Accessory Sex Organs / Term BW (%)** | Male | Mean | 0.615 | 0.618 | 0.622 | 0.622 | 0.627 | 0.622 |
|  |  | 95% CI | 0.570 - 0.660 | 0.572 - 0.663 | 0.577 - 0.667 | -- | -- | -- |
|  |  | Range | 0.444 - 0.769 | 0.481 - 0.867 | 0.429 - 0.745 | 0.507 - 0.783 | 0.470 - 0.712 | 0.516 - 0.812 |
| **Brain**  **(g)** | Female | Mean | 2.071 | 2.041 | 2.056 | 2.044 | 2.099^b^ | 2.023 |
|  |  | 95% CI | 2.021 - 2.121 | 1.991 - 2.091 | 2.006 - 2.105 | -- | -- | -- |
|  |  | Range | 1.969 - 2.200 | 1.907 - 2.269 | 1.924 - 2.198 | 1.934 - 2.207 | 1.996 - 2.255 | 1.805 - 2.219 |
|  | Male | Mean | 2.246 | 2.255 | 2.303 | 2.260 | 2.268 | 2.259 |
|  |  | 95% CI | 2.196 - 2.296 | 2.206 - 2.305 | 2.254 - 2.353 | -- | -- | -- |
|  |  | Range | 1.997 - 2.440 | 1.999 - 2.400 | 2.118 - 2.472 | 2.089 - 2.482 | 2.175 - 2.410 | 2.109 - 2.353 |
| **Brain /**  **Term BW**  **(%)** | Female | Mean | 0.710 | 0.737 | 0.716 | 0.723 | 0.743^b^ | 0.700 |
|  |  | 95% CI | 0.675 - 0.745 | 0.702 - 0.772 | 0.681 - 0.751 | -- | -- | -- |
|  |  | Range | 0.623 - 0.805 | 0.646 - 0.859 | 0.612 - 0.870 | 0.630 - 0.877 | 0.599 - 0.896 | 0.585 - 0.803 |
|  | Male | Mean | 0.386 | 0.394 | 0.389 | 0.409 | 0.398 | 0.400 |
|  |  | 95% CI | 0.364 - 0.407 | 0.373 - 0.416 | 0.367 - 0.411 | -- | -- | -- |
|  |  | Range | 0.309 - 0.448 | 0.317 - 0.468 | 0.332 - 0.448 | 0.355 - 0.445 | 0.362 - 0.451 | 0.353 - 0.493 |
| **Epididymides**  **(g)** | Male | Mean | 1.423 | 1.481 | 1.514 | 1.482 | 1.408 | 1.471 |
|  |  | 95% CI | 1.357 - 1.489 | 1.415 - 1.547 | 1.448 - 1.580 | -- | -- | -- |
|  |  | Range | 1.078 - 1.655 | 1.275 - 1.900 | 1.262 - 1.777 | 1.305 - 1.848 | 1.156 - 1.619 | 1.116 - 1.729 |
| **Epididymides / Brain Wt (%)** | Male | Mean | 63.423 | 65.662 | 65.827 | 65.615 | 62.099 | 65.191 |
|  |  | 95% CI | 60.943 - 65.904 | 63.182 - 68.142 | 63.347 - 68.307 | -- | -- | -- |
|  |  | Range | 49.815 - 72.208 | 53.859 - 79.167 | 52.715 - 73.728 | 55.508 - 75.625 | 51.372 - 74.437 | 50.658 - 78.698 |
| **Epididymides / Term BW (%)** | Male | Mean | 0.244 | 0.259 | 0.256 | 0.268 | 0.247 | 0.260 |
|  |  | 95% CI | 0.228 - 0.260 | 0.243 - 0.275 | 0.239 - 0.272 | -- | -- | -- |
|  |  | Range | 0.192 - 0.309 | 0.202 - 0.328 | 0.195 - 0.320 | 0.206 - 0.330 | 0.187 - 0.298 | 0.198 - 0.327 |

**Table S12. Organ Weight, Organ to Terminal Body Weight, and Organ to Brain Weight Results for Rats fed Maize Source Diets (continued)**

|  | **Sex** | **Statistical Parameter** | **Control** | **DP23211**  **High** | **DP23211**  **Low** | **P0928^a^** | **P0993** | **P1105** |
| --- | --- | --- | --- | --- | --- | --- | --- | --- |
| **Heart**  **(g)** | Female | Mean | 0.974 | 0.986 | 0.970 | 0.960 | 0.966 | 0.972 |
|  |  | 95% CI | 0.903 - 1.046 | 0.914 - 1.058 | 0.898 - 1.041 | -- | -- | -- |
|  |  | Range | 0.848 - 1.204 | 0.836 - 1.278 | 0.793 - 1.148 | 0.888 - 1.102 | 0.774 - 1.253 | 0.860 - 1.159 |
|  | Male | Mean | 1.731 | 1.684 | 1.754 | 1.666 | 1.655 | 1.758 |
|  |  | 95% CI | 1.638 - 1.824 | 1.590 - 1.777 | 1.660 - 1.847 | -- | -- | -- |
|  |  | Range | 1.494 - 2.053 | 1.316 - 1.922 | 1.421 - 2.189 | 1.391 - 2.085 | 1.410 - 1.927 | 1.416 - 2.070 |
| **Heart /**  **Brain Wt**  **(%)** | Female | Mean | 47.049 | 48.326 | 47.192 | 47.060 | 46.037^b^ | 48.108 |
|  |  | 95% CI | 44.236 - 49.863 | 45.513 - 51.140 | 44.379 - 50.006 | -- | -- | -- |
|  |  | Range | 40.887 - 54.727 | 40.987 - 61.680 | 36.443 - 53.099 | 40.915 - 53.962 | 36.050 - 62.245 | 41.559 - 56.592 |
|  | Male | Mean | 77.209 | 74.846 | 76.203 | 73.667 | 72.974 | 77.925 |
|  |  | 95% CI | 72.488 - 81.930 | 70.125 - 79.567 | 71.482 - 80.924 | -- | -- | -- |
|  |  | Range | 67.860 - 91.816 | 57.093 - 90.125 | 59.481 - 88.552 | 65.551 - 84.825 | 63.888 - 81.514 | 62.242 - 94.270 |
| **Heart /**  **Term BW**  **(%)** | Female | Mean | 0.334 | 0.354 | 0.336 | 0.339 | 0.340 | 0.336 |
|  |  | 95% CI | 0.319 - 0.348 | 0.340 - 0.369 | 0.321 - 0.351 | -- | -- | -- |
|  |  | Range | 0.272 - 0.412 | 0.324 - 0.423 | 0.288 - 0.378 | 0.290 - 0.392 | 0.297 - 0.401 | 0.279 - 0.405 |
|  | Male | Mean | 0.296 | 0.293 | 0.294 | 0.300 | 0.290 | 0.312 |
|  |  | 95% CI | 0.281 - 0.310 | 0.279 - 0.308 | 0.280 - 0.309 | -- | -- | -- |
|  |  | Range | 0.268 - 0.353 | 0.245 - 0.341 | 0.255 - 0.344 | 0.263 - 0.328 | 0.253 - 0.341 | 0.230 - 0.463 |
| **Kidneys**  **(g)** | Female | Mean | 2.078 | 1.986 | 2.056^b^ | 2.009 | 1.981 | 1.975^b^ |
|  |  | 95% CI | 1.959 - 2.196 | 1.868 - 2.104 | 1.935 - 2.177 | -- | -- | -- |
|  |  | Range | 1.698 - 2.456 | 1.589 - 2.286 | 1.664 - 2.376 | 1.803 - 2.307 | 1.620 - 2.297 | 1.739 - 2.358 |
|  | Male | Mean | 3.480 | 3.486 | 3.621 | 3.509 | 3.528 | 3.351 |
|  |  | 95% CI | 3.325 - 3.634 | 3.332 - 3.641 | 3.466 - 3.775 | -- | -- | -- |
|  |  | Range | 2.838 - 4.083 | 3.076 - 3.967 | 2.969 - 3.993 | 2.951 - 4.813 | 3.059 - 4.219 | 2.530 - 3.978 |
| **Kidneys /**  **Brain Wt**  **(%)** | Female | Mean | 100.394 | 97.253 | 99.857^b^ | 98.442 | 93.835^b^ | 98.347^b^ |
|  |  | 95% CI | 94.259 - 106.529 | 91.118 - 103.388 | 93.610 - 106.105 | -- | -- | -- |
|  |  | Range | 82.708 - 120.747 | 78.199 - 111.639 | 82.675 - 118.504 | 81.695 - 115.697 | 74.006 - 111.722 | 85.079 - 114.912 |
|  | Male | Mean | 155.068 | 154.800 | 157.514 | 155.111 | 155.608 | 148.353 |
|  |  | 95% CI | 147.617 - 162.519 | 147.349 - 162.251 | 150.062 - 164.965 | -- | -- | -- |
|  |  | Range | 132.927 - 180.453 | 132.643 - 177.039 | 124.018 - 178.492 | 135.243 - 218.872 | 135.502 - 190.131 | 114.843 - 172.957 |
| **Kidneys /**  **Term BW**  **(%)** | Female | Mean | 0.710 | 0.713 | 0.716^b^ | 0.708 | 0.699 | 0.685^b^ |
|  |  | 95% CI | 0.678 - 0.743 | 0.681 - 0.746 | 0.682 - 0.749 | -- | -- | -- |
|  |  | Range | 0.601 - 0.790 | 0.629 - 0.802 | 0.587 - 0.823 | 0.610 - 0.791 | 0.636 - 0.821 | 0.572 - 0.783 |
|  | Male | Mean | 0.596 | 0.608 | 0.610 | 0.633 | 0.618 | 0.591 |
|  |  | 95% CI | 0.564 - 0.629 | 0.575 - 0.641 | 0.578 - 0.643 | -- | -- | -- |
|  |  | Range | 0.486 - 0.698 | 0.534 - 0.672 | 0.498 - 0.691 | 0.501 - 0.954 | 0.542 - 0.738 | 0.520 - 0.697 |
| **Liver**  **(g)** | Female | Mean | 8.121 | 7.725 | 7.676 | 7.580 | 7.363 | 7.919 |
|  |  | 95% CI | 7.520 - 8.723 | 7.123 - 8.326 | 7.075 - 8.278 | -- | -- | -- |
|  |  | Range | 6.867 - 9.487 | 5.918 - 9.645 | 5.838 - 9.232 | 6.624 - 8.778 | 6.242 - 8.595 | 6.676 - 9.840 |
|  | Male | Mean | 14.370 | 13.995 | 14.773 | 13.863 | 14.068 | 13.517 |
|  |  | 95% CI | 13.512 - 15.228 | 13.138 - 14.853 | 13.915 - 15.630 | -- | -- | -- |
|  |  | Range | 10.332 - 16.887 | 11.860 - 16.808 | 12.010 - 18.626 | 11.034 - 17.186 | 10.434 - 17.173 | 10.112 - 16.244 |
| **Liver /**  **Brain Wt**  **(%)** | Female | Mean | 392.665 | 378.190 | 373.865 | 371.545 | 350.100^b^ | 391.731 |
|  |  | 95% CI | 364.587 - 420.743 | 350.112 - 406.267 | 345.787 - 401.943 | -- | -- | -- |
|  |  | Range | 334.486 - 465.727 | 309.892 - 462.147 | 268.290 - 455.148 | 300.272 - 440.221 | 290.731 - 426.975 | 335.477 - 488.823 |
|  | Male | Mean | 640.779 | 622.169 | 641.319 | 611.622 | 619.634 | 598.546 |
|  |  | 95% CI | 599.933 - 681.625 | 581.323 - 663.015 | 600.472 - 682.165 | -- | -- | -- |
|  |  | Range | 466.035 - 755.233 | 514.534 - 732.166 | 501.671 - 790.103 | 505.683 - 746.244 | 472.768 - 726.438 | 459.010 - 723.563 |
| **Liver /**  **Term BW**  **(%)** | Female | Mean | 2.774 | 2.773 | 2.652 | 2.665 | 2.601 | 2.734 |
|  |  | 95% CI | 2.634 - 2.914 | 2.634 - 2.913 | 2.513 - 2.792 | -- | -- | -- |
|  |  | Range | 2.432 - 3.262 | 2.292 - 3.123 | 2.333 - 3.099 | 2.405 - 2.955 | 2.274 - 3.090 | 2.377 - 3.345 |
|  | Male | Mean | 2.445 | 2.436 | 2.468 | 2.485 | 2.457 | 2.373 |
|  |  | 95% CI | 2.357 - 2.533 | 2.348 - 2.525 | 2.380 - 2.556 | -- | -- | -- |
|  |  | Range | 2.062 - 2.881 | 2.204 - 2.652 | 2.250 - 2.859 | 2.163 - 2.878 | 2.132 - 2.874 | 2.187 - 2.554 |

**Table S12. Organ Weight, Organ to Terminal Body Weight, and Organ to Brain Weight Results for Rats fed Maize Source Diets (continued)**

|  | **Sex** | **Statistical Parameter** | **Control** | **DP23211**  **High** | **DP23211**  **Low** | **P0928^a^** | **P0993** | **P1105** |
| --- | --- | --- | --- | --- | --- | --- | --- | --- |
| **Spleen**  **(g)** | Female | Mean | 0.573 | 0.553 | 0.523 | 0.566 | 0.540 | 0.541 |
|  |  | 95% CI | 0.529 - 0.616 | 0.509 - 0.597 | 0.480 - 0.567 | -- | -- | -- |
|  |  | Range | 0.470 - 0.696 | 0.428 - 0.637 | 0.422 - 0.695 | 0.374 - 0.695 | 0.387 - 0.852 | 0.456 - 0.633 |
|  | Male | Mean | 0.942 | 0.909 | 0.947 | 0.936 | 0.809 | 0.907 |
|  |  | 95% CI | 0.859 - 1.026 | 0.826 - 0.993 | 0.863 - 1.030 | -- | -- | -- |
|  |  | Range | 0.652 - 1.241 | 0.704 - 1.273 | 0.741 - 1.283 | 0.709 - 1.312 | 0.647 - 1.086 | 0.713 - 1.137 |
| **Spleen /**  **Brain Wt**  **(%)** | Female | Mean | 27.661 | 27.150 | 25.445 | 27.744 | 25.786^b^ | 26.728 |
|  |  | 95% CI | 25.716 - 29.606 | 25.205 - 29.096 | 23.500 - 27.391 | -- | -- | -- |
|  |  | Range | 23.263 - 34.020 | 20.342 - 31.187 | 20.515 - 32.768 | 16.946 - 35.788 | 18.805 - 42.685 | 23.145 - 30.862 |
|  | Male | Mean | 42.101 | 40.384 | 41.082 | 41.332 | 35.654 | 40.164 |
|  |  | 95% CI | 38.337 - 45.864 | 36.620 - 44.148 | 37.319 - 44.846 | -- | -- | -- |
|  |  | Range | 28.128 - 55.977 | 30.358 - 53.227 | 31.579 - 51.901 | 32.345 - 56.969 | 29.354 - 48.941 | 32.365 - 48.465 |
| **Spleen /**  **Term BW**  **(%)** | Female | Mean | 0.196 | 0.200 | 0.181 | 0.200 | 0.190 | 0.187 |
|  |  | 95% CI | 0.184 - 0.208 | 0.188 - 0.212 | 0.169 - 0.193 | -- | -- | -- |
|  |  | Range | 0.165 - 0.235 | 0.150 - 0.251 | 0.159 - 0.214 | 0.149 - 0.260 | 0.140 - 0.279 | 0.147 - 0.224 |
|  | Male | Mean | 0.161 | 0.158 | 0.158 | 0.168 | 0.142 | 0.160 |
|  |  | 95% CI | 0.149 - 0.173 | 0.146 - 0.170 | 0.146 - 0.170 | -- | -- | -- |
|  |  | Range | 0.121 - 0.214 | 0.137 - 0.201 | 0.131 - 0.182 | 0.135 - 0.234 | 0.114 - 0.190 | 0.127 - 0.198 |
| **Testes**  **(g)** | Male | Mean | 3.681 | 3.843 | 3.786 | 3.771 | 3.625 | 3.784 |
|  |  | 95% CI | 3.488 - 3.874 | 3.650 - 4.036 | 3.593 - 3.979 | -- | -- | -- |
|  |  | Range | 2.992 - 4.241 | 3.448 - 4.936 | 3.070 - 4.312 | 3.218 - 4.382 | 3.110 - 4.442 | 3.263 - 4.327 |
| **Testes /**  **Brain Wt**  **(%)** | Male | Mean | 163.747 | 170.656 | 164.693 | 167.043 | 159.886 | 167.712 |
|  |  | 95% CI | 155.524 - 171.969 | 162.433 - 178.878 | 156.470 - 172.915 | -- | -- | -- |
|  |  | Range | 149.825 - 183.832 | 148.334 - 216.872 | 133.073 - 192.446 | 135.723 - 185.195 | 133.362 - 191.548 | 148.116 - 196.950 |
| **Testes /**  **Term BW**  **(%)** | Male | Mean | 0.631 | 0.673 | 0.639 | 0.684 | 0.637 | 0.669 |
|  |  | 95% CI | 0.590 - 0.672 | 0.631 - 0.714 | 0.597 - 0.680 | -- | -- | -- |
|  |  | Range | 0.481 - 0.735 | 0.576 - 0.914 | 0.527 - 0.747 | 0.493 - 0.810 | 0.486 - 0.767 | 0.561 - 0.742 |
| **Thymus**  **(g)** | Female | Mean | 0.306^b^ | 0.272 | 0.305 | 0.284 | 0.275^b^ | 0.293 |
|  |  | 95% CI | 0.270 - 0.341 | 0.237 - 0.306 | 0.270 - 0.339 | -- | -- | -- |
|  |  | Range | 0.237 - 0.380 | 0.143 - 0.408 | 0.207 - 0.434 | 0.133 - 0.455 | 0.151 - 0.400 | 0.201 - 0.442 |
|  | Male | Mean | 0.343 | 0.321 | 0.343 | 0.303 | 0.344 | 0.352 |
|  |  | 95% CI | 0.286 - 0.399 | 0.264 - 0.377 | 0.286 - 0.399 | -- | -- | -- |
|  |  | Range | 0.216 - 0.542 | 0.194 - 0.555 | 0.167 - 0.467 | 0.183 - 0.440 | 0.246 - 0.448 | 0.258 - 0.526 |
| **Thymus /**  **Brain Wt**  **(%)** | Female | Mean | 14.751^b^ | 13.354 | 14.805 | 13.926 | 13.256^c^ | 14.472 |
|  |  | 95% CI | 13.021 - 16.481 | 11.672 - 15.035 | 13.123 - 16.486 | -- | -- | -- |
|  |  | Range | 11.515 - 18.692 | 7.037 - 20.523 | 10.759 - 20.627 | 6.717 - 21.646 | 7.170 - 18.391 | 9.858 - 21.543 |
|  | Male | Mean | 15.381 | 14.232 | 14.939 | 13.383 | 15.118 | 15.614 |
|  |  | 95% CI | 12.779 - 17.983 | 11.631 - 16.834 | 12.338 - 17.541 | -- | -- | -- |
|  |  | Range | 9.647 - 27.141 | 8.366 - 24.183 | 7.469 - 22.049 | 7.784 - 17.901 | 11.146 - 18.951 | 11.336 - 22.870 |
| **Thymus /**  **Term BW**  **(%)** | Female | Mean | 0.104^b^ | 0.098 | 0.105 | 0.100 | 0.098^b^ | 0.101 |
|  |  | 95% CI | 0.095 - 0.114 | 0.088 - 0.107 | 0.095 - 0.114 | -- | -- | -- |
|  |  | Range | 0.085 - 0.126 | 0.057 - 0.149 | 0.078 - 0.133 | 0.048 - 0.154 | 0.055 - 0.140 | 0.066 - 0.155 |
|  | Male | Mean | 0.058 | 0.055 | 0.057 | 0.054 | 0.060 | 0.062 |
|  |  | 95% CI | 0.049 - 0.068 | 0.046 - 0.065 | 0.048 - 0.067 | -- | -- | -- |
|  |  | Range | 0.037 - 0.093 | 0.039 - 0.094 | 0.032 - 0.085 | 0.033 - 0.067 | 0.050 - 0.075 | 0.042 - 0.090 |
| **Ovaries**  **(g)** | Female | Mean | 0.142 | 0.145 | 0.145 | 0.146 | 0.137 | 0.142 |
|  |  | 95% CI | 0.130 - 0.154 | 0.133 - 0.157 | 0.133 - 0.157 | -- | -- | -- |
|  |  | Range | 0.103 - 0.179 | 0.112 - 0.197 | 0.118 - 0.201 | 0.109 - 0.174 | 0.089 - 0.183 | 0.110 - 0.167 |
| **Ovaries /**  **Brain Wt**  **(%)** | Female | Mean | 6.865 | 7.128 | 7.049 | 7.156 | 6.440^b^ | 7.016 |
|  |  | 95% CI | 6.318 - 7.411 | 6.581 - 7.674 | 6.503 - 7.596 | -- | -- | -- |
|  |  | Range | 4.790 - 8.995 | 5.648 - 9.439 | 5.775 - 9.297 | 5.359 - 8.744 | 4.325 - 8.632 | 5.261 - 8.069 |
| **Ovaries /**  **Term BW**  **(%)** | Female | Mean | 0.049 | 0.052 | 0.050 | 0.051 | 0.048 | 0.049 |
|  |  | 95% CI | 0.045 - 0.052 | 0.049 - 0.055 | 0.047 - 0.053 | -- | -- | -- |
|  |  | Range | 0.035 - 0.060 | 0.042 - 0.063 | 0.041 - 0.062 | 0.042 - 0.063 | 0.032 - 0.063 | 0.033 - 0.061 |

**Table S12. Organ Weight, Organ to Terminal Body Weight, and Organ to Brain Weight Results for Rats fed Maize Source Diets (continued)**

|  | **Sex** | **Statistical Parameter** | **Control** | **DP23211**  **High** | **DP23211**  **Low** | **P0928^a^** | **P0993** | **P1105** |
| --- | --- | --- | --- | --- | --- | --- | --- | --- |
| **Uterus^#^**  **(g)** | Female | Mean | 0.680 | 0.587 | 0.680 | 0.841 | 0.689 | 0.838 |
|  |  | 95% CI | 0.594 - 0.779 | 0.512 - 0.672 | 0.594 - 0.779 | -- | -- | -- |
|  |  | Range | 0.431 - 1.486 | 0.395 - 1.061 | 0.455 - 1.136 | 0.408 - 1.436 | 0.460 - 1.029 | 0.468 - 1.585 |
| **Uterus /**  **Brain Wt^#^**  **(%)** | Female | Mean | 31.613 | 28.048 | 31.974 | 41.283 | 33.128^b^ | 41.327 |
|  |  | 95% CI | 27.831 - 36.587 | 25.030 - 31.895 | 28.109 - 37.070 | -- | -- | -- |
|  |  | Range | 20.801 - 69.504 | 19.869 - 53.505 | 21.448 - 56.687 | 18.804 - 73.944 | 22.190 - 48.860 | 25.146 - 75.801 |
| **Uterus /**  **Term BW^#^**  **(%)** | Female | Mean | 0.224 | 0.206 | 0.227 | 0.297 | 0.243 | 0.288 |
|  |  | 95% CI | 0.198 - 0.259 | 0.183 - 0.234 | 0.200 - 0.263 | -- | -- | -- |
|  |  | Range | 0.147 - 0.523 | 0.145 - 0.416 | 0.151 - 0.402 | 0.148 - 0.537 | 0.155 - 0.377 | 0.180 - 0.509 |
| **Pituitary**  **(g)** | Female | Mean | 0.018 | 0.018 | 0.017 | 0.018 | 0.020 | 0.019 |
|  |  | 95% CI | 0.015 - 0.020 | 0.016 - 0.020 | 0.015 - 0.019 | -- | -- | -- |
|  |  | Range | 0.013 - 0.022 | 0.012 - 0.029 | 0.009 - 0.024 | 0.013 - 0.023 | 0.015 - 0.024 | 0.015 - 0.022 |
|  | Male | Mean | 0.015 | 0.016^b^ | 0.016 | 0.014 | 0.014 | 0.015 |
|  |  | 95% CI | 0.014 - 0.016 | 0.015 - 0.017 | 0.015 - 0.017 | -- | -- | -- |
|  |  | Range | 0.009 - 0.017 | 0.012 - 0.019 | 0.013 - 0.020 | 0.008 - 0.021 | 0.008 - 0.019 | 0.012 - 0.018 |
| **Pituitary**  **/ Brain Wt**  **(%)** | Female | Mean | 0.848 | 0.886 | 0.819 | 0.902 | 0.934^b^ | 0.919 |
|  |  | 95% CI | 0.752 - 0.944 | 0.790 - 0.983 | 0.723 - 0.915 | -- | -- | -- |
|  |  | Range | 0.633 - 1.029 | 0.606 - 1.378 | 0.459 - 1.092 | 0.669 - 1.153 | 0.724 - 1.140 | 0.713 - 1.093 |
|  | Male | Mean | 0.672 | 0.694^b^ | 0.694 | 0.637 | 0.636 | 0.676 |
|  |  | 95% CI | 0.622 - 0.721 | 0.643 - 0.745 | 0.645 - 0.743 | -- | -- | -- |
|  |  | Range | 0.417 - 0.851 | 0.538 - 0.900 | 0.571 - 0.900 | 0.347 - 0.893 | 0.359 - 0.852 | 0.522 - 0.807 |
| **Pituitary**  **/Term BW**  **(%)** | Female | Mean | 0.00602 | 0.00649 | 0.00585 | 0.00651 | 0.00705 | 0.00643 |
|  |  | 95% CI | 0.00532 - 0.00671 | 0.00579 - 0.00718 | 0.00516 - 0.00655 | -- | -- | -- |
|  |  | Range | 0.00460 - 0.00774 | 0.00489 - 0.01018 | 0.00372 - 0.00799 | 0.00486 - 0.00874 | 0.00506 - 0.00918 | 0.00469 - 0.00832 |
|  | Male | Mean | 0.00259 | 0.00273^b^ | 0.00270 | 0.00262 | 0.00253 | 0.00270 |
|  |  | 95% CI | 0.00236 - 0.00281 | 0.00250 - 0.00296 | 0.00247 - 0.00292 | -- | -- | -- |
|  |  | Range | 0.00156 - 0.00324 | 0.00203 - 0.00340 | 0.00194 - 0.00350 | 0.00123 - 0.00376 | 0.00160 - 0.00360 | 0.00214 - 0.00338 |
| **Thyroid**  **(g)** | Female | Mean | 0.020 | 0.019 | 0.018 | 0.018 | 0.017 | 0.018 |
|  |  | 95% CI | 0.018 - 0.022 | 0.017 - 0.021 | 0.016 - 0.020 | -- | -- | -- |
|  |  | Range | 0.014 - 0.026 | 0.015 - 0.024 | 0.012 - 0.023 | 0.013 - 0.025 | 0.013 - 0.022 | 0.008 - 0.026 |
|  | Male | Mean | 0.026 | 0.025 | 0.028 | 0.024 | 0.025 | 0.025^b^ |
|  |  | 95% CI | 0.025 - 0.028 | 0.023 - 0.027 | 0.026 - 0.029 | -- | -- | -- |
|  |  | Range | 0.020 - 0.041 | 0.019 - 0.030 | 0.021 - 0.038 | 0.015 - 0.036 | 0.019 - 0.033 | 0.019 - 0.037 |
| **Thyroid**  **/ Brain Wt**  **(%)** | Female | Mean | 0.963 | 0.937 | 0.863** | 0.901 | 0.826^b^ | 0.871 |
|  |  | 95% CI | 0.892 - 1.034 | 0.866 - 1.008 | 0.792 - 0.934 | -- | -- | -- |
|  |  | Range | 0.676 - 1.278 | 0.757 - 1.160 | 0.551 - 1.112 | 0.624 - 1.254 | 0.617 - 1.015 | 0.383 - 1.267 |
|  | Male | Mean | 1.180 | 1.107 | 1.197 | 1.043 | 1.101 | 1.118^b^ |
|  |  | 95% CI | 1.069 - 1.291 | 0.996 - 1.218 | 1.086 - 1.308 | -- | -- | -- |
|  |  | Range | 0.875 - 1.834 | 0.819 - 1.315 | 0.921 - 1.678 | 0.651 - 1.637 | 0.852 - 1.396 | 0.846 - 1.648 |
| **Thyroid**  **/Term BW***  **(%)** | Female | Mean | 0.00683 | 0.00688 | 0.00614** | 0.00648 | 0.00610 | 0.00602 |
|  |  | 95% CI | 0.00635 - 0.00731 | 0.00640 - 0.00736 | 0.00566 - 0.00662 | -- | -- | -- |
|  |  | Range | 0.00479 - 0.00813 | 0.00561 - 0.00865 | 0.00437 - 0.00749 | 0.00498 - 0.00828 | 0.00454 - 0.00768 | 0.00257 - 0.00813 |
|  | Male | Mean | 0.00448 | 0.00435 | 0.00465 | 0.00428 | 0.00437 | 0.00443^b^ |
|  |  | 95% CI | 0.00412 - 0.00484 | 0.00399 - 0.00471 | 0.00430 - 0.00501 | -- | -- | -- |
|  |  | Range | 0.00361 - 0.00566 | 0.00345 - 0.00539 | 0.00342 - 0.00707 | 0.00231 - 0.00713 | 0.00354 - 0.00552 | 0.00339 - 0.00582 |

Note: When a reported statistic was estimated to be < 10^-10^ in absolute value, the value was set to zero.

^a^	n=15 females only; ^b^ n=15; ^c^ n=14; otherwise n=16

^#^	Test and control data were transformed prior to analysis. The means are presented on the data scale (back-transformed), confidence interval (CI) are presented on the transformed scale. Reference arithmetic means were calculated from the raw data.

*	Sex x Treatment interaction statistically significant at the 0.05 level

**	Statistically significant difference when compared to control at the 0.05 level; FDR not significantly different

**Table S13. Summary of Microscopic Incidence Findings in Rats fed Maize Source Diets**

| **Finding^1^**  **(n = 16)** | **Female** | | | **Male** | | |
| --- | --- | --- | --- | --- | --- | --- |
|  | **Control** | **DP23211 High** | **DP23211 Low** | **Control** | **DP23211 High** | **DP23211 Low** |
| **Epididymides:** Infiltrate; lymphocytes, minimal | --- | --- | --- | 2 | 0 | 1 |
| **Eyes:** |  |  |  |  |  |  |
| Fold/rosette, retinal, minimal | 1 | 1 | 0 | 2 | 1 | 3 |
| Degeneration/atrophy, retinal | 0 | 0 | 0 | 2 | 1 | 0 |
| minimal  mild |  |  |  | [1]  [1] | 0  [1] |  |
| **Heart:** |  |  |  |  |  |  |
| Cardiomyopathy, minimal | 0 | 0 | 0 | 8 | 6 | 1 |
| Infiltrate: mononuclear, minimal | 0 | 0 | 0 | 1 | 3 | 4 |
| **Kidney** |  |  |  |  |  |  |
| Basophilic tubule(s), minimal | 0 | 0 | 0 | 2 | 2 | 3 |
| Chronic progressive nephropathy, minimal | 0 | 0 | 1 | 1 | 3 | 1 |
| Dilatation; unilateral, pelvis | 0 | 0 | 0 | 3 | 3 | 3 |
| minimal  mild  moderate |  |  |  | [3]  0  0 | 0  [2]  [1] | [2]  [1]  0 |
| Infiltrate; mononuclear | 5 | 2 | 2 | 3 | 1 | 3 |
| minimal  mild | [5]  0 | [2]  0 | [2]  0 | [3]  0 | [1]  0 | [2]  [1] |
| Mineralization, minimal | 0 | 2 | 0 | 0 | 0 | 0 |
| **Liver** |  |  |  |  |  |  |
| Inflammation, chronic, minimal | 0 | 0 | 0 | 2 | 2 | 0 |
| Vacuolation; median cleft, minimal | 4 | 4 | 2 | 3 | 3 | 3 |
| **Lungs** |  |  |  |  |  |  |
| Congestion | 1 | 1 | 0 | 1 | 2 | 2 |
| minimal  mild | [1]  0 | [1]  0 |  | [1]  0 | [1]  [1] | [1]  [1] |
| Infiltrate; histiocytic, minimal | 1 | 1 | 2 | 1 | 1 | 4 |
| Inflammation; chronic, minimal | 0 | 0 | 1 | 3 | 2 | 1 |
| **Lymph Nodes - Mandibular:** |  |  |  |  |  |  |
| Congestion | 0 | 0 | 0 | 3 | 1 | 1 |
| minimal  mild |  |  |  | [2]  [1] | [1]  0 | [1]  0 |
| **Lymph Nodes - Mesenteric:** |  |  |  |  |  |  |
| Congestion | 0 | 2 | 0 | 0 | 2 | 0 |
| minimal  mild |  | [2]  0 |  |  | [1]  [1] |  |
| **Pancreas** |  |  |  |  |  |  |
| Atrophy, acinar cell | 4 | 0 | 3 | 4 | 3 | 3 |
| minimal  mild | [4]  0 |  | [2]  [1] | [4]  0 | [3]  0 | [2]  [1] |
| Focus, basophilic, minimal | 0 | 0 | 0 | 0 | 2 | 0 |
| Infiltrate; mononuclear, minimal | 3 | 1 | 1 | 2 | 2 | 2 |
| Inflammation; chronic, minimal | 0 | 0 | 0 | 2 | 0 | 1 |
| **Prostate:** |  |  |  |  |  |  |
| Infiltrate; lymphocytes | - | - | - | 5 | 7 | 9 |
| minimal  mild |  |  |  | [5]  0 | [7]  0 | [8]  [1] |
| **Salivary Glands:** |  |  |  |  |  |  |
| Infiltrate, mononuclear, minimal | 2 | 0 | 0 | 0 | 0 | 1 |
| **Testes:** Atrophy | --- | --- | --- | 2 | 1 | 0 |
| minimal |  |  |  | [1] | [1] | 0 |
| moderate |  |  |  | [1] | 0 | 0 |
| **Thyroid Gland:** Ectopic thymus tissue, minimal | 1 | 1 | 0 | 2 | 2 | 0 |
| **Urinary Bladder:** Infiltrate; mononuclear, minimal | 1 | 0 | 1 | 2 | 4 | 1 |

^1^ This table represents findings that occurred at an incidence >1 in any treatment group evaluated.
